# Global climate impact of land-surface change driven by radiative effects

**DOI:** 10.1038/s41467-026-77834-0

**Published:** 2026-10-03

**Authors:** Philipp de Vrese, Victor Brovkin, Moritz Günther, Sarah M. Kang, Thomas Raddatz, Wim Thiery, Yi Yao

**Affiliations:** 1 Climate Dynamics, Max Planck Institute for Meteorology, Bundesstrasse 53, Hamburg, Germany; 2 Department of Water and Climate, Vrije Universiteit Brussel, Pleinlaan 2, Brussels, Belgium; 3 Institute for Atmospheric and Climate Science, ETH Zurich, Universitätstrasse 16, Zurich, Switzerland

**Keywords:** Hydrology, Climate change

## Abstract

A robust proportionality between radiative forcing and global-mean temperature has been established for most climate drivers. However, land-surface changes are considered an exception due to their potential to alter surface hydrology, introducing non-radiative effects that may rival those from surface albedo changes. Here, comparing Earth-system-model simulations with large-scale perturbations of surface albedo and water availability, we find that the spatially integrated biophysical climate impact is similarly dominated by radiative processes and scales approximately linearly with the resulting shifts in planetary albedo, implying a  ~ 0.4 K surface temperature response per W m^−2^ change in top-of-atmosphere net short-wave radiation. Thus, while non-radiative effects strongly influence local temperatures, their net contribution to the global-mean signal is negligible. The planetary-albedo response is primarily governed by regional cloud-cover characteristics, resulting in a substantially stronger temperature response to land-surface change in mid- and high-latitude regions than in the tropics and subtropics.

## Abstract (web summary)

This study shows that the integrated temperature response to land-surface change scales linearly with the resultant shift in planetary albedo, demonstrating that non-radiative effects — which determine local temperatures — cancel out at the global scale.

## Introduction

The most important physical surface properties shaping the land-atmosphere interactions are arguably its albedo and resistance to evapotranspiration, with the latter being closely linked to the availability of water. For both properties, we have an intuitive understanding of how their changes affect local temperatures, since we can directly relate to personal experience: Shifts in surface reflectivity modify the absorbed shortwave radiation, which is why dark surfaces often feel hotter on sunny days than bright ones. Water availability, on the other hand, determines the ratio of latent- and sensible heat, with the associated cooling being the main reason we perspire.

Numerous studies have investigated the ways land-surface change—generally understood as the alteration of the characteristics of Earth’s terrestrial surface via land-use and land-cover change – affects climate by modifying these surface properties^1–8^. Yet, the impact of man-made alterations to the surface hydrology—such as agricultural irrigation, or changes in evapotranspiration due to de- or afforestation—on global climate remains poorly constrained. They constitute the only major anthropogenic driver whose contribution is not fully accounted for in the assessments of the Intergovernmental Panel on Climate Change, owing to difficulties in quantifying associated non-radiative effects, which are thought to be of the same order of magnitude as those of surface-albedo changes^9–11^. One issue may be the expectation that the drivers of local effects, indeed often of a non-radiative nature^12–14^, are similarly responsible for the global-scale consequences. There is, however, a key difference in the way the surface properties shape regional and global climate: Changes in surface albedo modify Earth’s heat content directly, while changes in surface hydrology merely redistribute the available energy. And, without a direct effect on the overall energy, the spatially integrated climate impacts of changes in surface hydrology are determined by a number of indirect mechanisms whose temperature effects don’t necessarily align with the local response^15,16^.

The land-atmosphere exchange is shaped by highly complex, often non-linear dependencies. Nonetheless, the top-of-atmosphere (TOA) outgoing longwave radiation largely follows a linear relationship with the temperature of the underlying surface^17–21^. This suggests that changes in global-mean temperature relate to changes in Earth’s total longwave emissions in a similarly linear manner. Furthermore, while the efficacy of a forcing – i.e., the temperature response per unit TOA radiative forcing – depends on where the perturbation is applied^22–24^, the overall amount of short-wave radiation absorbed by a system in equilibrium must equal its long-wave emissions. Therefore, the equilibrium change in net TOA shortwave radiation (forcing and feedbacks) follows the same linear relationship with surface temperature as the outgoing longwave radiation. This, in turn, implies that the spatially integrated, long-term temperature response to changes in Earth’s physical surface properties is, regardless of type or location, simply a linear function of the resultant shift in planetary albedo. That is, unless non-radiative effects notably alter the TOA-radiation-surface-temperature relations[^9,10^, TOARSTR].

We test this hypothesis, comparing idealized simulations with the Max-Planck Institute for Meteorology’s Earth system model[^25^, MPI-ESM] in which we modified either the surface albedo or hydrology in three extensive continental regions, with an unperturbed control run. Here, we focus on changes that can be expected to induce a substantial local cooling, namely an increase in albedo of ~0.15 or a near-complete elimination of moisture constraints on evaporation, with changes being applied to the entire land surface in the latitudes 10°S–10°N, 15°N–35°N and 40°N–60°N, respectively.

## Results

### Effect on the surface energy balance

**Fig. 1:**
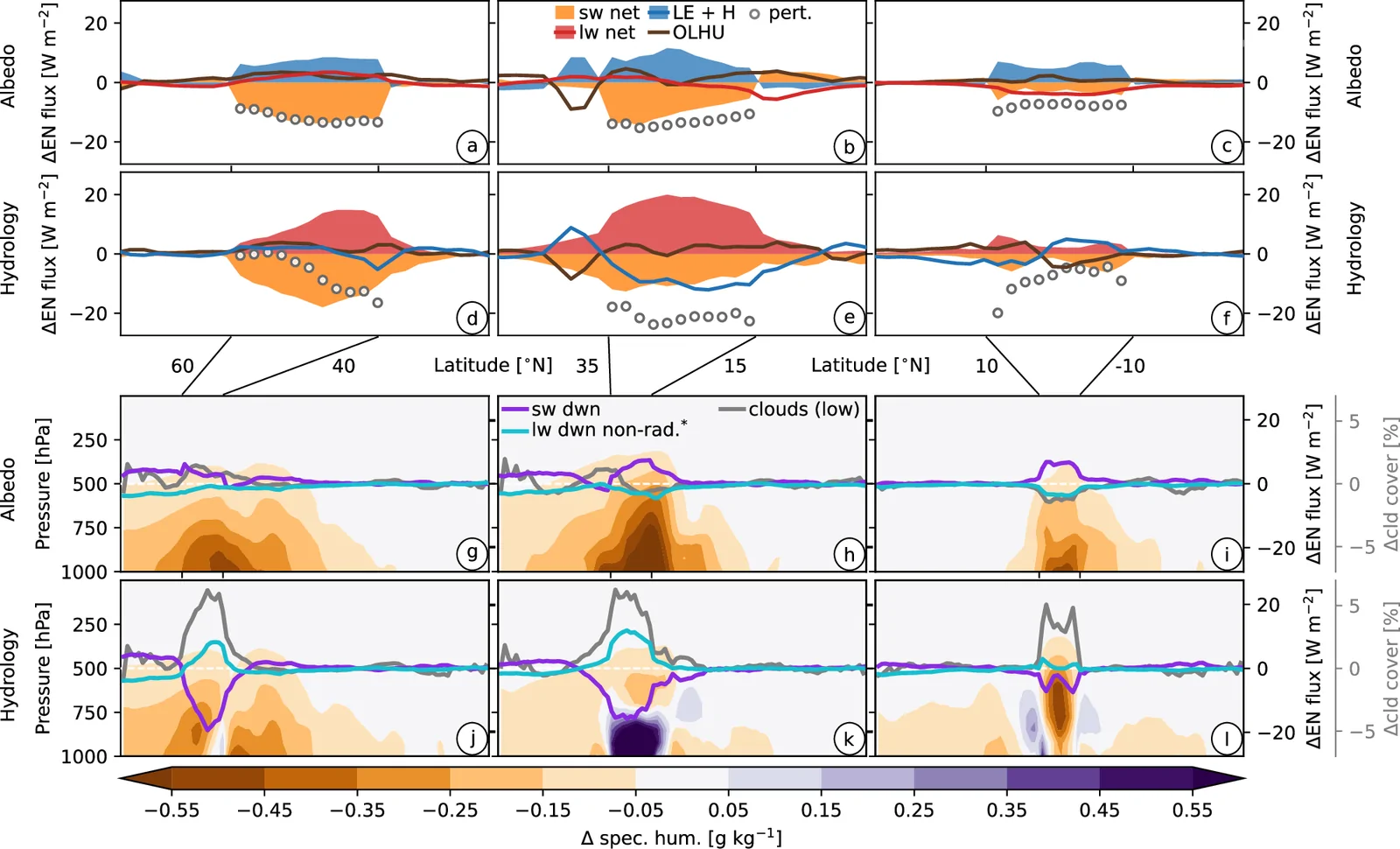
Surface energy balance. Panels **a**–**f** show 50-year mean, zonally averaged changes in the surface-energy fluxes (EN) resulting from increases in surface albedo (**a**–**c**) and water availability (**d**–**f**), with perturbed regions indicated on the x-axes. SW net is net surface shortwave radiation [orange], LW net is net surface longwave radiation [red], (H+LE) is the sum of turbulent fluxes [blue], and OLHU is ocean and land heat uptake [brown]. All fluxes are defined as positive downward except OLHU, which is defined as positive upward. Consequently, a positive value in the panels always corresponds to an increase of energy at the surface: 0 = sw net + lw net + (H + LE) + OLHU. Shading indicates the two dominant changes in the perturbed region, and solid lines indicate the less pronounced effects. Circles show the initial perturbation (pert.; either the prescribed albedo change multiplied by the incoming shortwave radiation from the control simulation [a-c] or the simulated change in latent heat flux [**d**–**f**]). Panels **g**–**l** show changes in specific humidity (shading) resulting from changes in surface albedo (**g**–**i**) and hydrology (**j**–**l**). Gray lines indicate changes in the low-altitude cloud cover (clouds (low); outer, right y-axis), purple lines in surface downwelling shortwave radiation (sw dwn; inner, right y-axis) and blue lines in the contribution of non-radiative processes to the atmospheric counterradiation (lw dwn non-rad.^*^; inner, right y-axis). The latter is approximated by comparing the simulated changes in counterradiation with temperature-based estimates (indicated by asterisks) that neglect changes in the atmospheric heat uptake due to changes in the turbulent fluxes and heat advection: $\Delta l{w}_{{{\rm{down}}}}^{{{\rm{SRF}}}*}=\Delta {{{\rm{lw}}}}_{{{\rm{up}}}}^{{{\rm{SRF}}}}-\Delta {{{\rm{lw}}}}_{{{\rm{out}}}}^{{{\rm{TOA}}}*}$. These make use of the linear dependency of the top-of-atmosphere (TOA) longwave emissions on surface (SRF) temperature: $\Delta {{{\rm{lw}}}}_{{{\rm{out}}}}^{{{\rm{TOA}}}*}=\Delta {T}^{{{\rm{SRF}}}}\cdot 1.7$, assuming fixed relationships between changes in surface temperature, in the greenhouse effect of water vapor and in atmospheric emissions due to changes in relative humidity^21^.

The system’s general response to surface perturbations differs notably between changes in albedo and hydrology (Fig. 1a–f and supplement SF5). Nonetheless, the decisive shifts in the surface energy balance are invariably driven by radiative processes, with the decreases in net surface shortwave (SW) radiation being the only factor that is consistent with the cooling in all simulations (sec. 2.2). Such a response agrees well with our understanding of albedo changes but not with the local effects of shifts in hydrology. The latter are dominated by the evaporative cooling associated with changes in the latent heat flux, hence, by non-radiative processes^12–14^. On larger spatial scales, however, the increase in the latent heat flux is offset by opposing shifts in the sensible heat fluxes, with the turbulent exchange often removing less energy from the surface (Fig. 1d, f and supplement SF3). This compensation arises mainly because evaporative cooling lowers the surface temperature while subsequent condensational heating warms the atmosphere, jointly reducing the surface-atmosphere temperature gradient that drives the sensible heat flux. And in case of a net increase in the turbulent fluxes, the resultant energy deficit is compensated by an increase in atmospheric counterradiation (Fig. 1e, k). As a result, the overall effect of non-radiative processes is largely limited to the redistribution of energy between unperturbed- and perturbed regions (Fig. 1g–l).

The changes in net sw radiation are a function of shifts in surface solar irradiance and -reflectivity, which for albedo increases constitute two opposing factors. Here, the increase in reflectivity reduces net SW radiation at the surface, resulting in general cooling. This cooling lowers the saturation vapor pressure, causing atmospheric drying and thus diminished SW absorption by water vapor, which in turn increases surface solar irradiance (Fig. 1g–i). Within the perturbed regions, the increase in surface reflectivity dominates, leading to a net reduction in SW radiation. However, because the drying of the atmosphere is not limited to these areas, some adjacent regions can exhibit an increase in net SW radiation. Importantly, the total reduction in atmospheric moisture content does not necessarily correspond to a decrease in relative humidity, often resulting in a spatially varying cloud response that both amplifies or diminishes the change in surface insolation.

For increases in water availability, the atmospheric response is spatially less uniform and specific humidity may increase locally (Fig. 1j–l). Nonetheless, the overall effect is also dominated by a cooling-related moisture reduction, implying that the changes in surface irradiance are not driven by an increased atmospheric water absorption. Instead, the effects stem from the finite water-holding capacity of the atmosphere, which increases precipitation to match the higher evapotranspiration rates and thereby cloudiness. This effect is amplified by the reduction in the sensible heat flux, which simultaneously cools the atmosphere and results in a higher relative humidity—a process that may further contribute to cloud cover via enhanced lower troposphere stability^26,27^. Thus, hydrological changes affect the net SW radiation primarily via shifts in cloud radiative forcing, especially from optically thick, low-altitude clouds.

### Global temperature response

**Fig. 2:**
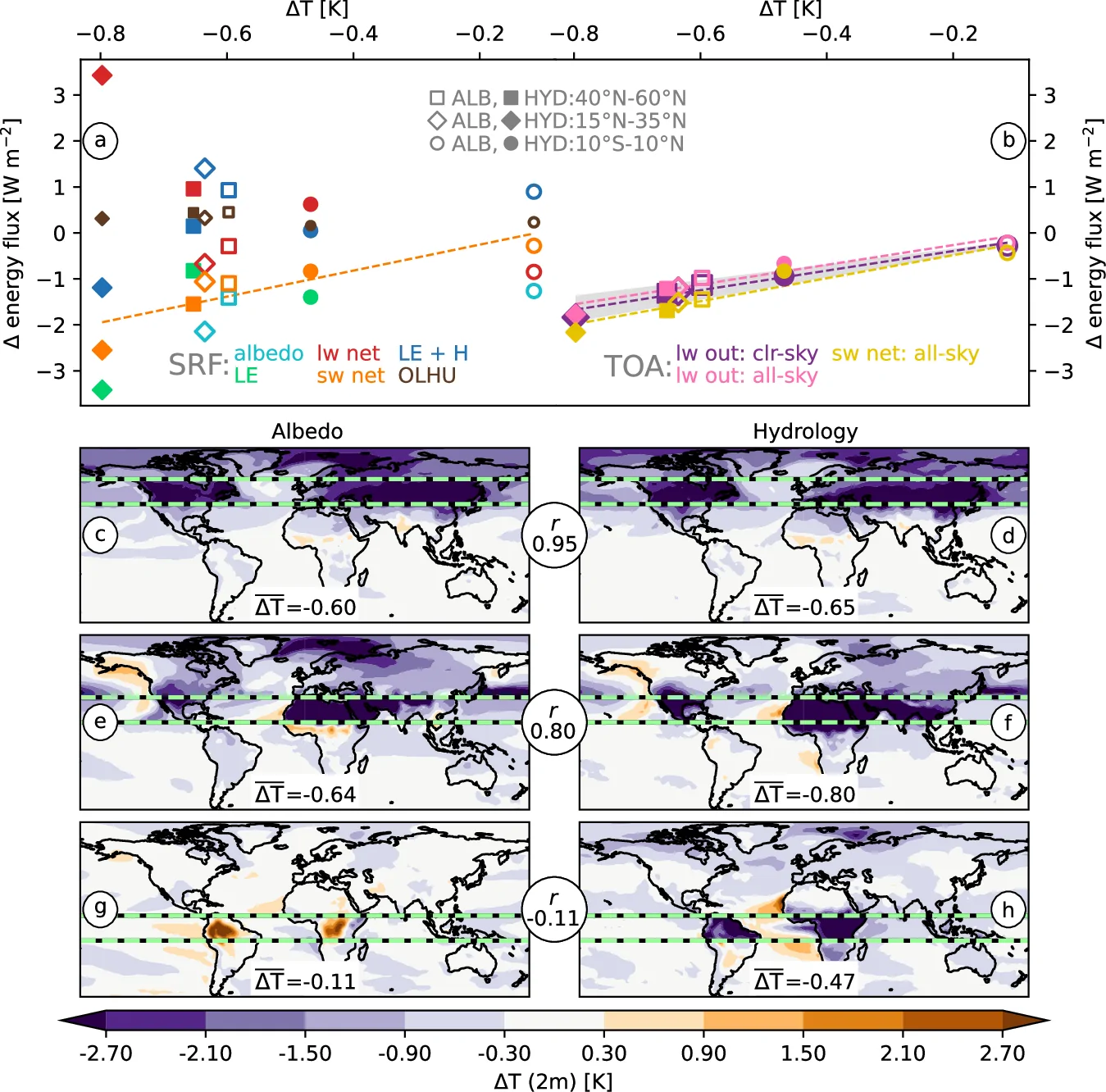
Temperature response. Panel **a** shows the global mean 2m air temperature change (Δ*T*) relative to the spatially integrated magnitude of a given albedo- (albedo) or hydrological (LE) perturbation and relative to the changes in the surface (SRF) energy-balance components, namely net lw-, net sw radiation at the surface (lw net, sw net), the turbulent fluxes (LE + H) and the ocean and land heat uptake (OLHU). Panel **b** shows the same but for the energy fluxes at the top of the atmosphere (TOA), namely clear-sky and all-sky outgoing lw fluxes (lw out: clr-sky, lw out: all-sky) and net sw radiation (sw net: all-sky). Colors indicate the energy balance component, markers indicate the experiments (open markers show albedo perturbations, full markers hydrological perturbations), and dashed lines indicate the best linear fit for statistically significant correlations. The gray shading in panel **b** indicates the observations- and reanalysis-based range of lw-TOARSTRs ± 10%^20,21^. Panels **c**, **e** and **g** show the temperature response to an increase in surface albedo in the regions between 40–60°N, 15–35°N and 10°S–10°N, respectively, and panels **d**, **f**, and **h** show the corresponding effects due to an increase in water availability. Dashed green lines indicate the latitudinal band of the perturbation, the numbers at the bottom of the panels indicate the global mean temperature response, and the number between two panels indicates the spatial correlation (coefficient *r*) between the two fields.

The global temperature response to changes in the surface properties is highly sensitive to the location of the perturbation (Fig. 2a): A smaller increase in latent heat flux in the region between 40–60°N results in a substantially larger cooling than in the region 10°S–10°N and there is a large discrepancy in the temperature response to comparable albedo perturbations between the equatorial and the two northern-hemisphere regions. Except for the net shortwave radiation, the surface energy balance components also exhibit a distinct lack of consistency in their relation to the global temperature response, with discrepancies stemming equally from differences in type and location of the perturbation.

The changes in spatially integrated TOA radiation, on the other hand, are highly consistent with the global temperature signal (Fig. 2b). Here, observations- and reanalysis-based estimates place the slope in the clear-sky lw-TOARSTR at between 1.9 and 2.2 W m^−2^ K^−1^ and suggest similar relationships for all-sky conditions^20,21^. For the simulated response to changes in the surface properties, none of the clear-sky LW-TOARSTRs deviates from this range by more than 10%, and there is no systematic difference between perturbations in different latitudes or between albedo- and hydrological perturbations. For all-sky lw-TOARSTRs, only the hydrological perturbation in the tropics falls slightly outside this range, indicating that, here, radiative effects associated with shifts in high-altitude cloud cover notably modify the TOARSTRs.

At the local level, changes in surface hydrology have a crucial non-radiative impact on the lw-TOARSTRs via unique lapse-rate effects (supplement SF7). Furthermore, there are important regional differences in the portioning of turbulent fluxes and long- and shortwave radiation—introducing distinct patterns in the zonally averaged response to surface perturbations which are currently being investigated in ongoing work. At the global level, however, the similarity to the lw-TOARSTRs for albedo changes—an entirely radiative disturbance—supports our initial hypothesis, suggesting that such non-radiative effects even out on this scale and play no major role in the overall climate impact of hydrological perturbations. Instead, a similar consistency in the sw-TOARSTRs—on average 2.5 W m^−2^ K^−1^—implies that the spatially integrated response to any large-scale change in the physical land-surface properties is driven by changes in planetary albedo, with the ocean heat uptake being the only other factor notably affecting the temperature signal (as indicated by the differences between lw- and sw-TOARSTRs). For changes in the surface hydrology, this means that the single most important factor determining the magnitude of the global temperature response is the radiative forcing from shifts in cloud cover, particularly at low altitudes (sec. 2.1, supplement SF8).

The similarities are not limited to the global-mean TOARSTRs, but there is also some consistency in the spatial temperature-change patterns (Fig. 2c–h). This suggests a comparable effect on large-scale circulations and remote regions, and adds to the findings of previous studies showing that these patterns are partly independent of the location of the perturbation^28,29^. Except for the tropical albedo increase (see below), all perturbations result in a notable cooling of the boreal region, the Arctic Ocean and the North Atlantic, mainly driven by changes in the meridional heat transport. At the same time, most northern-hemisphere perturbations lead to a warming at the southern border of the Sahel region and in South Asia, caused by changes in precipitation which are connected to a southward shift of the Intertropical Convergence Zone^30–32^. The marked horseshoe warming pattern (or less pronounced cooling) at North America’ west coast, on the other hand, stems from changes in the intensity and position of the Aleutian Low, which determines the strength of the westerly winds^33^.

For the tropical albedo increase, the picture is, however, very different. Here, a notable warming across the Amazon and Congo basins results from a secondary perturbation, namely an extreme decline in precipitation causing a substantial reduction in water availability and decrease in tree cover within the basins (supplements SF1 and SF5). This, in turn, affects evapotranspiration, resulting in a notable decrease in cloud cover and more shortwave radiation being absorbed at the surface despite the initial increase in the surface albedo. An albedo-change-induced breakdown of precipitation across these basins is, in itself, highly interesting^34,35^, but an in-depth discussion of this feature is beyond the scope of the present study. Nonetheless, the fact that the global TOARSTRs in this extreme case are not very different from the other perturbations implies another essential quality of land-surface changes, namely that synergistic effects are of minor importance for the global temperature response.

### Dependency on cloud cover

Our results suggest that (I) the global temperature mainly responds to shifts in planetary albedo, with the effect of land-surface changes on the latter being chiefly determined by the characteristics of the low-altitude cloud cover: Effects of changes in surface brightness scale with the fraction of the land surface visible from space, while hydrological perturbations predominantly affect the planetary albedo via the rate of cloud formation. The simulations also indicate that (II) remote effects are comparable between different perturbations and that (III) synergistic effects are small. It should, therefore, be possible to approximate the biophysical response to any land-surface change based exclusively on the local shifts in surface brightness and evapotranspiration in combination with region-specific sensitivity parameters that depend on the prevailing cloud-cover characteristics.

**Fig. 3:**
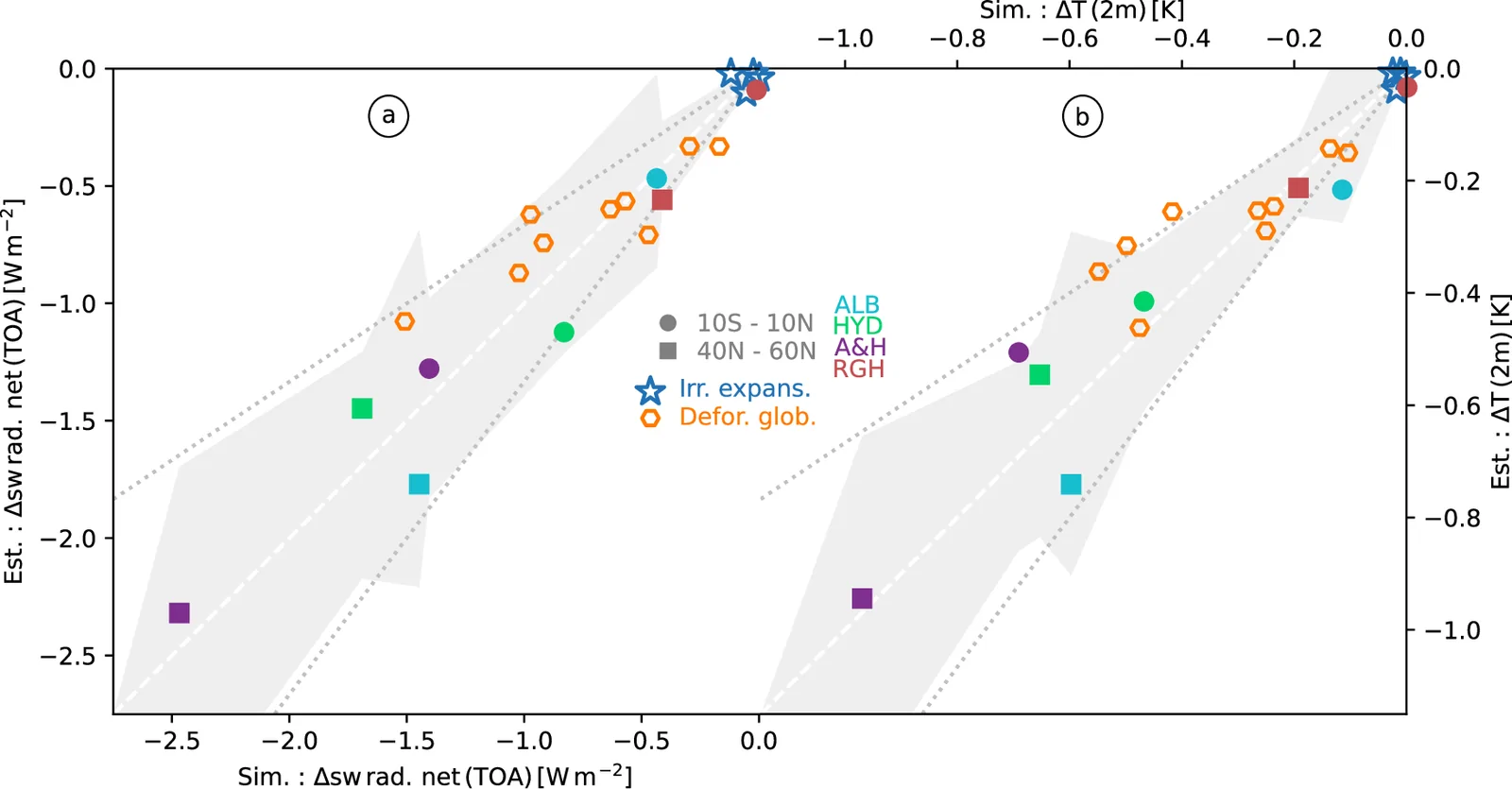
Approximation of the global temperature response. Shown is the comparison between the simulated (x-axis) and estimated (y-axis) global mean response to land-surface change. The estimates are based on the simulated shift in the (land) evapotranspiration and surface albedo within the latitudinal band of the initial perturbation, employing sensitivity factors that are based on the characteristics of the low-altitude cloud cover. Panel **a** shows the response in the top-of-atmosphere net shortwave radiation (Δsw. rad ne(TOA)), panel **b** that of the 2-meter temperature (ΔT (2m)). Cyan symbols refer to simulations with a change in surface albedo (ALB), green symbols to an increase in water availability (HYD), purple symbols to a combination of the two (A& H) and red colors to increases in surface roughness (RGH). Circles indicate perturbations in the region between 10°S-10°N and squares between 40–60°N (note that, due to the way the sensitivity parameters were determined, effects of perturbations in the region 15–35°N cannot be estimated). Dashed gray lines give the range of *y* = *x* ± ∣*x*∣/3, and the gray shading indicates the area *y* = *x* ± (∣*y*_alb_∣ + ∣*y*_evp_∣)/3, where *y*_alb_ constitutes the estimated effects of changes in surface albedo and *y*_evp_ those due to changes in evapotranspiration. Dark blue stars (Irr. expans.) show the response to the 20^th^-century expansion of irrigated areas as simulated by the models participating in the Irrigation Model Intercomparison Project (supplements SF9–SF11; note that due to the setup of the model intercomparison, we show the relations for the net shortwave fluxes at the surface instead of the top of the atmosphere). Orange hexagons show the results for the idealized deforestation scenario simulated in the Land Use Model Intercomparison Project (supplement SF12).

To test this hypothesis, we used such a simplified approach (Sec. 4, supplement ST2) to estimate the global temperature- and TOA net sw radiation response for a range of MPI-ESM simulations (the ones discussed above and simulations targeting a combination of brightness- and hydrological changes and an increase in surface roughness) as well as for multi-model experiments conducted in the Land-Use- and Irrigation Model Intercomparison Projects^36,37^. Overall, these estimates show a high agreement with the simulated responses (Fig. 3), suggesting the general validity of the assumptions (I–III) the simplified approach is based on. Here, the estimated impacts due to albedo- and hydrological perturbations exhibit minor biases, stemming mainly from the non-linear nature of the cloud sensitivities (supplement SF4). Yet the overall good agreement strongly suggests that the magnitude of the biophysical response to changes in these properties is, indeed, chiefly determined by regional cloud-cover characteristics. For the combination of brightness- and hydrological changes the agreement is even better (since the biases partly offset each other), resulting in a close fit between the estimated and simulated changes in the TOA radiative fluxes. Likewise, the temperature estimates deviate from the simulated values by less than a third of the latter which is as good an agreement as can be expected – considering the variability in the TOARSTRs (Fig. 2)—and confirms that synergistic effects play a minor role in the overall response. Furthermore, effects due to roughness changes are also approximated well, indicating that—similar to changes in water availability—they primarily act via shifts in Bowen ratio and cloudiness (supplement SF6).

In summary, our results thus strongly suggest that non-radiative effects make no meaningful contribution to the global temperature response to real-world land-use- and land-cover changes which, in the vast majority of cases, simultaneously alter surface brightness, -roughness and -hydrology. The response is, in fact, proportional to the resultant change in planetary albedo, with comparable surface perturbations in different regions exerting vastly different radiative forcings, depending on the prevailing cloud extent and cloud sensitivities.

## Discussion

Our investigation relies on simulations with a single Earth system model which limits the generalizability of some of our findings. Most importantly, the parameters describing the system’s sensitivity to albedo- and evapotranspiration changes are highly model-dependent (supplements SF11 and SF12), with the strength of cloud effects being a key source of uncertainty^38–40^. Yet, despite the large inter-model variability, we obtain very reasonable order-of-magnitude estimates of the biophysical climate response for other Earth system models, simulating large-scale deforestation scenarios and the 20^th^-century expansion of agricultural irrigation (Fig. 3, hexagons and stars). More importantly, the general shifts in the surface energy balance agree well with those produced by other models^41^, supplements SF9 and SF10, while the TOARSTRs are consistent with (quasi-) observation- and model-based estimates[^20,21,41,42^, supplement SF12]. We therefore believe the applicability of our key findings to be neither limited to our model nor to the idealized scenarios investigated in this study.

Instead, they offer straightforward answers to a number of longstanding questions, such as whether hydrological effects cause the forcing exerted by land-surface change to fundamentally differ from that of other drivers^9,10^. Here, our results show that non-radiative effects of shifts in surface hydrology – most importantly in the evaporative cooling—are balanced by changes in the sensible heat flux and counterradiation, while the overall temperature response stems primarily from changes in the low-altitude cloud cover. Therefore, treating the rapid cloud response as a radiative forcing instead of a climate feedback^43,44^—as done for cloud adjustments to aerosols^11,45,46^—would allow the radiative forcing framework to be applied to all aspects of land-surface change. Such treatment could also resolve the apparent difference between the climate sensitivity to land-use- and to atmospheric CO_2_ change^10,43^.

Our results also help resolve another important puzzle: why agricultural irrigation has profound local impacts yet only exerts a minor influence on global mean temperature^37,47–53^. Here, our findings suggest that we essentially irrigate in the “wrong" places for it to have a notable global-scale impact. Irrigated areas are predominantly located in arid regions^54^, where the local evaporative cooling may be strongest, but the cloud sensitivity to changes in evapotranspiration is comparatively low. Applying the same amount of water in the boreal region – with a weaker evaporative cooling but a substantially higher cloud sensitivity—would induce a global cooling that is several times larger, even suggesting that terrestrial water management offers some potential as a climate-mitigation tool.

## Methods

This study is based on simulations with the Max-Planck Institute for Meteorology’s Earth system model (MPI-ESM) using the standard versions of the atmospheric- and ocean components ECHAM6 and MPIOM. The model is largely identical to the one used in the sixth phase of the CMIP experiments^25,55^, but the present version includes a fix in the atmospheric component, where a bug was recently discovered that led to an error in the calculation of the transfer coefficients determining the turbulent exchange at the ocean-atmosphere interface. All modifications of the surface hydrology and albedo are limited to the land component JSBACH^56^ (the model version used in the present study is available under MPI-ESM software license version 2^57^, and model output has been archived via the German Climate Computing Center’s [DKRZ] long-term archive for documentation data: https://www.wdc-climate.de/ui/entry?acronym=DKRZ_LTA_1236_ds00002 [subject to CC BY 4.0]). Here, the modified code is only executed in the grid cells within predefined regions—the land surface within the latitudes between 10°S–10°N, 15–35°N and 40–60°N, respectively—using a mask that the model reads in together with the other boundary conditions.

For perturbations of the surface albedo we adjust the dynamically calculated values (${{{\rm{alb}}}}_{{{\rm{srf}}}}^{{{\rm{sim}}}}$), which include the effect of snow, by a prescribed offset (Δalb):

1 $${{{\rm{alb}}}}_{{{\rm{srf}}}}={{{\rm{alb}}}}_{{{\rm{srf}}}}^{{{\rm{sim}}}}+\Delta {{\rm{alb}}},$$

using an offset of +0.15, with the resulting albedo being limited to values between 0.03 and 0.9, for the visible range, and between 0.15 and 0.75 for the near-infrared range.

Changes in the water availability are represented in a similarly simplistic manner, by maintaining the water content of the skin reservoir (sr; the thin film of water that can form on top of any type of land surface) at a prescribed fraction (*f*_trgt_) of the maximum water content of the reservoir (${{{\rm{sr}}}}_{\max }$).

2 $${{\rm{sr}}}={{{\rm{sr}}}}_{\max }\cdot {f}_{\!\!{{\rm{trgt}}}}.$$

For simulations targeting an increase in water availability, we use a target fraction of 0.8, with adjustments limited to time-steps in which the dynamically simulated filling of the skin reservoir was below the target value and in which surface temperatures were above the freezing point. For simulations with a reduced water availability (required for the approximation of global effects), the target was 0.2, with adjustments being limited to time-steps in which this level was exceeded. It should be noted that, for simulations in which water was added to the skin reservoir, we did not fully balance the artificial fluxes, resulting in a marked violation of mass conservation. Here, the initial inputs ranged from ~0.4 Sv (10^6^ m^3^ s^−1^) to ~0.8 Sv, with the largest fluxes occurring in the 15–35°N region and the smallest in the 40–60°N domain. These rates are not minor modifications to the hydrological cycle but represent severe perturbations akin to those used in freshwater hosing experiments^58^. Therefore, in order to mitigate water balance violations, we attempted to compensate for the added water by reducing locally generated runoff and drainage in the perturbed regions. However, between ~0.2 Sv and ~0.6 Sv of water could not be balanced, of which ~0.05–~0.15 Sv entered the ocean via river discharge—though not necessarily within the perturbed latitudinal zones—with potentially important implications for simulated ocean circulation and heat uptake: The perturbations in the 10°S–10°N and 15–35°N regions led to a notable weakening of the Atlantic Meridional Overturning Circulation (AMOC), with a reduction of approximately ~2 Sv at 27°N. In contrast, the 40–60°N perturbation resulted in a strengthening of the AMOC by a similar magnitude. However, these patterns are not necessarily consistent with the expected signal for the freshwater forcing alone, which should have lead to a pronounced weakening of the AMOC, particularly when freshwater is added in the latitudes that include the regions of Atlantic deep water formation, that is between 40–60°N. Instead, the observed AMOC responses align more closely with changes in the latitudinal temperature gradient: cooling in high latitudes can be expected to strengthen the AMOC, while cooling closer to the equator may weaken it. Crucially, the simulations with albedo perturbations exhibited similar AMOC responses despite showing opposing changes in river discharge, suggesting that shifts in the latitudinal temperature gradient – rather than freshwater inputs—may indeed have been the dominant driver of AMOC variability in our experiments. The exception lies in the tropical regions, where hydrological and albedo perturbations produce markedly different AMOC responses—but here, the temperature responses also differ significantly between the two forcing types.

Furthermore, the unbalanced addition of water amplifies the model’s inherent inconsistencies in the treatment of the global energy balance. This is not merely a technical oversight but a consequence of deeper structural limitations present in the MPI-ESM framework. Here, river discharge is assumed to enter the ocean grid cell at the temperature of that cell, as the thermal state of water within rivers is never explicitly calculated. As a result, the model’s energy balance is not strictly closed. By adding or removing water at the surface, our perturbation approach exacerbates this existing thermodynamic inconsistency. However, while the water flux introduced by our adjustments is very large, the additional river runoff remains small relative to the total discharge to the ocean. Given that similar simplifications in the base model are considered acceptable for large-scale climate simulations, we assume that the additional energy flux from our perturbations remains within a comparable range of uncertainty. Nonetheless, we acknowledge that this assumption may not hold in all contexts-particularly at regional scales or over long integration periods-where cumulative effects could become significant.

All simulations were run at a T63 resolution, covering 50 years under pre-industrial greenhouse gas concentrations and starting from an equilibrium pi-control experiment. The effects due to any change in the land-surface properties are described by the (period) mean differences between the respective simulation and an unperturbed reference run. It should be noted that, in all experiments, the surface properties are partly dynamic because the model determines vegetation, snow, and sea-ice cover interactively. Consequently, the model includes vegetation-related feedbacks with respect to (i) surface albedo, (ii) resistance to evapotranspiration, and (iii) surface roughness. The only feedback that is not represented is the change in atmospheric CO_2_ concentrations that would result from vegetation changes, because CO_2_ is prescribed in all of our experiments. These feedback effects have important implications for how we define the initial perturbation in terms of energy, to make the perturbation strengths inter-comparable. For surface-albedo experiments we define the initial perturbation as the downwelling short-wave radiation in the unperturbed reference run, multiplied by the prescribed albedo offset. By construction, this definition excludes any albedo effects that arise from changes in vegetation or snow cover. For the water-availability experiments such a straightforward energy definition is problematic due to the way the perturbation is introduced in the model, that is by modifying the filling of the skin reservoir. Computing the perturbation as “added water x latent heat of vaporization" would be misleading, since the extra water may be routed to runoff rather than to evaporation. Instead we define the initial perturbation as the difference in latent-heat flux between the perturbed- and the control simulation, evaluated over the perturbed region. In a strict sense, this definition necessarily includes all surface feedbacks—especially those from vegetation—because the moisture flux into the atmosphere is also controlled by leaf area and stomatal conductance. Importantly, however, the amount of water added to the skin reservoir is also determined interactively and responds to the shifts in atmospheric water demand related to changes in transpiration; therefore any vegetation-induced increase or decrease in moisture flux is automatically compensated by the reservoir forcing. In practice this makes the changes in vegetation cover largely irrelevant for the hydrological perturbation.

With respect to the initial perturbation it should also be noted that the experiments were not designed to produce comparable global-mean effects. The three latitudinal bands differ markedly in continental area and in their background-climate states. Consequently, applying the same albedo offset or the same target filling of the skin-reservoir inevitably yields (i) different amounts of water added to the system and (ii) different changes in the global-mean surface albedo. In principle one could (a) adjust the widths of the latitudinal bands so that each band contains a similar land area, (b) restrict the perturbation to a subset of land grid-cells within a band, or (c) adjust the albedo offset and target filling to compensate for the differences in land area and climate within the perturbed regions. However, such “patterned” or size-adjusted perturbations may themselves modify the climate response (e.g., by altering the spatial distribution of the forcing and the associated feedbacks). For this reason we chose the simplest configuration: the same spatially uniform perturbation applied over the entire land surface within equal-width latitudinal bands. We acknowledge that a direct comparison of the absolute temperature responses between the three simulations is therefore of limited meaning. Instead, our analysis emphasizes qualitative differences in the regional responses and the resulting spatial patterns. When we present quantitative results we focus on the proportionality between the spatially integrated driver (e.g., perturbation strength or total change in planetary albedo) and the climate response, thereby removing the first-order effects of differing land-area fractions and background-climate states and enabling a meaningful comparison of the underlying physics across the three latitude bands.

In the last section (2.3), we attempt to estimate the climate impact of land-surface perturbations or combination of perturbations considering only the changes in the surface properties they entail (in case of water availability we consider the change in evapotranspiration directly), using sets of region-specific sensitivity parameters. These parameters are designed in a way that allows us to test a number of hypothesis that we make based on our analysis in the preceding sections. Our main hypothesis is that the overall (biophysical) climate impact (Δx) of any land surface change similarly arises due to and scales with the resulting change in planetary albedo (Δalb_TOA_). Here, our findings suggest that land surface changes notably modify the planetary albedo only via changes in surface brightness or in the extent of the low-altitude cloud cover, with changes in the latter being mainly determined by shifts in Bowen ratio. Therefore, the overall response to any land surface change is assumed to be proportional to the changes in surface albedo (Δalb_srf_) and evapotranspiration (Δevp) it entails, with the proportionality (*s*_x,evp_, *s*_x,alb_) being a function of the extent and sensitivity of the low-altitude cloud cover (cld):

3 $$\begin{array}{llllllr}\Delta {{\rm{x}}}&=&f(\Delta {{\rm{evp}}}\cdot {s}_{{{\rm{x}}},{{\rm{evp}}}},\Delta {{{\rm{alb}}}}_{{{\rm{srf}}}}\cdot {s}_{{{\rm{x}}},{{\rm{alb}}}})&&&&,{{\rm{with}}}\\ {s}_{{{\rm{x}}},{{\rm{evp}}}}&=&\frac{\Delta {{\rm{x}}}}{\Delta {{{\rm{alb}}}}_{{{\rm{TOA}}}}}\cdot f\left(\frac{\Delta {{\rm{cld}}}}{\Delta {{\rm{evp}}}}\right)\hfill&&&&,{{\rm{and}}}\\ {s}_{{{\rm{x}}},{{\rm{alb}}}}&=&\frac{\Delta {{\rm{x}}}}{\Delta {{{\rm{alb}}}}_{{{\rm{TOA}}}}}\cdot f\left({{\rm{cld}}},\frac{\Delta {{\rm{cld}}}}{\Delta {{{\rm{alb}}}}_{{{\rm{srf}}}}}\right).\hfill&&&&\end{array}$$

Furthermore, our results suggest, that synergistic effects are negligible in the relation of surface albedo, evapotranspiration and top-of-atmosphere shortwave radiation, so that the overall response should simply be the sum of the responses to albedo- and evapotranspiration changes:

4 $$\Delta {{\rm{x}}}=\Delta {{\rm{evp}}}\cdot {s}_{{{\rm{x}}},{{\rm{evp}}}}+\Delta {{{\rm{alb}}}}_{{{\rm{srf}}}}\cdot {s}_{{{\rm{x}}},{{\rm{alb}}}}.$$

Our results also suggest that non-local effects are largely independent of the type of perturbation and mainly arise due to change in the meridional heat transport. Thus, the non-local effects should be roughly proportional to the magnitude of the local temperature response and, therefore to the local changes in evapotranspiration (Δevp^local^) and surface albedo ($\Delta {{{\rm{alb}}}}_{{{\rm{srf}}}}^{{{\rm{local}}}}$).

5 $$\Delta {{\rm{x}}}=\Delta {{{\rm{evp}}}}^{{{\rm{local}}}}\cdot {s}_{{{\rm{x}}},{{\rm{evp}}}}\cdot (1+{f}_{{{\rm{non}}}-{{\rm{loc}}}})+\Delta {{{\rm{alb}}}}_{{{\rm{srf}}}}^{{{\rm{local}}}}\cdot {s}_{{{\rm{x}}},{{\rm{alb}}}}\cdot (1+{f}_{{{\rm{non}}}-{{\rm{loc}}}}),$$

with *f*_non−loc_, being the fraction by which the global response in- or decreases due to remote effects.

There is substantial spatial variability in the low-altitude cloud cover and the sensitivities, *s*_x,evp_, *s*_x,alb_, are, therefore, highly dependent on the latitudinal band (lat) in which the land surface change occurs. It is conceivable to deduce them based on the local climate conditions and known relations, such as the Clausius-Clapeyron equation. However, in the present investigation, we follow a different approach and determine the region-specific sensitivities (${s}_{{{\rm{x}}},{{\rm{evp}}}}^{{{\rm{lat}}}}$, ${s}_{{{\rm{x}}},{{\rm{alb}}}}^{{{\rm{lat}}}}$) based on reference sensitivities (${s}_{{{\rm{x}}},{{\rm{evp}}}}^{{{\rm{ref}}}}$, ${s}_{{{\rm{x}}},{{\rm{alb}}}}^{{{\rm{ref}}}}$) and region-specific adjustment factors (${f}_{{{\rm{evp}}}}^{{{\rm{lat}}}}$, ${f}_{{{\rm{alb}}}}^{{{\rm{lat}}}}$), all of which were determined based on model output. As reference (ref) we use simulations with perturbations prescribed in the region between 15–35°N, and we, therefore, do not attempt to estimate the climate response to the perturbations in this region in the MPI-ESM simulations. It should also be noted that the reference sensitivities already account for the non-local effects, i.e. that the scaling with (1 + *f*_non−loc_) is already included in ${s}_{{{\rm{x}}},{{\rm{evp}}}}^{{{\rm{ref}}}}$ and ${s}_{{{\rm{x}}},{{\rm{alb}}}}^{{{\rm{ref}}}}$:

6 $${s}_{{{\rm{x}}},{{\rm{evp}}}}^{{{\rm{lat}}}}={s}_{{{\rm{x}}},{{\rm{evp}}}}^{{{\rm{ref}}}}\cdot {f}_{{{\rm{evp}}}}^{{{\rm{lat}}}},{{\rm{and}}}\\ {s}_{{{\rm{x}}},{{\rm{alb}}}}^{{{\rm{lat}}}}={s}_{{{\rm{x}}},{{\rm{alb}}}}^{{{\rm{ref}}}}\cdot {f}_{{{\rm{alb}}}}^{{{\rm{lat}}}},{{\rm{with}}}\\ {f}_{{{\rm{evp}}}}^{{{\rm{lat}}}}=\frac{{\Delta }^{\star }{{{\rm{cld}}}}_{{{\rm{glb}}}}^{{{\rm{lat}}}}}{\Delta {{{\rm{evp}}}}_{{{\rm{lnd}}},{{\rm{lat}}}}^{{{\rm{lat}}}}}{\left[\frac{{\Delta }^{\star }{{{\rm{cld}}}}_{{{\rm{glb}}}}^{{{\rm{ref}}}}}{\Delta {{{\rm{evp}}}}_{{{\rm{lnd}}},{{\rm{ref}}}}^{{{\rm{ref}}}}}\right]}^{-1},{{\rm{and}}}\\ {f}_{{{\rm{alb}}}}^{{{\rm{lat}}}}=\left[(1-{{{\rm{cld}}}}_{{{\rm{lnd}}},{{\rm{lat}}}})\frac{{A}_{{{\rm{lnd}}},{{\rm{lat}}}}}{{A}_{{{\rm{glb}}}}}+\frac{{\Delta }^{\star }{{{\rm{cld}}}}_{{{\rm{lat}}}}^{{{\rm{lat}}}}}{\Delta {{{\rm{alb}}}}_{{{\rm{srf}}},{{\rm{lat}}}}^{{{\rm{lat}}}}}({{{\rm{alb}}}}_{{{\rm{cld}}}}-{{{\rm{alb}}}}_{{{\rm{srf}}},{{\rm{lat}}}})\frac{{A}_{{{\rm{lat}}}}}{{A}_{{{\rm{glb}}}}}\right]\\ \quad{\left[(1-{{{\rm{cld}}}}_{{{\rm{lnd}}},{{\rm{ref}}}})\frac{{A}_{{{\rm{lnd}}},{{\rm{ref}}}}}{{A}_{{{\rm{glb}}}}}+\frac{{\Delta }^{\star }{{{\rm{cld}}}}_{{{\rm{ref}}}}^{{{\rm{ref}}}}}{\Delta {{{\rm{alb}}}}_{{{\rm{srf}}},{{\rm{ref}}}}^{{{\rm{ref}}}}}({{{\rm{alb}}}}_{{{\rm{cld}}}}-{{{\rm{alb}}}}_{{{\rm{srf}}},{{\rm{ref}}}})\frac{{A}_{{{\rm{ref}}}}}{{A}_{{{\rm{glb}}}}}\right]}^{-1}.$$

Here, alb_cld_ is the cloud albedo (assumed to be 0.5) and *A* the surface area. Superscripts indicate the region of the initial perturbation and, in the formulation of the adjustment factors, subscripts indicate the spatial aggregation that is either across the entire globe (glb), the latitudinal zone (lat) in which the perturbation is applied or only the land area within the latitudinal band (lnd,lat). The values for the cloud cover (cld) and surface albedo (alb_srf_) stem from a simulation without land-surface perturbations, while the respective rates of change ($\frac{{\Delta }^{\star }{{\rm{cld}}}}{\Delta {{\rm{y}}}}$) stem from simulations with either an albedo- or a hydrological perturbation, isolating the respective effects in the output (see below).

As stated above, the surface properties in all simulations are, in parts, dynamic because the model determines the vegetation-, snow- and sea-ice cover interactively. This makes it difficult to isolate the effects that stem from a given albedo or hydrological perturbation from those resulting from local and remote changes in the other surface properties. Here, we adapted an iterative approach in which we initially determined the cloud sensitivities in simulations with a reduced water availability, which entailed only negligible effects on the surface albedo. Based on these cloud sensitivities, we were able to factor out the respective effects in simulations with prescribed changes in surface albedo, which were then used to isolate the changes in cloud cover resulting purely from changes in the surface hydrology. A more detailed description of the approach is given in the Supplementary Materials SF1 and SF2.

In this part of the analysis, we also attempt to estimate the climate effects due to changes in surface roughness (rgh), assuming that these can be approximated by the resulting changes in surface albedo, evapotranspiration and the corresponding sensitivity parameters. Here, the assumption is that, if roughness changes have any discernible large-scale effect, increases should lead to a surface cooling since they increase the turbulent exchange, hence, the amount of energy expended in the latent and sensible heat fluxes rather than in heating the surface. Thus, for the perturbed simulations, we simply double the roughness that the model simulates dynamically (${{{\rm{rgh}}}}_{{{\rm{sim}}}}$), with a minimum roughness increase of 2 m:

7 $${{\rm{rgh}}}=\left\{\begin{array}{ll}{{{\rm{rgh}}}}^{{{\rm{sim}}}}\cdot 2\quad &,{{\rm{if}}}\,{{{\rm{rgh}}}}^{{{\rm{sim}}}} > 2\\ {{{\rm{rgh}}}}^{{{\rm{sim}}}}+2\quad &{{\rm{otherwise}}}.\hfill\end{array}\right.$$

For bare and snow-covered areas, 2 m correspond to a 400- and 1000-fold increase, while this number is roughly 20 for grasslands and pastures.

## Supplementary information

Supplementary Information

Transparent Peer Review file

## Data Availability

The model output generated in this study has been deposited in the German Climate Computing Center’s (DKRZ) long-term archive for documentation data: https://www.wdc-climate.de/ui/entry?acronym=DKRZ_LTA_1236_ds00002 and is subject to the terms of the Creative Commons Attribution 4.0 International (CC BY 4.0). A minimum dataset is further available together with the code of the model (see Code availability). With a few key exceptions, all variables that have been investigated in this study are described in the user manual of MPI-ESM’s atmospheric component (ECHAM6^59^) and the technical documentation of its land model (JSBACH3^56^). These documents each contain a list of all output variables, including the information in which data stream they can be found, their GRIB code numbers and units. Importantly, relative humidity—a key factor in cloud formation, as highlighted in the manuscript—is included as a standard ECHAM6 model output (hur), while the strength of the Atlantic Meridional Overturning Circulation (AMOC) is available in MPIOM’s dedicated moc output stream. There are some derived variables which have to be determined as part of the postprocessing. Some of these variables, such as low-, mid-, and high-altitude cloud fraction can be computed with the ECHAM standard postprocessing, the Afterburner as part of the Climate Data Operators (CDO^60^), a few others have to be computed specifically from the ECHAM output: 1. planetary albedo = -srad0u / srad0d 2. surface albedo: = -sradsu/(srads-sradsu) 3. surface downwelling shortwave radiation = srads-sradsu 4. surface downwelling longwave radiation = trads-tradsu 5. top-of-atmosphere outgoing shortwave radiation = srad0d-srad0 6. Ocean and land heat uptake = srads + trads + ahfs + ahfl Finally, two additional output fields were implemented in the land model: the change in skin reservoir content resulting from the imposed hydrological perturbation: “skinres_chng_acc", and the unbalanced violation of the water-balance “wb_viol_acc". The first variable is given in kg m^−2^ s^−1^ and was assigned the GRIB code 229, while the second is also given in kg m^−2^ s^−1^ and has the GRIB code 230. Both variables can be found in the models “land" output stream.

## Code availability

The code of the reference model is subject to the license terms of the MPI-ESM license version 2 and available via Edmond, the Open Research Data Repository of the Max Planck Society https://doi.org/10.17617/3.H44EN5. The code used in this study is similarly available via Edmond https://doi.org/10.17617/3.JEDQZT, together with the configuration files and the files containing the boundary conditions required to repeat the experiments. Further, included is the minimal selection of model output, allowing to reproduce the analyses presented in the main article^57^.
