# Supplementary material for: Global climate impact of land-surface change driven by radiative effects
Source: Nat Commun. 2026 Oct 3;17:10454. doi: 10.1038/s41467-026-77834-0 (PMC13634050; doi:10.1038/s41467-026-77834-0)
Supplement: Supplementary file 1 [file 41467_2026_77834_MOESM1_ESM.pdf]

Supporting Information for **Global climate impact of land-surface change driven by radiative effects**

de Vrese, Brovkin, Günther, Kang, Raddatz, Thiery, Yao

Contents of this file

1. Figures SF1 to SF12
2. Tables ST1 and ST2

Supplementary Figures

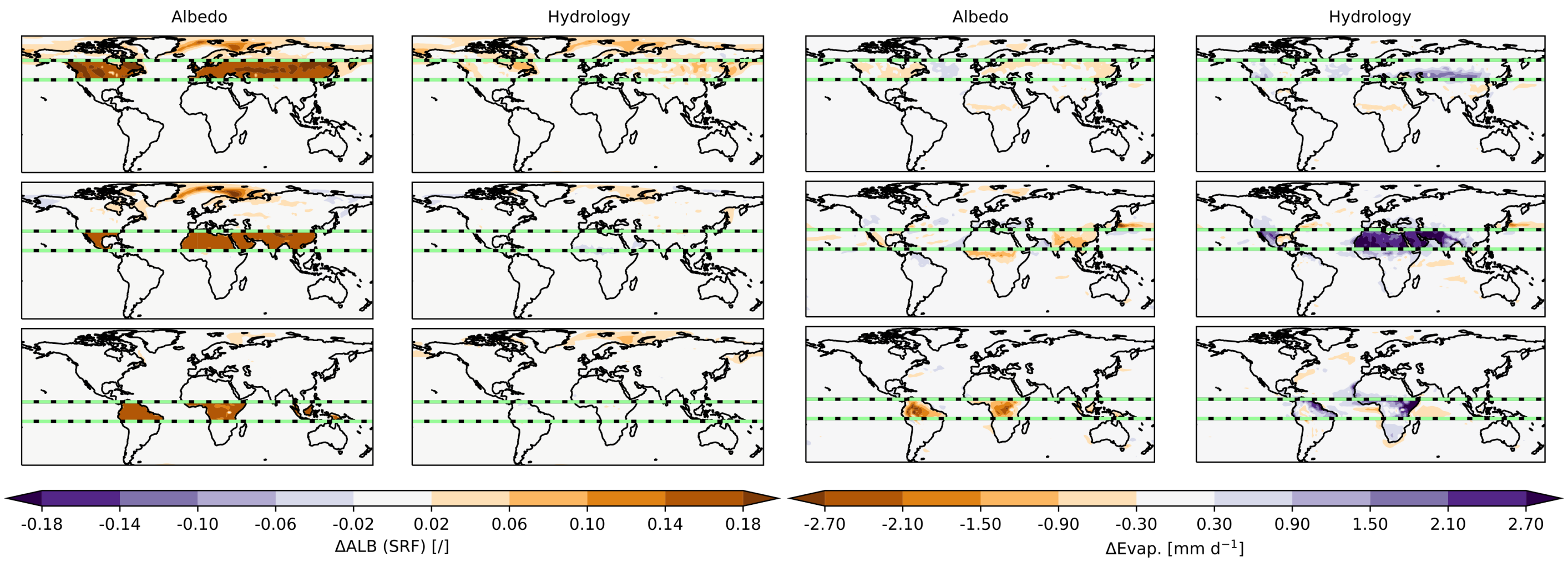

Figure SF1: **Change in surface albedo and evapotranspiration in response to initial perturbation (Part I)**

Shown are the changes in surface albedo, resulting from an increase in surface reflectivity (Albedo; first column) and water availability (Hydrology; third column) as well as the changes in evapotranspiration, due to an increase in surface reflectivity (Albedo; second column) and water availability (Hydrology; fourth column). Here, the first row shows the effects due to perturbations in the zone between 40°N-60°N, the second row between 15°N-35°N and the third row between 10°S-10°N, with the latitudinal zone of the initial perturbation indicated by dashed green lines. The initial albedo perturbation was simulated applying a fixed offset of 0.15 to the dynamically simulated values ($\Delta\text{alb} = +0.15$) and the change in water availability by maintaining the filling of the skin reservoir at a target fraction of at least 80% of the reservoirs maximum capacity ($f_{\text{trgt}} = 0.8$).

Any change in water availability leads to (remote) changes in the surface albedo and any change in surface brightness entails effects on the surface hydrology. In case of brightness increases (decreases), a reduction (increase) in evapotranspiration is part of the direct response, since the former reduces (increase) the available energy at the surface, hence, the energy contained in the turbulent fluxes. In contrast, changes in water availability mainly entail indirect effects on the surface albedo – via temperature-related changes in vegetation, snow cover and sea-ice extent. Furthermore, brightness changes may also have an indirect effect on evapotranspiration, in particular, if they alter precipitation patterns. In some cases, e.g. the changes in surface albedo due to the increase in water availability between 15°N-35°N, these secondary effects are so small that they can safely be neglected. However, in other cases, e.g. the evapotranspiration changes due to the albedo perturbations between 10°S-10°N they are in the same order of magnitude as those in the simulations in which they constitute initial perturbation (see below Tab. ST1).

The above issue poses a particular challenge for the last part of our analysis, which requires estimates of the cloud sensitivity for the individual surface properties. There are several ways to determine the cloud sensitivity to changes in a specific surface property. The simplest way is by defining it as the simulated change in cloud cover relative to the initial perturbation, including the impacts of all secondary effects. While this is certainly a valid and straight-forward approach, it did result in a poor agreement between estimates and simulated values, particularly for those perturbations that entail large secondary effects. A second possibility is to define the cloud sensitivity as the change in cloud cover relative to the initial perturbation, but trying to exclude the cloud cover changes due to all secondary effects. However, the complex nature of the Earth system makes it next to impossible to accurately separate direct from indirect effects. For the present investigation, we, therefore, used a different approach and determined the cloud sensitivity to changes in surface brightness based on the change in cloud cover as simulated for an albedo perturbation, but trying to remove all effects, direct and indirect, due to changes in evapotranspiration (SF2). Accordingly, the cloud sensitivity to changes in evapotranspiration was determined based on the change in cloud cover as simulated for a hydrological perturbation, but removing all effects, direct and indirect, due to changes in surface brightness.

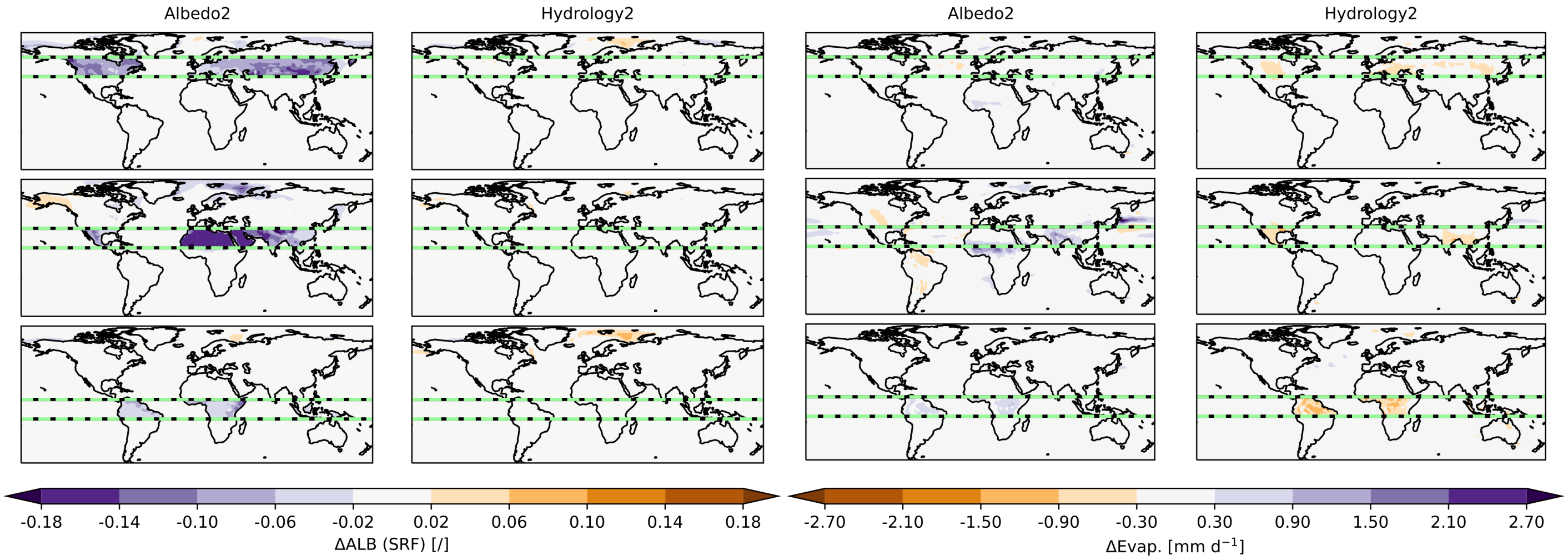

Figure SF2: **Change in surface albedo and evapotranspiration in response to initial perturbation (Part II)**

Shown are the changes in surface albedo, resulting from an decrease in surface reflectivity (Albedo2; first column) and water availability (Hydrology2; third column) as well as the changes in evapotranspiration, due to an decrease in surface reflectivity (Albedo2; second column) and water availability (Hydrology2; fourth column). Here, the first row shows the effects due to perturbations in the zone between 40°N-60°N, the second row between 15°N-35°N and the third row between 10°S-10°N, with the latitudinal zone of the initial perturbation indicated by dashed green lines. The initial albedo perturbation was simulated applying a fixed offset of -0.15 to the dynamically simulated values ($\Delta \text{alb} = -0.15$) and the change in water availability by maintaining the filling of the skin reservoir at a target fraction of at most 20% of the reservoirs maximum capacity ($f_{\text{trgt}} = 0.2$).

In addition to the simulations with an increase in surface brightness and water availability (SF1), we conducted two sets of simulations, with a decreased albedo and water availability. Based on these 6 sets of simulations we disaggregated the change in cloud cover into changes exclusively due to changes in surface albedo and changes resulting purely from shifts in evapotranspiration in the following manner:

1) Decreases in water availability entail only minor effects on the surface albedo (Hydrology2; second column) and it can be assumed that the latter contribute very little to the change in cloudiness. Thus, the cloud sensitivity to decreases in evapotranspiration can be estimated by the change in cloud cover in the respective simulations relative to the simulated decrease in evapotranspiration.
2) Increases in surface brightness almost exclusively lead to a decline in evapotranspiration (SF1; Albedo; third column), allowing us to estimate the corresponding effects on the cloud cover using the sensitivities determined in step 1. The latter can then be removed from the overall change in cloud cover, yielding the changes in cloud cover exclusively due to the increases in albedo.
3) Finally, the cloud sensitivities to increases in evapotranspiration can be isolated in the simulations with an increased water availability by removing the effects due to albedo increases (SF1; Hydrology; second column), using the sensitivities estimated in step 2 together with the simulated increases in surface brightness.

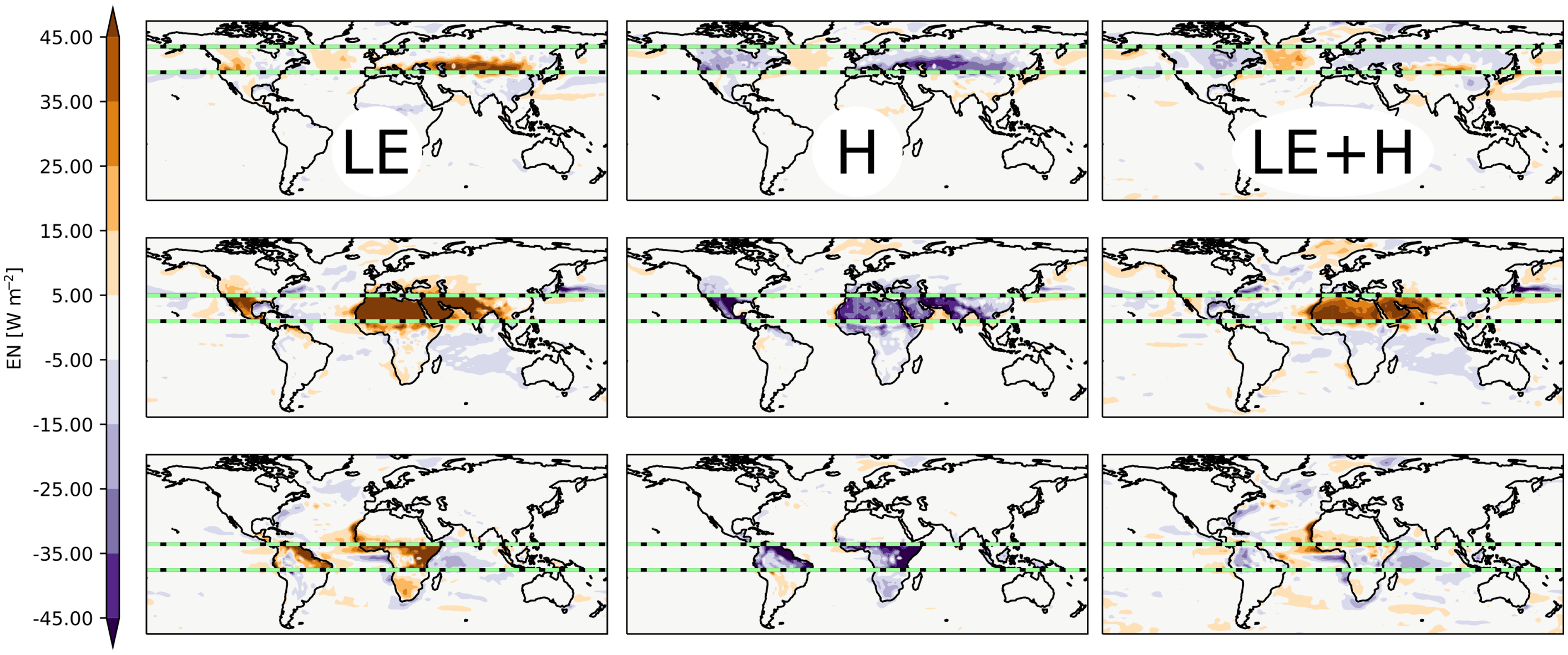

Figure SF3: **Compensatory shifts in LE and H in case of changes in water availability**

Shown are the 50-year mean changes in latent heat flux (LE, first column), sensible heat flux (H, second column) and in the overall turbulent fluxes (LE+H, third column), resulting from an increased water availability in the regions between 40°N-60°N (top row), 15°N-35°N (middle row) and 10°S-10°N (bottom row), with dashed green lines indicating the latitudinal band of the perturbation. Please note that, different from Fig. 1 in the main manuscript, all fluxes are defined as upward positive.

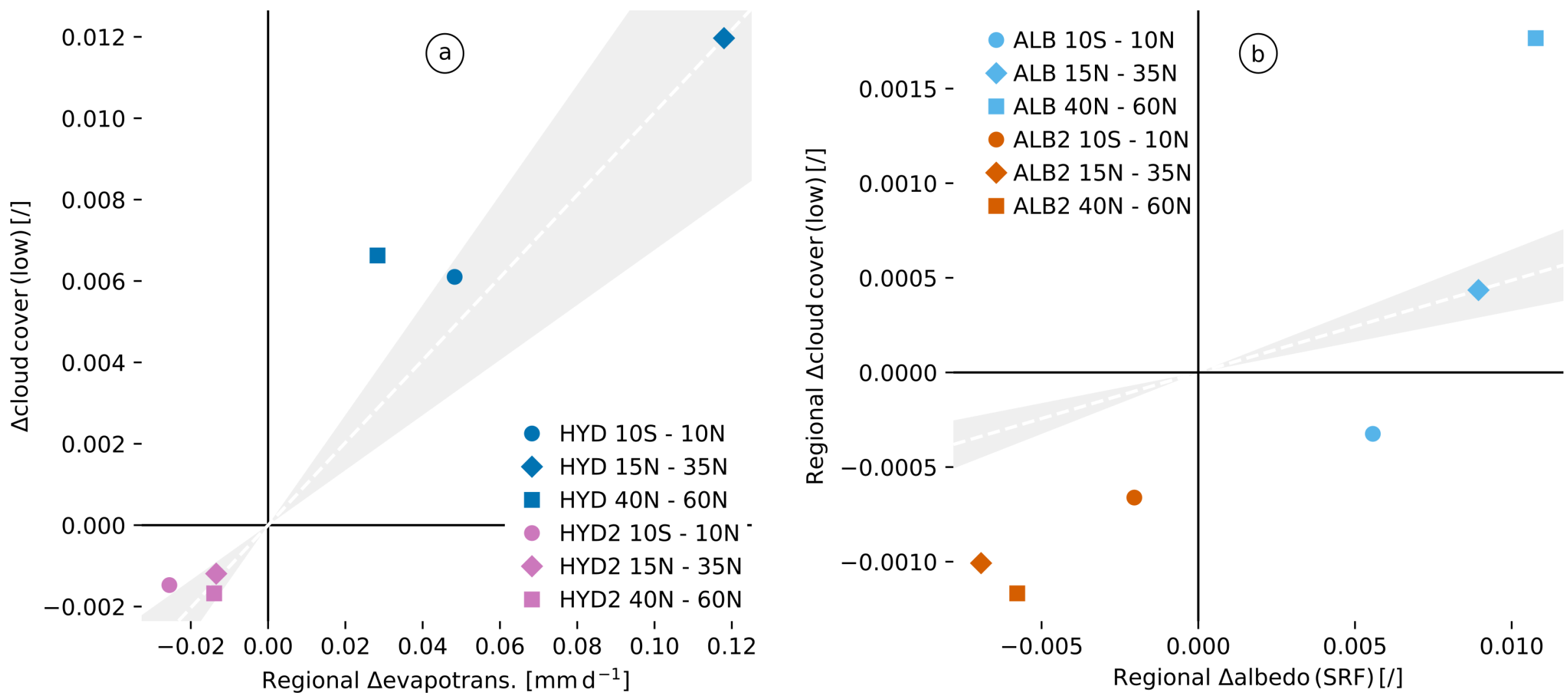

Figure SF4: **Cloud sensitivities**

Shown are the changes in low-altitude cloud cover resulting from changes in evapotransiration and surface albedo. Here, panel a shows the change in the global mean cloud cover relative to the change in terrestrial evapotranspiration within the perturbed region (weighted by the ratio of perturbed area and Earth's total surface area). The dark blue color refers to simulations in which the water availability was increased (HYD) and pink indicates simulations with a reduced water availability (HYD2). To isolate the effects of changes in evapotranspiration from those resulting from changes in surface albedo, we followed the approach described in SF2. Panel b shows the change of the cloud cover within the latitudinal zone of the perturbation relative to the change in the terrestrial surface albedo (weighted by the ratio of land area and total area within the perturbed latitudinal band). Light blue indicates simulations with an increased surface albedo (ALB) and red simulations with a decrease in albedo (ALB2). In both panels, circles indicate perturbations in the region between 10°S-10°N, diamonds between 15°N-35°N and squares between 40°N-60°N. The dashed white line shows (and extends) the connection between the origin of the graph and the data pair obtained for HYD15°N-35°N and ALB15°N-35°N and the gray shading gives the range of $y = x \pm \frac{|x|}{3}$ around the line.

The cloud sensitivities are highly dependent on the region of the land-surface perturbation, with generally higher sensitivities in latitudes that feature a more extensive cloud cover – that is a higher sensitivity in the region 40°N-60°N and 10°S-10°N than in the region between 15°N-35°N. Consequently the sensitivity parameters used in the estimation of global-scale climate impacts, are determined specifically for the region in which the change in surface properties occurs. The cloud sensitivities also depend on the direction of the change in the surface properties, with generally higher sensitivities to increases in water availability and decreases in surface albedo. Overall, this suggests a non-linear dependency of changes in cloud cover on the causative changes in surface properties. Such non-linearity has a large potential to introduce errors in the estimation of the global-scale climate effects, since the use of factors – rather than functions – implies that changes in cloud cover scale directly with the changes in surface properties. Here, the sensitivities are calculated separately for positive and negative changes in the surface properties, nonetheless, our approach still assumes that the cloud cover increases linearly with the magnitude of increases in water availability and surface albedo while decreases in cloud cover scale linearly with the magnitude of decreases in albedo and water availability.

The non-linear dependency of the cloud sensitivities to a changes in a given surface property does not only affect the final estimates of the global response, but also the estimates of the cloud sensitivity to changes in the other surface property. Our approach to determine the cloud sensitivities requires the attribution of simulated cloud-cover changes to either brightness- or hydrological changes (SF2). Thus, if the cloud sensitivity to albedo changes is (under-) overestimated, the sensitivity to changes in water availability is necessarily (over-) underestimated and vice versa. The effects due to inaccuracies in the attribution are clearly visible in the estimates of the global response (main article Fig. 3), where the biases in the estimates for albedo changes oppose those for hydrological perturbations. In the estimates for combined albedo and hydrological perturbations the two biases offset each other, resulting in a very close fit between estimated and simulated values, at least in case of the TOA radiative fluxes.

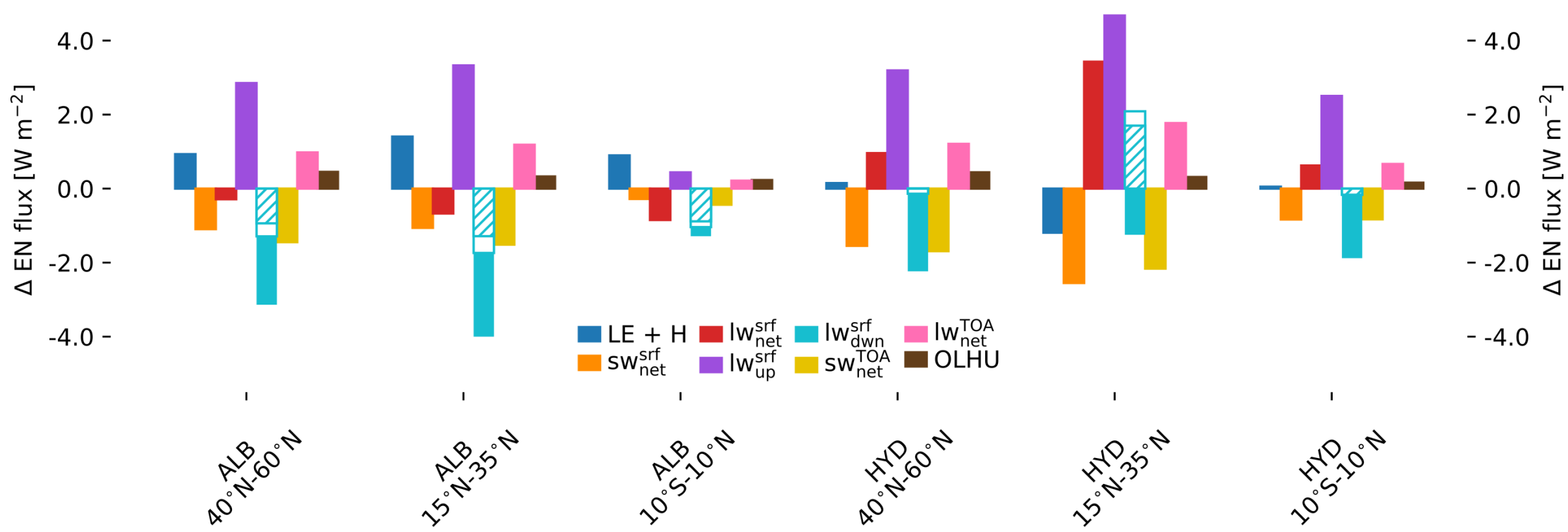

Figure SF5: **Effects on the global surface energy balance**

Shown are 50-year average, global-mean changes in the turbulent surface fluxes (LE + H, blue), the short-wave (sw_{net}^{srf}, orange) and long-wave (lw_{net}^{srf}, red) net radiation at the surface, the surface long-wave emissions (lw_{up}^{srf}, cyan), the counterradiation (lw_{dwn}^{srf}, grey), the net short-wave (sw_{net}^{TOA}, light green) and long-wave (lw_{net}^{TOA}, pink) radiation at the top of the atmosphere and the ocean and land heat flux (OLHU, brown). The changes result from albedo- (ALB) and hydrological (HYD) perturbations in 3 latitudinal zones, that is 10°S-10°N, 15°N-35°N and 40°N-60°N. Also show are the changes in counterradiation due to non-radiative effects (grey outline, grey hatches), calculated as described in Fig. 1 in the main article, and the changes in counterradiation that additionally include the effects of changes in sw absorption (grey outline, no hatches). Positive values indicate an increase in the energy content of the surface or at the top of the atmosphere.

All perturbations result in a reduction of the surface- and top-of-atmosphere (TOA) long-wave (lw) emissions, consistent with the overall cooling signal. Similarly, all simulations indicate a reduction in the TOA net short-wave (sw) fluxes, which together with the reduced ocean and land heat uptake balance the change in TOA lw emissions. Almost all simulations exhibit a reduced sw absorption in the atmosphere (indicated by the respective contribution to the counterradiation), consistent with the overall drying of the atmosphere. Here, the hydrological perturbation between 15°N-35°N is an exception, exhibiting an increase in sw absorption, despite the overall decrease in the atmospheric water content. The reason for this lies in the spatial distribution of water vapor and incident sw radiation, since the regions in which the atmospheric moisture content actually increases feature a higher irradiance than the regions with a reduced atmospheric water vapor (main manuscript Fig.1). In all the experiments, the atmosphere has an increased net absorption of lw radiation – given by the difference in lw_{up}^{srf} up and lw_{net}^{TOA} – mainly due to the non-linear dependency of lw emissions on temperature.

In contrast to the above similarities, the perturbations exhibit extremely different effects with respect to the surface net lw radiation, which is mainly due to the variability in the response of the counterradiation. The latter is a function of the changes in the surface lw emissions (since the largest fraction of these is absorbed within the atmosphere and partially re-emitted to the surface) and of the changes in sw absorption in the atmosphere, which for most perturbations corresponds to a decrease in the downward lw fluxes (since the dryer atmosphere absorbs less radiation). However, the dominant effect appears to be, by far, the change in the turbulent fluxes. Here, the albedo perturbations all entail (relatively) large decreases in the latent and sensible heat fluxes leading to a reduction in the counterradiation which is so pronounced that it constitutes the largest shift in any of the energy fluxes at the surface and causes an overall decrease in the surface net lw radiation, despite the cooling-induced reduction in surface emissions. In contrast, the increases in water availability only have a minor- or a positive impact on the turbulent fluxes and consequently on the counterradiation. And, as a result, all of the hydrological perturbations entail an increase in the surface net lw radiation, with the effect being most pronounced for the perturbation in the region between 15°N-35°N. This, strong dependency of the counterradiation on the turbulent fluxes is mainly related to the altitudes at which the latter influence the atmospheric state. Changes in the turbulent fluxes warm or cool the atmosphere particularly at low altitudes – in case of the latent heat flux via the condensational heat release – resulting in large changes to the re-emissions towards the surface. In contrast, temperatures close to the top of the atmosphere are much less affected, resulting in only minor changes to the TOA outgoing lw radiation. Thus, changes in the turbulent fluxes entail a lapse-rate feedback (e.g. in case of the hydrological perturbation between 15°N-35°N, indicated by the non-radiative contribution to the counterradiation being larger than the change in the turbulent fluxes), however, its effects are small relative to change in surface net sw radiation.

In case of the albedo perturbation between 10°S-10°N, the globally averaged decrease in net lw radiation is so strong that it far exceeds the initial reduction in sw radiation. This is possible due to a secondary, a hydrological, perturbation in the Amazon- and Congo basins. Here, the brightness increase initially cools the surface and the atmosphere and impedes the turbulent fluxes to a point at which convective processes are strongly inhibited, severely limiting precipitation. The resulting soil moisture deficit leads to a reduction in evapotranspiration which – though in the opposite direction – is almost as large as the increase in evapotranspiration in the simulation with an artificially increased water availability (Fig. SF1). The corresponding reduction in the latent heat fluxes, in turn, has a positive effect on temperatures, increasing both the sensible heat flux and the surface lw emissions. Here, the effects of the albedo increase and the subsequent decline in water availability do not fully offset each other, because the former reduces the lw emissions, the sensible heat flux as well as the latent heat flux, while the latter only increases the lw emissions and the sensible heat flux. Instead, the superimposition of the two perturbations entails a basin-wide constellation with higher surface temperatures but an overall colder and dryer atmosphere in combination with less atmospheric counterradiation and precipitation.

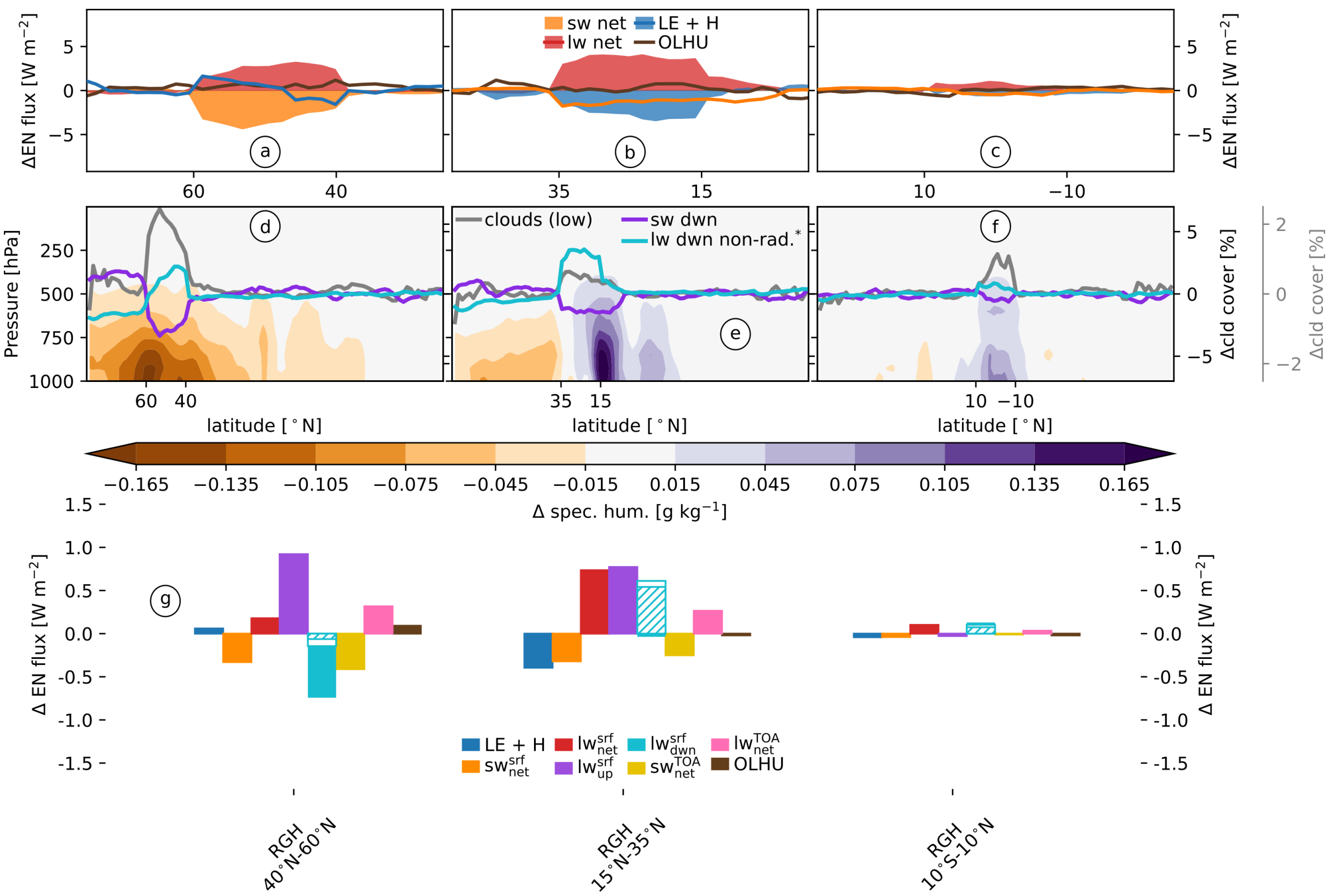

Figure SF6: **Changes in surface roughness**

Panels a-f show the same as Fig. 1 in the main manuscript – but for increases in surface roughness – panel g the same as Fig. SF5, with all y-axis ranges reduced by a factor of ≈ 3. The roughness perturbation was simulated by doubling the dynamically determined surface roughness length, with a minimum increase of 2 m.

Changes in surface roughness affect the land-atmosphere interactions in a way that is quite similar to changes in surface hydrology, since the dominant effect is also a shift in the surface moisture flux. However, the overall impacts are substantially smaller, for a number of reasons. On one hand, the changes in moisture flux are constrained by the availability of water and even large in- or decreases in boundary layer turbulence may entail only minor shifts in the moisture fluxes in (semi-) arid regions. On the other hand, roughness changes affect the turbulent mixing of sensible heat in the same way they affect the surface moisture fluxes, which diminishes the overall effect also in predominantly energy-limited regions: With a positive effect on the surface sensible heat flux, increases in surface roughness cool the atmosphere to a much lesser extent than increases in water availability. And the resulting increase in relative humidity, hence, in the rate of cloud formation is, therefore, much weaker, resulting in a much smaller effect on the planetary albedo. Furthermore, while an increased roughness, necessarily intensifies the boundary layer turbulence for given horizontal wind speeds, there is a very strong negative feedback, since the roughness increase also reduces the velocity of the winds that are generating the shear-driven turbulence. Here, the idealized increases in roughness considered in the present study reduce the horizontal, near-surface wind speeds in the perturbed regions by on average around 40% - 60%.

Roughness increases in the regions between 40°N-60°N still result in notably higher evapotranspiration rates and overall cooler, more saturated atmosphere, which notably increase the low-altitude cloud over. The latter reduces the surface solar irradiance, hence, the available energy, to the extent that the turbulent fluxes decrease, despite the enhanced turbulent mixing. The overall cooling reduces the total water content of the atmosphere – hence, the absorption of sw radiation. Thus, roughness changes in this region have a general effect on the surface energy balance and on the state of the atmosphere that is very consistent with the effects due to an increase in water availability – although substantially smaller. There are however, some differences related to the way the perturbations were implemented in the model. One key aspect is that the roughness modifications act and affect the cloud cover also during boreal winter, when the solar irradiance is small. Thus the roughness perturbation causes a smaller decline in (annual mean) surface solar irradiance relative to the increase in (annual mean) cloud cover than the hydrological perturbation.

The roughness perturbation in the region 15°N-35°N, still has some similarity to its hydrological counterpart. Here the key difference is that both sensible and latent heat flux increase in response to the higher surface roughness. Consequently, the corresponding increase in the turbulent fluxes is the main factor balancing the shift in the net lw radiation – as opposed to the surface net sw radiation – although it does not necessary change the total available energy since it is balanced by the increase in counterradiation. Furthermore, the combined increase of sensible and latent heat flux actually results in warmer and moister atmosphere within and south of the perturbed region. This leads to a higher sw absorption in the atmosphere which leads to a comparatively strong decrease in surface net sw radiation, despite the comparatively small reduction in the low altitude cloud cover.

For roughness changes in the region 10°S-10°N, the overall effects are so small, that they make it impossible to draw any robust conclusion.

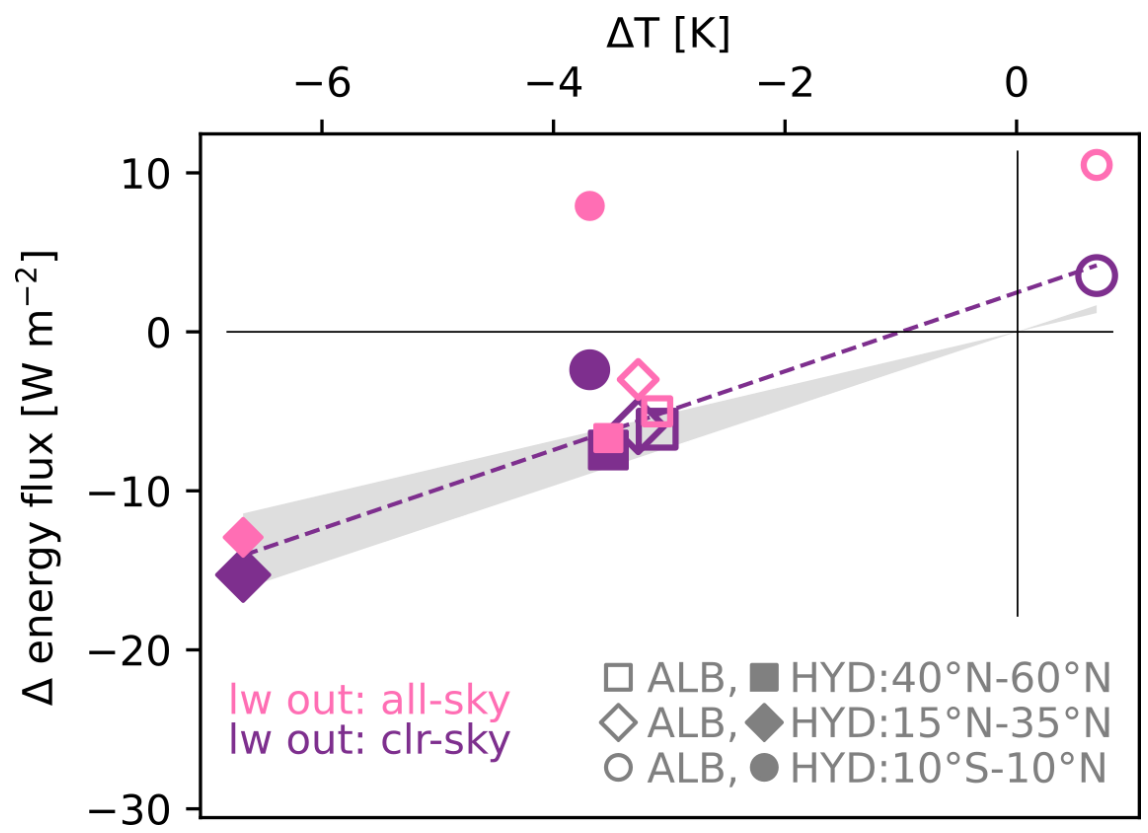

Figure SF7: **Local top-of-atmosphere-radiation-surface-temperature relations**

Shown are the same relationships as in panel a of Fig. 2 in the main manuscript but for surface temperatures and energy fluxes in the region of the initial perturbation and with the displayed variables being limited to the top-of-atmosphere (TOA) outgoing longwave (lw) radiatition.

On the global scale, changes in surface temperature and TOA radiative fluxes match the observations-, reanalysis- and model-based estimates of the slope in the lw-TOARSTR comparatively well (Fig. 2a). This, however, is not the case for the relationships within the region of the initial perturbation, even though there is still a statistically significant linear relation between the changes in surface temperature and clear-sky TOA outgoing lw radiation for the different perturbations. However, the intercept of this relationship strongly diverges from zero and the lw-TOARSTRs range between less than 1 $\mathrm{W\,m^{-2}\,K^{-1}}$ and more than 5 $\mathrm{W\,m^{-2}\,K^{-1}}$. For the all-sky lw radiation, this range is roughly between -2 $\mathrm{W\,m^{-2}\,K^{-1}}$ and 15 $\mathrm{W\,m^{-2}\,K^{-1}}$ and there is no significant agreement between the lw-TOARSTRs in the different experiments.

In particular, the stark contrast between the effects of hydrological perturbations in different regions strongly suggests that local temperatures are to a large extent determined by non-radiative effects, which is in line with the findings of previous studies [5, 31, 11]. The decrease in clear-sky outgoing lw radiation is about 4 times larger in the region 40°N-60°N than in the region 10°S-10°N for very similar decreases in surface temperature – that is -3.7 K and -3.6 K, respectively. Here, it is the tropical increase in water availability that most strongly deviates from the (quasi-) observation-based slopes in the clear-sky lw-TOARSTRs. In the two northern-hemisphere regions, the latent cooling of the surface is partly offset by an increase in atmospheric counterradiation but this is not the case in the tropics (Fig1. j-l). The reason for this appears to be that the drying of the atmosphere in the tropics occurs at greater heights - that is mainly above 750 hPa - which is likely connected to an inhibition of deep convection. As a result, less of the surface lw emissions are absorbed and re-emitted higher up in the atmosphere, leading to less counterradiation and more TOA outgoing lw radiation. Thus, the lapse rate effect leading to the pronounced shift in the lw-TOARSTRs is the combined result of the non-radiative cooling of the surface and a reduced greenhouse effect of water vapor. For the all-sky outgoing lw radiation, the respective changes are even of opposing signs, that is a decrease of about -7 $\mathrm{W\,m^{-2}}$ in the region 40°N-60°N and an increase of about 7 $\mathrm{W\,m^{-2}}$ in the region 10°S-10°N. Here, it is again the perturbation in the tropics that exhibits the largest deviation from the expected relationships, showing an increase in TOA outgoing lw radiation despite a cooling of the surface. This is similarly connect to the drying of the atmosphere – particularly above 750 hPa – which results in a marked reduction in cloud cover at heights between 400 hPa and 100 hPa, corresponding to a substantial decrease in the amount of heat that is trapped by high-altitude clouds.

There are also pronounced differences in the lw-TOARSTRs for the albedo changes, with the perturbation in the region 10°S-10°N exhibiting a ratio of about 5 $\mathrm{W\,m^{-2}\,K^{-1}}$ for the clear-sky fluxes- and 15 $\mathrm{W\,m^{-2}\,K^{-1}}$ for the all-sky fluxes. This does, however, not necessarily mean that brightness changes lead to non-radiative effects directly. Here, the albedo change caused a substantial decline in precipitation and, consequently, in evapotranspiration, making it plausible that the deviations of the lw-TOARSTRs from the expected range are caused by this secondary, hydrological perturbation. However, an in depth analysis of this feature is beyond the scope of the present study.

The fact that the local TOARSTRs are sensitive to shifts in surface hydrology but the global relations are not implies that non-radiative effects also necessarily modify the TOARSTRs in remote regions. This can happen in two ways: Either remote surface temperatures change, without a shift in the TOA outgoing lw fluxes, or the outgoing lw fluxes in remote regions change, without a notable surface temperature change. The answer to the question which of these two mechanisms is the dominant one may have important implications for the overall response since the latter also depends on the spatial temperature distribution [9, 2]. Here, non-local temperature effects from non-radiative processes could potentially trigger radiative feedbacks, connected to the temperature-dependency of the surface albedo and cloudiness: A non-radiative cooling in the tropics, for example, which results in a compensating surface warming in the boreal and polar regions, would induce an additional radiative heating via the albedo reduction from a loss in snow- and sea-ice cover. Thus non-radiative processes potentially affect the global temperature response by modifying the climate feedback parameter through changes in the spatial temperature patterns. Studies have primarily targeted the pattern effects connected to changes in sea-surface temperature[3], but there is some evidence suggesting a significant negative contribution to the global internal feedback parameter from changes in land temperatures[27]. Based on our experimental setup we can, unfortunately, not robustly assess which of the two mechanisms is the dominant one, mainly because the remote changes in surface temperature and TOA outgoing lw fluxes are primarily determined by the changes in lateral heat transport connected to the radiative forcing of the perturbations. Future studies could, however, address this question by prescribing similar perturbations of the land surface in so called cloud radiation denial experiments. These allow for a clear separation between effects of (cloud) radiative- and non-radiative processes, by artificially inhibiting some or all cloud radiative effects on climate [13].

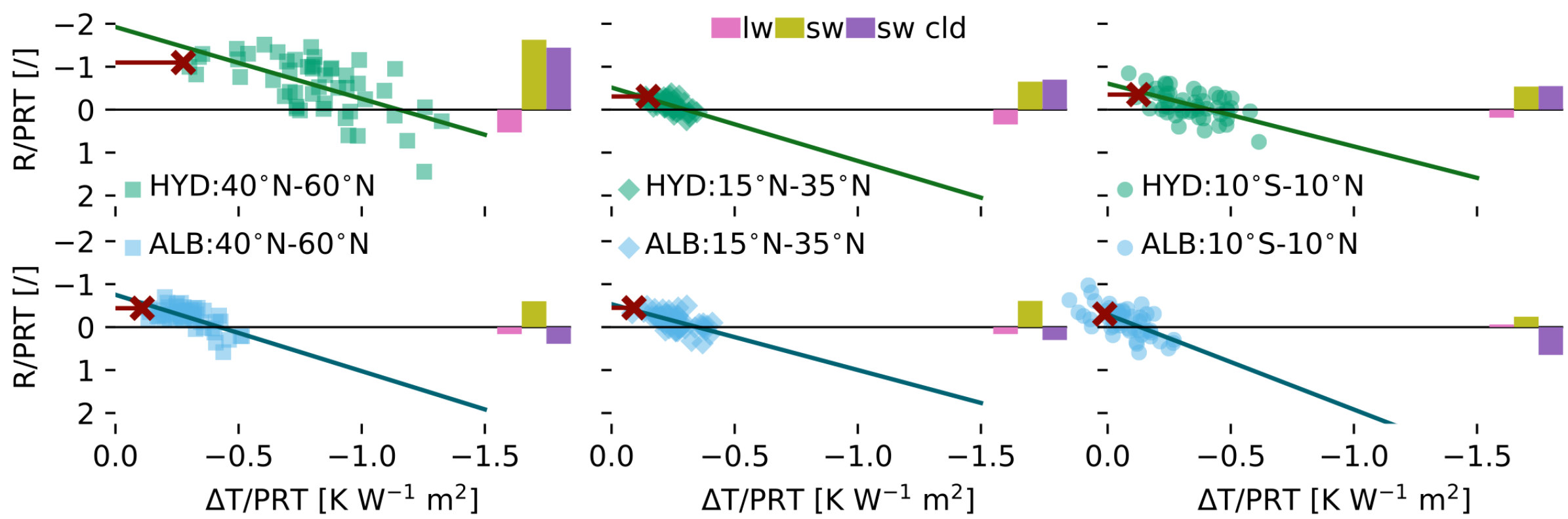

Figure SF8: **Radiative forcing and cloud sensitivities**

Shown is the annual mean top-of-atmosphere radiative imbalance (R) relative to the global mean temperature change (ΔT) – in fully coupled simulations – resulting from increases in water availability (HYD, green) and surface albedo (ALB, blue) in the latitudes 40°N-60°N, 15°N-35°N and 10°S-10°N, respectively, with lines giving the best linear fit. Red crosses and lines show the 50-year-mean TOA radiative imbalance in corresponding AMIP-type simulations (with prescribed sea-surface temperatures and sea-ice cover). At the right side of each panel, the latter is disaggregated into the long-wave (lw, pink) and short-wave contribution (sw, light green) and further into the short-wave cloud effect (sw cld, purple), as the difference between all-sky and clear-sky sw radiation. To facilitate the intercomparison between perturbations, the relationships are normalized based on the strength of the initial perturbation (PRT). In case of hydrological changes, the initial perturbation is given by the change in latent heat flux within the perturbed region (weighted by the area of the perturbed region relative to Earth's total surface area), and in case of albedo perturbations by the change in net sw radiation at the surface. Here, we didn't use the actually simulated change in net sw radiation, but to preclude the effects from differences in the cloud cover between the regions, we used a potential perturbation strength based on the prescribed brightness change in combination with the clear-sky surface solar irradiance in the unperturbed reference simulation. Please note that this deviates from the perturbation definition used in Fig. 1 of the main manuscript, where the strength of the albedo perturbation was quantified by the brightness change in combination with the all-sky surface solar irradiance in the unperturbed reference simulation.

It is clear that, in some of the fully coupled simulations, the inter-annual variability in the radiative imbalances is comparatively large while the overall effects are small and, without an ensemble of simulations, the Gregory method [10, 8] provides only a rough approximation of the radiative forcing exerted by land-surface change (indicated by the line's y-value at the origin on the x-axis). Likewise, the Hansen method [12] can only provide coarse estimates of the radiative forcing, since land-surface temperatures change in AMIP-type experiments allowing for some adjustment in the TOA outgoing long-wave radiation [1, 22, 30]. However, a qualitative assessment of the forcing for different perturbations is still informative, especially the comparison between perturbations in different latitudinal bands.

The disaggregation of the TOA radiative imbalance in the AMIP-type simulations clearly shows that the hydrological perturbations mainly act via cloud radiative effects. Here, the strength of the cloud response differs markedly between different latitudinal zones. For the perturbation in the region 40°N-60°N, the radiative forcing relative to the causative change in the latent heat flux is about 3-4 (2-3) times as large as for the region 15°N-35°N (10°S-10°N), indicating a substantially higher cloud sensitivity in the former. This is also the only region in which the radiative forcing is larger than the initial perturbation, with the additional clouds reflecting between 10 % and 66 % more energy back to space than was "required" to generate them in the first place. In contrast, a 1-$\mathrm{W\,m^{-2}}$-change in the latent heat flux only entails a -0.3 $\mathrm{W\,m^{-2}}$ to -0.5 $\mathrm{W\,m^{-2}}$ (-0.4 $\mathrm{W\,m^{-2}}$ to -0.9 $\mathrm{W\,m^{-2}}$) radiative forcing for the perturbation in the region 15°N-35°N (10°S-10°N). Interestingly, the albedo perturbation in the region 40°N-60°N also entails the largest radiative forcing – relative to the potential perturbation strength – despite the fact that the cloud-free surface fraction is substantially smaller than in the region 15°N-35°N. Here, the high ratio of radiative forcing and potential perturbation strength is only possible because the difference between cloud- and surface albedo is larger than the prescribed change in surface reflectivity so that changes in the cloud cover have the potential to induce changes in the TOA sw radiative fluxes that exceed the ones driven by the change in surface albedo (even in the complete absence of clouds). Thus, the radiative forcing is not only determined by the change in surface albedo itself but, in large parts, also by the cloud response to the resultant, cooling-induced increase in relative humidity, with the cloud sensitivity being highest in the region 40°N-60°N.

Overall, the above results strongly suggest that changes in the surface properties in mid- and high latitudes have a substantially larger biophysical effect on global climate than comparable changes within the tropics and the subtropics. Thus, they confirm the latitudinal dependency of the climate impact of land-surface changes, demonstrated in numerous studies [14, 15], highlighting the important role of the cloud cover characteristics in shaping this dependency [17, 16, 18, 20].

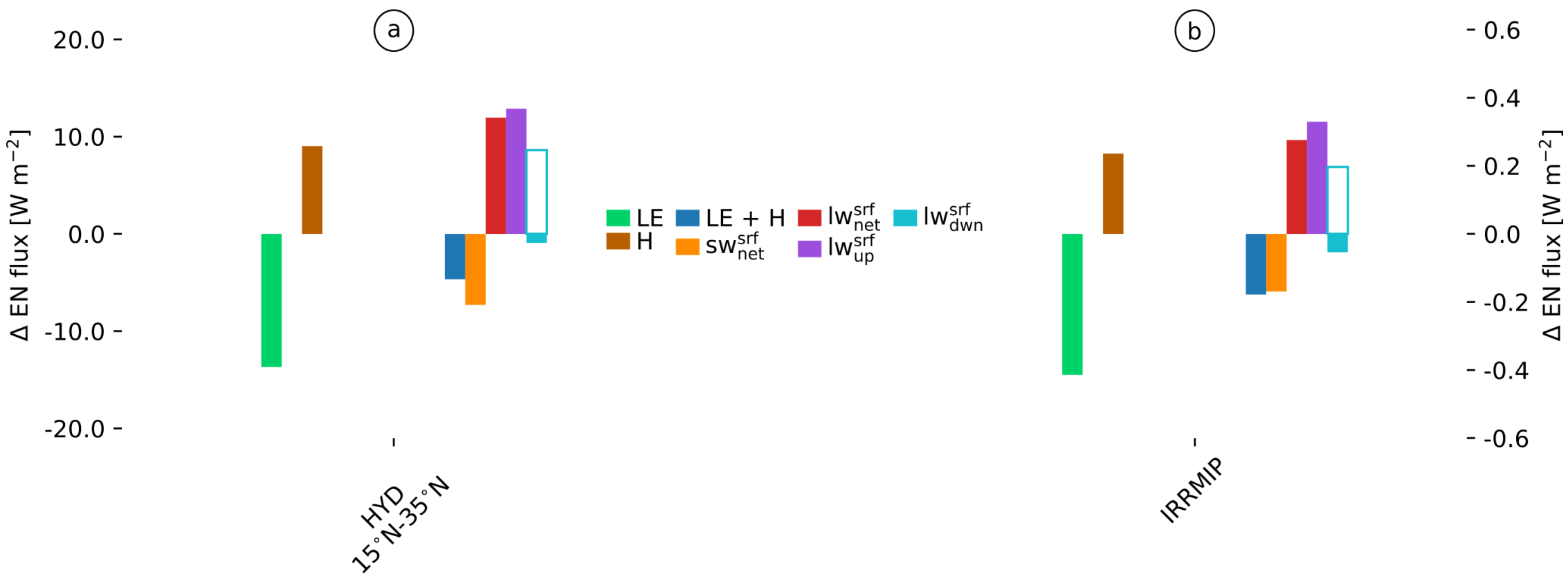

Figure SF9: **Effects of 20th-century irrigation expansion**

Shown are the changes in latent- (LE, green) and sensible heat flux (H, purple), the sum of the turbulent surface fluxes (LE + H, blue), the short-wave (sw_{net}^{srf}, orange) and long-wave (lw_{net}^{srf}, red) net radiation at the surface, the surface long-wave emissions (lw_{up}^{srf}, cyan) and the counterradiation (lw_{dwn}^{srf}, grey) resulting from increases in water availability, averaged over the global land area. White bars with grey outlines additionally indicate the change in counterradiation originating from changes in the turbulent fluxes, in the sw absorption in the atmosphere and in atmospheric heat exchange between ocean- and land grid cells. Panel a (left y-axis) shows the results for the idealized increase in water availability in the region between 15°N-35°N as simulated by MPI-ESM for a 50-year period under pre-industrial greenhouse gas concentrations. Panel b (right y-axis) shows the effects due to the historical expansion of irrigated areas during the period 1901-2014 as simulated by the IRRMIP participants (see below), with values showing the ensemble mean during the period 1985-2014. Positive fluxes indicate an increase in the energy content of the surface.

Agricultural irrigation arguably constitutes the most important anthropogenic intervention in the terrestrial hydrological cycle. Estimates of irrigation water withdrawals range between roughly 2000 and 3000 $km^3 yr^{-1}$, corresponding to 60 - 70 % of the withdrawals and 80 % of the consumption of freshwater. Roughly 20 % of cultivated areas – that is about 3-4 Mio km^2 – are equipped for irrigation, producing about 40 % of our food [26, 21]. A recent initiative, the Irrigation Model Intercomparison Project (IRRMIP), brought together a number of Earth system models, to estimate the impacts of the expansion of irrigated areas during the 20th century – that is from roughly 1 Mio km^2 equipped for irrigation to 3 Mio km^2 – on water resources as well as on regional and global climate [28]. With the majority of irrigated areas being located in the latitudes between 10°N and 40°N, mainly in South-, East and South-East Asia [25], the results of the IRRMIP simulations should be comparable with our hydrological perturbation between 15°N-35°N. However, the IRRMIP protocol consists of AMIP-style climate experiments [6], meaning that sea-surface temperatures and sea-ice extent are prescribed based on observations. Consequently, a comparison of the global energy balance and surface temperature is not without problems. Nonetheless, a comparison of the surface energy balance over terrestrial areas can still allow a qualitative answer to the question if the response to changes in surface hydrology is fundamentally different between the idealized perturbations, investigated in the present study, and the more realistic scenario targeted by IRRMIP. It should be noted that we show the ensemble mean of 4 models participating in IRRMIP, while 2 were excluded (see below).

The impacts of the historical irrigation expansion are almost two orders of magnitude smaller than those resulting from the increase in water availability in our idealized setup (note the different y-axes). The initial increase in the latent heat flux amounts to roughly 0.4 $W\,m^{-2}$, averaged over the global land surface, with the corresponding value in the idealized simulation being 14 $W\,m^{-2}$. Overall, the land surface cools by about 0.04 K due to the additional irrigation, while the idealized perturbation results in a land-surface cooling of about 2.0 K. But despite the different orders of magnitude in the effects and the differences in the setups, the responses to the two hydrological changes are qualitatively very similar. The increases in latent heat flux is, in both cases, predominantly balanced by a decline in the sensible heat fluxes, with a sensible to latent heat ratio of -0.57 due to the irrigation expansion and -0.65 for our idealized experiment. In case of the irrigation expansion, the resulting change in the turbulent fluxes is actually slightly larger than the decrease in the net sw radiation, however, the change in the turbulent fluxes is in both cases overcompensated by the non-radiative contributions to the counterradiation and the (ocean-to-land) atmospheric heat advection (note that we could not exclude the effects due to changes in atmospheric sw absorption but, as indicated by Fig. SF5, these should be minor). In both cases the latter results in a extremely small ratio between the changes in surface downward- and upward lw radiation, that is roughly 0.07 for the idealized perturbation and 0.16 for the irrigation expansion. Most importantly, the ratio of the change in surface net sw radiation and surface cooling resulting from the irrigation expansion is consistent with the idealized perturbation experiments – that is a ratio of 3.5 $W\,m^{-2}\,K^{-1}$, while the idealized perturbation resulted in a ratio of 3.8 $W\,m^{-2}\,K^{-1}$. And while the inter-model variability is very high (see below Fig. SF11), the overall response, thus, strongly suggests that the large-scale climate effect due to the expansion of irrigated areas during the 20th century is driven by shifts in the planetary albedo rather than by the evaporative cooling of the surface, which is also in line with previous studies reporting the cooling effect of real-world, agricultural irrigation to be dominated by indirect effects [23, 24, 7].

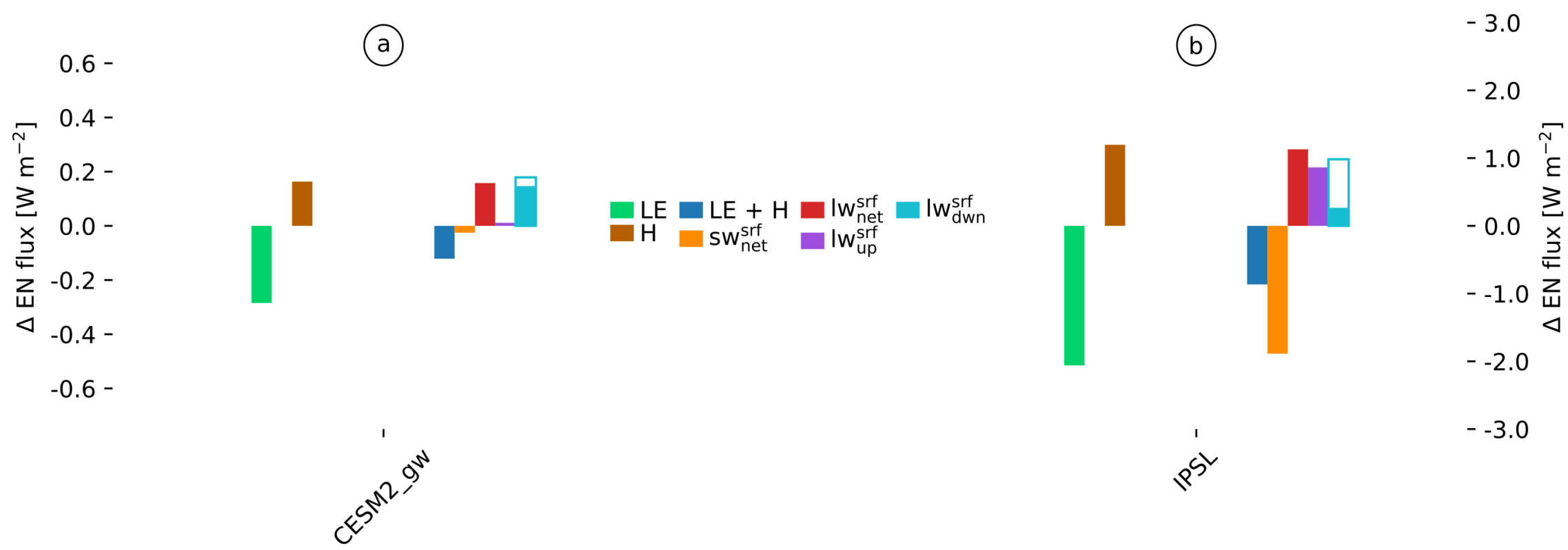

Figure SF10: **Models excluded from the IRRMIP analysis**

Same as SF9 but for CESM2 with groundwater withdrawal and flow representation (CESM_gw, panel a) and for IPSL (panel b).

From the 6 IRRMIP-participants for which sufficient output was available, we only included 4 models in our analysis, namely CESM2, CNRM, E3SM and NorESM, while the simulations from CESM2 with groundwater withdrawal (CESM_gw) and from IPSL were excluded from the analysis. In case of CESM_gw, the decision to exclude the model was based on the fact that the only difference to the standard CESM2 version is the inclusion of an intercell lateral ground-water interaction and an aquifer pumping process. And while the main features of the two models are identical, the CESM_gw simulations were repeatedly interrupted by model crashes – requiring a number of resets during the model run – and partly resulted in highly implausible energy fluxes. The reason not to include IPSL, on the other hand, was based on the fact that the simulations constitute extreme outliers from the ensemble, with the spatially integrated irrigation-induced changes in the energy fluxes being an order of magnitude larger than those simulated by the other models (note the different y-axes).

Yet despite their exclusion from the main analysis, the simulations are still consistent with the main findings of the present study. In case of CESM_gw, the expansion of irrigated areas mainly results in a notable increase in the turbulent fluxes, driven by a large increase in evapotranspiration. This however, does not result in an average cooling of the land-surface, with the terrestrial surface temperature actually increasing slightly. Here, the increase in the turbulent fluxes is completely offset by an increase in counterradiation, meaning that the local evaporative cooling is balanced by the (non-local) effects of the condensational heat release in the atmosphere and the lateral heat exchange. And without any notable effect on the incoming solar radiation there is no overall cooling. In contrast, the IPSL simulations show a notable cooling due to the irrigation expansion, however, here the model simulates a strong decrease in the net surface short-wave radiation – one that is roughly twice as large as the increase in the turbulent fluxes – in combination with an increase in counterradiation. This is consistent with our idealized increase in water availability between 15°N-35°N, strongly suggesting that in the IPSL simulations the cooling in the is also mainly driven by changes in the cloud radiative effect. However, it is also obvious that the increase in the turbulent fluxes and the decrease in the net shortwave radiation are very far from balancing the changes in the surface net long-wave fluxes. This implies that there is an extremely large change in the ground heat uptake which, in this magnitude, is not present in any other model.

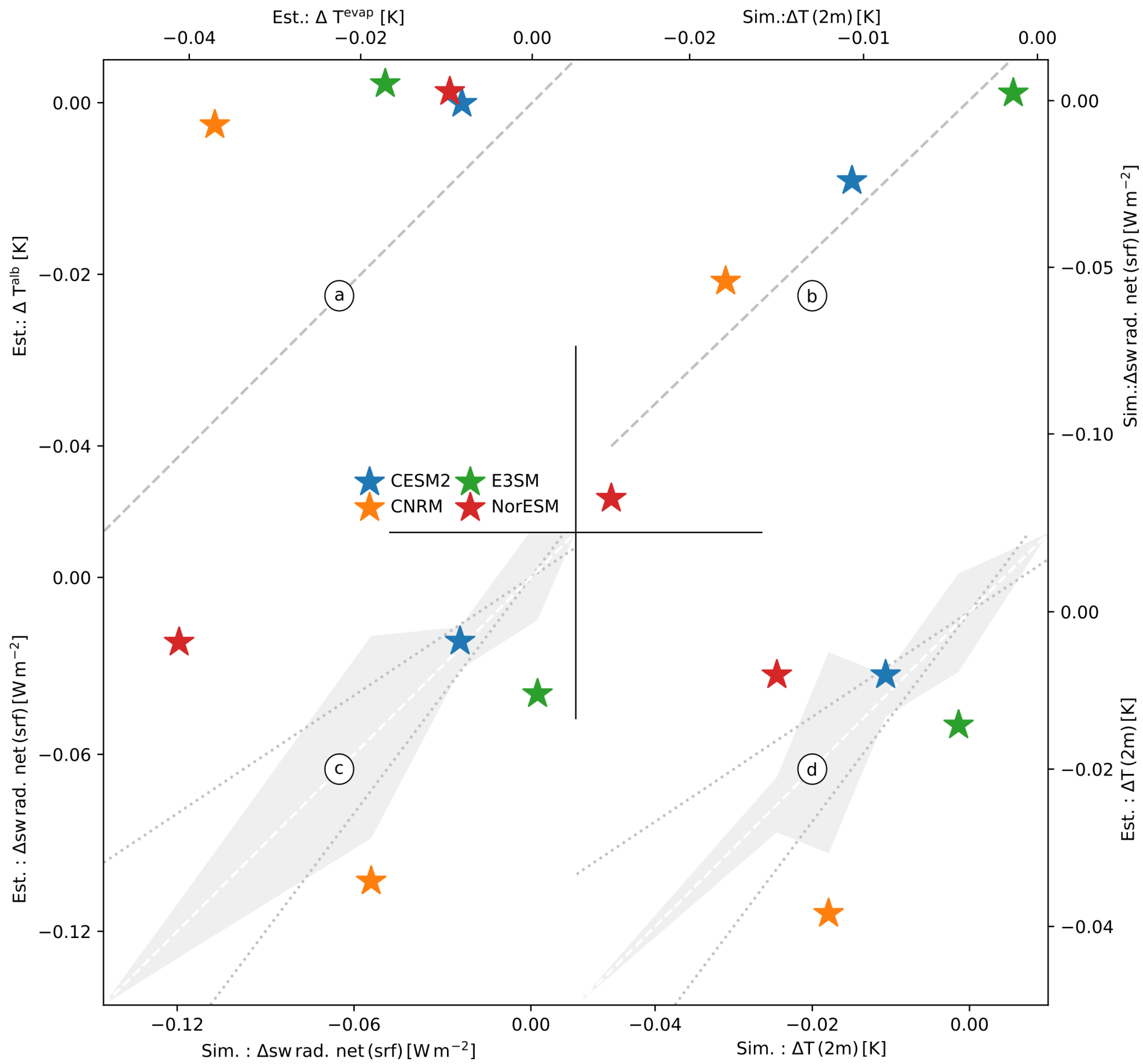

Figure SF11: **Global temperature response to 20th-century irrigation expansion**

Panel a shows the estimated contributions of effects from changes in evapotranspiration (ΔT^{evap}; x-axis) and surface brightness (ΔT^{alb}; y-axis) to the overall temperature change in response to the expansion of irrigated areas during the 20th century. Panel b shows the relation between simulated global-mean change in surface net short-wave radiation (Δ sw rad. net; y-axis) and surface temperature (ΔT (2m); x-axis). Panels c and d show the corresponding comparison between the simulated (x-axis) and estimated (y-axis) global mean response in surface net short-wave radiation (c) and 2-meter temperature (d). In panel b, the dashed gray line indicates the best linear fit and in panel a it gives the $y = x$ relation. In panel c and d, the white dashed line also indicates the $y = x$ relation, the gray dotted lines show the range of $y = x \pm \frac{|x|}{3}$ and the gray shading indicates the area $y = x \pm \frac{|y_{\text{alb}}|+|y_{\text{evp}}|}{3}$, where y_{alb} constitutes the estimated effects of changes in surface albedo and y_{evp} those due to changes in evapotranspiration. Since sea-surface temperatures are prescribed in the IRRMIP experiments, the simulated global mean values (panel b and x-values in panel c and d) are calculated assuming zero-values in ocean grid cells. The estimates (panel a and y-values in panels c and d) are based on the simulated changes in terrestrial evapotranspiration and surface albedo in the latitudes 10°N-40°N in combination with the sensitivity parameters determined for the idealized perturbations in the region 15°N-35°N. To be able to use the parameters for the AMIP-type simulations, they were scaled by the ratio of global mean temperature change assuming no changes in ocean cells and the global mean temperature change actually simulated for the idealized perturbation in the region 15°N-35°N. Note that the comparisons are being made for surface- instead of TOA net sw radiation due to limited data availability.

In all the simulations, the global temperature change in response to the the 20th-century expansion of irrigated areas is almost exclusively driven by changes in evapotranspiration (panel a). However, the IRRMIP participants produce very different global temperature changes, even though they agree that the overall effect is negligible – with the largest simulated response being a cooling of less than -0.03 K (panel b). CESM2, E3SM and NorESM actually simulate comparable changes in evapotranspiration (indicated by the similar estimates of the corresponding temperature effect [panel a]) which is not surprising since they have the same representation of irrigation and their land components are all based on versions of the Community Land Model (CLM5 in case of CESM2 and NorESM and CLM4.5 in case of E3SM). However, the largest disagreement on the simulated global temperature change actually exists between NorESM and E3SM (panel b). The differences in the temperature response appear to – very roughly – mirror those in the surface net sw fluxes (panels c and d) and there is a robust positive correlation between the two (panel b). Here, a perfect fit was not to be expected, especially considering that prescribing sea-surface temperatures can add or remove large amounts of energy at the ocean-atmosphere interface, which, in turn, affects the terrestrial energy balance by modifying the atmospheric heat and moisture exchange between ocean- and land areas. Nonetheless, the fact that the models which produce a larger (smaller) global cooling also exhibit a larger (smaller) decrease in surface sw radiation suggests that the main cause of the differences in the climate response is the cloud sensitivities in the models. In case of CESM2, E3SM and NorESM the large differences are somewhat surprising since the atmospheric components of the models are all based on versions of the Comunity Atmosphere Model (CAM6 in case of CESM2 and NorESM2 and CAM5 in case of E3SM), but it is highly consistent with a key result of the present investigation, namely that the climate impact of changes in the surface hydrology is mainly determined by the response of the low-altitude cloud cover. Here, the differences in the cloud sensitivity between models can also explain the poor agreement between simulated and estimated values, since the latter were determined based on sensitivity parameters obtained from MPI-ESM simulations. Here, there appears to be a comparatively good agreement between MPI-ESM and CESM2 and a particularly poor agreement between MPI-ESM and NorESM.

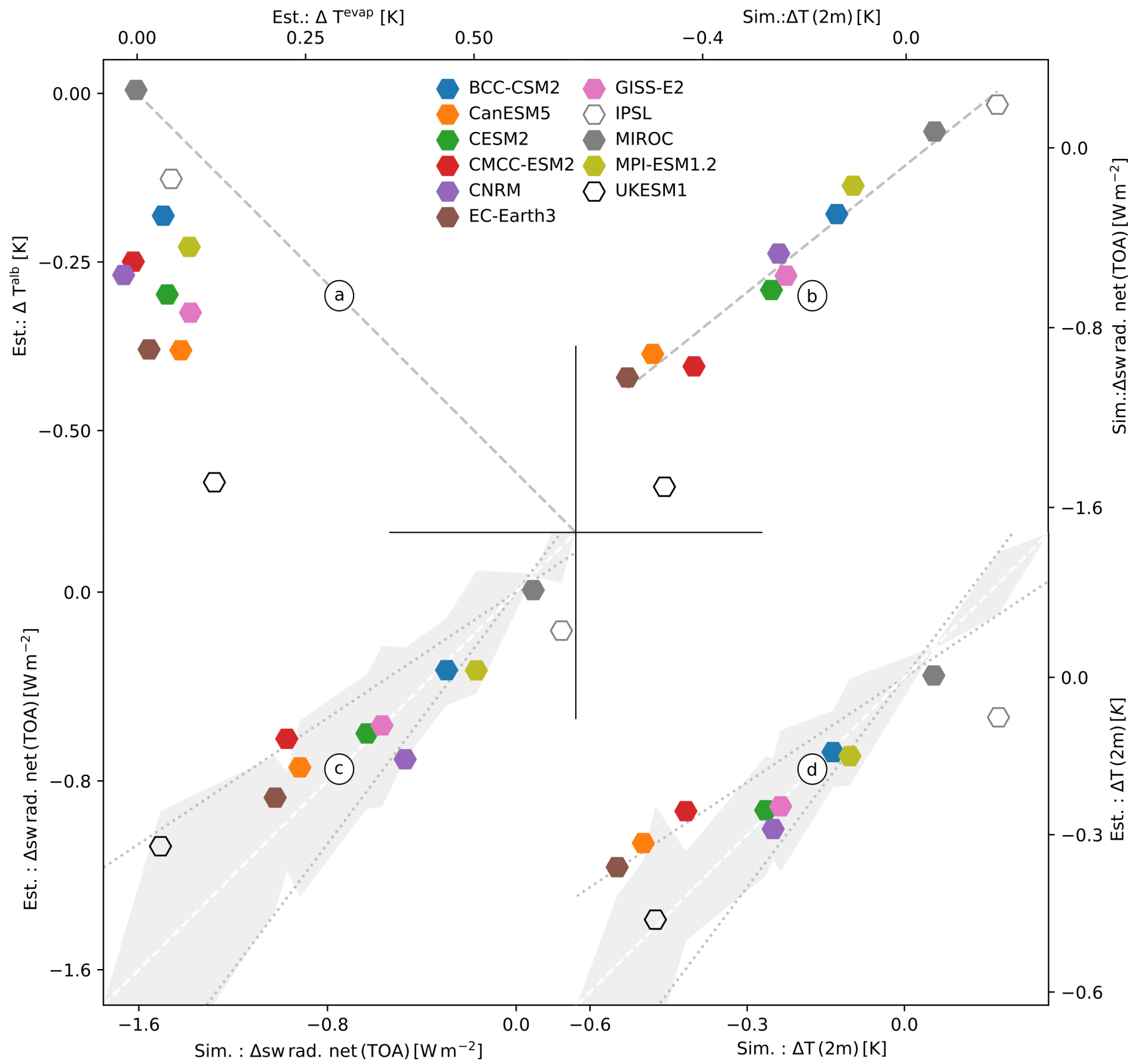

Figure SF12: **Global temperature response to idealized deforestation**

Shown is the same as in Fig. SF11 but for the idealized deforestation scenario (deforest-globe) simulated in the Land Use Model Intercomparison Project [19, 4, LUMIP]. For these simulations, 20 Mio. km^2 of the most densely forested areas were converted to natural grasslands, with data points showing the mean over a 30-year period following the conversion. The estimates of the global effects (panel a and y-values in panels c and d) are based on the simulated changes in terrestrial evapotranspiration and surface albedo in the latitudes 10°S-10°N, 10°S/N-35°S/N and 35°N-80°N in using the sensitivity parameters determined for the idealized perturbations in the region 10°S-10°N, 15°N-35°N and 40°N-60°N, respectively. Another difference to Fig. SF11 is that changes in top-of-atmosphere- instead of surface net sw radiation are being shown.

The IPCC's fifth assessment report [14, p. 688] and its special report on Climate And Land [15, p. 138,179] both highlight that the overall impact of land-surface change is not only determined by changes in the surface albedo and terrestrial CO_2 uptake, since non-radiative effects – mainly due to shifts in surface hydrology – modulate the temperature response notably. Here, the idealized deforestation experiment confirms this conclusion to the extent that the majority of models simulates a non-negligible contribution from changes in evapotranspiration to the overall temperature change (panel a). However, our results suggest that changes in surface hydrology entail only minor non-radiative impacts – since they mainly act via shifts in the planetary albedo – which is also supported by the deforest-globe simulations. Here, some models almost exclusively simulate a cooling due to brightness changes – that is purely radiative effects – (CEMCC-ESM2, CNRM and EC-Earth3), while other models additionally simulate non-negligible effects due changes in surface hydrology (IPSL, MPI-ESM, BCC-CSM2). Yet, these two groups of models produce very similar ratios between changes in TOA net sw radiation and in surface temperature (panel b). Here, the fact that this ratio is similar for almost all models – with the exception being UKESM1 (which was therefore not included in the calculation of the best linear fit in panel b) – is highly consistent with the key results of the present study, more specifically that changes in surface brightness and hydrology both predominantly affect the climate system via shifts in the planetary albedo, meaning that the combined temperature change is also proportional to the radiative effects.

Overall, the changes in TOA net sw radiation and surface temperature are also approximated reasonably well using the sensitivity parameters determined from the idealized MPI-ESM simulations (panels c and d), especially considering that, in many cases, the changes are the result of two large, opposing effects (a cooling due to an increase in surface brightness and a warming due to a decrease in evapotranspiration [panel a]). However, the LUMIP simulations also suggest that the MPI-ESM-based estimates of the sensitivities are most likely too low, with CNRM being the only model for which the overall impacts are overestimated (other than MPI-ESM itself). Here, MPI-ESM's planetary albedo appears to be comparatively insensitive to changes in surface albedo – e.g. indicated by the underestimated cooling for the CMCC-ESM2 simulation – as well as to changes in evapotranspiration – most prominently shown by the underestimated warming for the IPSL simulation (though here effects may not be without problems; see Fig. SF10). This strongly suggests that MPI-ESM overestimates the extent of the low-altitude cloud cover – hence, the low sensitivity to changes in surface brightness – but underestimates its sensitivity to land-surface change – hence, the low sensitivity to changes in evapotranspiration. This is also supported by the fact that MPI-ESM, has a low equilibrium climate sensitivity, in comparison to most CMIP6 models, mainly due to its low cloud sensitivity [29].

Supplementary Tables

Table ST1: Global mean differences in land-surface albedo[a] ($\Delta alb_{srf,lnd}$; [/]), irrigation[a] (irr; [mm d^{-1}]), terrestrial evapotranspiration[a] ($\Delta evap_{lnd}$; [mm d^{-1}]) and 2-meter temperature (ΔT (2m); [K]), between the perturbation experiments and an unperturbed reference simulation. Shown are the values for simulations with increased land-surface albedo (ALB) and increased water availability (HYD) in the latitudes between 40°N-60°N, 15°N-35°N and 10°S-10°N. Also included are the corresponding results for a decrease in surface albedo (ALB2) and a decrease in water availability (HYD2).

Experiment	$\Delta alb_{srf,lnd}$	irr	$\Delta evap_{lnd}$	ΔT (2m)
HYD 40°N-60°N	0.002	0.070	0.021	-0.65
ALB 40°N-60°N	0.011	0.0	-0.022	-0.60
HYD 15°N-35°N	0.000	0.140	0.136	-0.80
ALB 15°N-35°N	0.010	0.0	-0.026	-0.64
HYD 10°S-10°N	0.000	0.125	0.055	-0.46
ALB 10°S-10°N	0.006	0.0	-0.046	-0.11
HYD2 40°N-60°N	0.000	-0.016	-0.013	0.14
ALB2 40°N-60°N	-0.006	0.0	0.008	0.31
HYD2 15°N-35°N	0.000	-0.016	-0.016	0.06
ALB2 15°N-35°N	-0.007	0.0	0.028	0.49
HYD2 10°S-10°N	0.000	-0.027	-0.030	0.05
ALB2 10°S-10°N	-0.002	0.0	0.012	0.08

[a] Values do not represent the average over the continental surface but over the entire global area with 0-values in ocean cells.

Table ST2: Sensitivity parameters relating positive (↗) and negative (↘) shifts in evapotranspiration (evap.) and albedo (albedo) in the regions between 40°N-60°N, 15°N-35°N and 10°S-10°N, to the global mean change in toa sw net radiation (sw_{toa}), toa lw net radiation (lw_{toa}) and near-surface temperature (T (2m)). The sensitivity parameters relating evapotranspiration to the changes in toa radiation have the unit W m^{-2} mm^{-1} d and those relating evapotranspiration to surface temperature the unit K mm^{-1} d. With the surface albedo being unit-less, the corresponding units are W m^{-2} and K. Since the extent of land areas differs between the latitudes, the parameters are scaled by the ratio of land area within the latitudinal band of the perturbation and Earth's total surface area.

Sensitivities	sw_{toa}	lw_{toa}	T (2m)
↗ evap. (40°N-60°N)	-42.2	34.5	-15.6
↗ evap. (15°N-35°N)	-18.9	15.5	-7.0
↗ evap. (10°S-10°N)	-23.5	19.2	-8.7
↘ evap. (40°N-60°N)	-17.9	14.6	-6.2
↘ evap. (15°N-35°N)	-13.2	10.8	-4.6
↘ evap. (10°S-10°N)	-8.5	6.9	-2.9
↗ albedo (40°N-60°N)	-188.4	148.3	-77.1
↗ albedo (15°N-35°N)	-196.2	154.5	-80.3
↗ albedo (10°S-10°N)	-153.0	120.5	-62.6
↘ albedo (40°N-60°N)	-251.22	186.6	-109.7
↘ albedo (15°N-35°N)	-204.2	151.7	-89.2
↘ albedo (10°S-10°N)	-177.7	132.0	-77.6

Supplementary References

[1] Timothy Andrews, Christopher J. Smith, Gunnar Myhre, Piers M. Forster, Robin Chadwick, and Duncan Ackerley. Effective radiative forcing in a gcm with fixed surface temperatures. *Journal of Geophysical Research: Atmospheres*, 126(4), February 2021.

[2] Jonah Bloch-Johnson, Raymond T. Pierrehumbert, and Dorian S. Abbot. Feedback temperature dependence determines the risk of high warming. *Geophysical Research Letters*, 42(12):4973–4980, June 2015.

[3] Jonah Bloch-Johnson, Maria A. A. Rugenstein, Marc J. Alessi, Cristian Proistosescu, Ming Zhao, Bosong Zhang, Andrew I. L. Williams, Jonathan M. Gregory, Jason Cole, Yue Dong, Margaret L. Duffy, Sarah M. Kang, and Chen Zhou. The green's function model intercomparison project (gfmip) protocol. *Journal of Advances in Modeling Earth Systems*, 16(2), February 2024.

[4] Lena R. Boysen, Victor Brovkin, Julia Pongratz, David M. Lawrence, Peter Lawrence, Nicolas Vuichard, Philippe Peylin, Spencer Liddicoat, Tomohiro Hajima, Yanwu Zhang, Matthias Rocher, Christine Delire, Roland Séférian, Vivek K. Arora, Lars Nieradzik, Peter Anthoni, Wim Thiery, Marysa M. Laguë, Deborah Lawrence, and Min-Hui Lo. Global climate response to idealized deforestation in cmip6 models. *Biogeosciences*, 17(22):5615–5638, November 2020.

[5] Ryan M. Bright, Edouard Davin, Thomas O'Halloran, Julia Pongratz, Kaiguang Zhao, and Alessandro Cescatti. Local temperature response to land cover and management change driven by non-radiative processes. *Nature Climate Change*, 7(4):296–302, March 2017.

[6] W. Lawrence Gates, James S. Boyle, Curt Covey, Clyde G. Dease, Charles M. Doutriaux, Robert S. Drach, Michael Fiorino, Peter J. Gleckler, Justin J. Hnilo, Susan M. Marlais, T.Thomas J. Phillips, Gerald L. Potter, Benjamin D. Santer, Kenneth R. Sperber, Karl E. Taylor, and Dean N. Williams. An overview of the results of the atmospheric model intercomparison project (amip i). *Bull. Amer. Meteor. Soc.*, 80(1):29–55, Jan 1999.

[7] Aine M. Gormley-Gallagher, Sebastian Sterl, Annette L. Hirsch, Sonia I. Seneviratne, Edouard L. Davin, and Wim Thiery. Agricultural management effects on mean and extreme temperature trends. *Earth System Dynamics*, 13(1):419–438, February 2022.

[8] J. M. Gregory, T. Andrews, P. Ceppi, T. Mauritsen, and M. J. Webb. How accurately can the climate sensitivity to co_2 be estimated from historical climate change? *Climate Dynamics*, 54(1–2):129–157, October 2019.

[9] J. M. Gregory, T. Andrews, and P. Good. The inconstancy of the transient climate response parameter under increasing co2. *Philosophical Transactions of the Royal Society A: Mathematical, Physical and Engineering Sciences*, 373(2054):20140417, November 2015.

[10] J. M. Gregory, W. J. Ingram, M. A. Palmer, G. S. Jones, P. A. Stott, R. B. Thorpe, J. A. Lowe, T. C. Johns, and K. D. Williams. A new method for diagnosing radiative forcing and climate sensitivity. *Geophysical Research Letters*, 31(3), February 2004.

[11] Lorenz Hänchen, Benjamin Quesada, and Almut Arneth. Non-radiative effects dominate the local surface temperature response to land-cover change—insights from a semi-empirical model. *Journal of Environmental Management*, 380:124741, April 2025.

[12] J. Hansen, M. Sato, R. Ruedy, L. Nazarenko, A. Lacis, G. A. Schmidt, G. Russell, I. Aleinov, M. Bauer, S. Bauer, N. Bell, B. Cairns, V. Canuto, M. Chandler, Y. Cheng, A. Del Genio, G. Faluvegi, E. Fleming, A. Friend, T. Hall, C. Jackman, M. Kelley, N. Kiang, D. Koch, J. Lean, J. Lerner, K. Lo, S. Menon, R. Miller, P. Minnis, T. Novakov, V. Oinas, Ja. Perlwitz, Ju. Perlwitz, D. Rind, A. Romanou, D. Shindell, P. Stone, S. Sun, N. Tausnev, D. Thresher, B. Wielicki, T. Wong, M. Yao, and S. Zhang. Efficacy of climate forcings. *Journal of Geophysical Research: Atmospheres*, 110(D18), September 2005.

[13] Bryce E. Harrop, Jian Lu, L. Ruby Leung, William K. M. Lau, Kyu-Myong Kim, Brian Medeiros, Brian J. Soden, Gabriel A. Vecchi, Bosong Zhang, and Balwinder Singh. An overview of cloud–radiation denial experiments for the energy exascale earth system model version 1. *Geoscientific Model Development*, 17(8):3111–3135, April 2024.

[14] IPCC. *Climate Change 2013 - The Physical Science Basis: Working Group I Contribution to the Fifth Assessment Report of the Intergovernmental Panel on Climate Change*. Cambridge University Press, Cambridge, United Kingdom and New York, NY, USA, 2013.

[15] IPCC. *Climate Change and Land: IPCC Special Report on Climate Change, Desertification, Land Degradation, Sustainable Land Management, Food Security, and Greenhouse Gas Fluxes in Terrestrial Ecosystems*. Cambridge University Press, November 2022.

[16] Sarah M. Kang, Kiwoong Park, Fei-Fei Jin, and Malte F. Stuecker. Common warming pattern emerges irrespective of forcing location. *Journal of Advances in Modeling Earth Systems*, 9(6):2413–2424, October 2017.

[17] Sarah M. Kang and Shang-Ping Xie. Dependence of climate response on meridional structure of external thermal forcing. *Journal of Climate*, 27(14):5593–5600, July 2014.

[18] Marysa M. Laguë, Abigail L. S. Swann, and William R. Boos. Radiative feedbacks on land surface change and associated tropical precipitation shifts. *Journal of Climate*, page 1–63, May 2021.

[19] David M. Lawrence, George C. Hurtt, Almut Arneth, Victor Brovkin, Kate V. Calvin, Andrew D. Jones, Chris D. Jones, Peter J. Lawrence, Nathalie de Noblet-Ducoudrè, Julia Pongratz, and et al. The land use model intercomparison project (lumip) contribution to cmip6: rationale and experimental design. *Geosci. Model Dev.*, 9(9):2973–2998, Sep 2016.

[20] Hao Luo, Johannes Quaas, and Yong Han. Decreased cloud cover partially offsets the cooling effects of surface albedo change due to deforestation. *Nature Communications*, 15(1), August 2024.

[21] Sonali McDermid, Mallika Nocco, Patricia Lawston-Parker, Jessica Keune, Yadu Pokhrel, Meha Jain, Jonas Jägermeyr, Luca Brocca, Christian Massari, Andrew D. Jones, Pouya Vahmani, Wim Thiery, Yi Yao, Andrew Bell, Liang Chen, Wouter Dorigo, Naota Hanasaki, Scott Jasechko, Min-Hui Lo, Rezaul Mahmood, Vimal Mishra, Nathaniel D. Mueller, Dev Niyogi, Sam S. Rabin, Lindsey Sloat, Yoshihide Wada, Luca Zappa, Fei Chen, Benjamin I. Cook, Hyungjun Kim, Danica Lombardozzi, Jan Polcher, Dongryeol Ryu, Joe Santanello, Yusuke Satoh, Sonia Seneviratne, Deepti Singh, and Tokuta Yokohata. Irrigation in the earth system. *Nature Reviews EarthI& ; Environment*, 4(7):435–453, June 2023.

[22] Yi Qin, Mark D. Zelinka, and Stephen A. Klein. On the correspondence between atmosphere-only and coupled simulations for radiative feedbacks and forcing from co2. *Journal of Geophysical Research: Atmospheres*, 127(3), February 2022.

[23] William J Sacks, Benjamin I Cook, Nikolaus Buenning, Samuel Levis, and Joseph H Helkowski. Effects of global irrigation on the near-surface climate. *Clim. Dyn.*, 33(2-3):159–175, 2009.

[24] Steven C Sherwood, Vishal Dixit, and Chryséis Salomez. The global warming potential of near-surface emitted water vapour. *Environmental Research Letters*, 13(10):104006, September 2018.

[25] S. Siebert, P. Döll, J. Hoogeveen, J.-M. Faures, K. Frenken, and S. Feick. Development and validation of the global map of irrigation areas. *HESSD*, 2(4):1299–1327, August 2005.

[26] S Siebert, M Kummu, M Porkka, P Döll, N Ramankutty, and B R Scanlon. A global data set of the extent of irrigated land from 1900 to 2005. *HESS*, 19(3):1521–1545, 2015.

[27] David W. J. Thompson, Maria Rugenstein, Piers M. Forster, and Leif Fredericks. An observational estimate of the pattern effect on climate sensitivity: The importance of the eastern tropical pacific and land areas. *Journal of Climate*, 38(16):4233–4249, August 2025.

[28] Yi Yao, Agnès Ducharne, Benjamin I. Cook, Steven J. De Hertog, Kjetil Schanke Aas, Pedro F. Arboleda-Obando, Jonathan Buzan, Jeanne Colin, Maya Costantini, Bertrand Decharme, David M. Lawrence, Peter Lawrence, L. Ruby Leung, Min-Hui Lo, Narayanappa Devaraju, William R. Wieder, Ren-Jie Wu, Tian Zhou, Jonas Jägermeyr, Sonali McDermid, Yadu Pokhrel, Maxwell Elling, Naota Hanasaki, Paul Muñoz, Larissa S. Nazarenko, Kedar Otta, Yusuke Satoh, Tokuta Yokohata, Lei Jin, Xuhui Wang, Vimal Mishra, Subimal Ghosh, and Wim Thiery. Impacts of irrigation expansion on moist-heat stress based on irrmip results. *Nature Communications*, 16(1), January 2025.

[29] Mark D. Zelinka, Timothy A. Myers, Daniel T. McCoy, Stephen Po-Chedley, Peter M. Caldwell, Paulo Ceppi, Stephen A. Klein, and Karl E. Taylor. Causes of higher climate sensitivity in CMIP6 models. *Geophysical Research Letters*, 47(1):e2019GL085782, January 2020.

[30] Bosong Zhang, Brian J. Soden, Haozhe He, and Ryan J. Kramer. Decoupling land surface effects from co2 effective radiative forcing in a climate model. *Journal of Climate*, 39(1):65–76, January 2026.

[31] Guo Zhang, Huoqing Li, Jianduo Li, Chunlei Meng, and Xitian Cai. Non-radiative effect of land use/land cover differences on land surface temperature simulated by noah-mp land surface model over east asia. *Journal of Geophysical Research: Atmospheres*, 128(16), August 2023.
