# Supplementary material for: Global climate impact of land-surface change driven by radiative effects
Source: Nat Commun. 2026 Oct 3;17:10454. doi: 10.1038/s41467-026-77834-0 (PMC13634050; doi:10.1038/s41467-026-77834-0)
Supplement: Supplementary file 2 [file 41467_2026_77834_MOESM2_ESM.pdf]

nature portfolio

Peer Review File

Global climate impact of land-surface change driven by radiative effects

Corresponding Author: Dr Philipp de Vrese

This file contains all reviewer reports in order by version, followed by all author rebuttals in order by version.

Version 0:

Reviewer comments:

Reviewer #1

(Remarks to the Author)
Review of “Global climate impact of land-surface change driven by radiative effects” by Philipp de Vrese et al., submitted to Nature Communications
Overview
The authors use the coupled ocean–land–atmosphere model MPI-ESM to investigate how perturbations in surface albedo and water availability affect the surface energy balance and global/regional temperatures. The role of clouds in mediating these responses is emphasized throughout the manuscript. Overall, I find the study to be novel, and the analyses are generally informative and supportive of the main conclusions. However, in several places the logical connection between results and conclusions is not sufficiently developed. My detailed comments are provided below.
Major:
1. Given that MPI-ESM is an Earth System Model, I assume that the simulations employ dynamic vegetation rather than prescribed/static vegetation. If so, vegetation should respond to changes in surface albedo and water availability by experimental design. For example, altered photosynthesis and stomatal conductance could modify near-surface humidity, low-level cloud cover, and ultimately the radiation budget. Doutriaux-Boucher et al. (2009) demonstrate that CO_2-induced stomatal closure can rapidly reduce low cloud cover and increase radiative forcing, suggesting that such processes may be relevant here.
• Doutriaux-Boucher, M., Webb, M., Gregory, J. M., & Boucher, O. (2009). Carbon dioxide induced stomatal closure increases radiative forcing via a rapid reduction in low cloud. Geophysical Research Letters, 36, L02703. https://doi.org/10.1029/2008GL036273
Recent studies using climate models have shown that land-surface properties, including land surface temperature and soil moisture, can influence the effective radiative forcing of CO_2 (see below). This process is relevant to the present study and should be discussed.
• Andrews, T., Smith, C. J., Myhre, G., Forster, P. M., Chadwick, R., & Ackerley, D. (2021). Effective radiative forcing in a GCM with fixed surface temperatures. Journal of Geophysical Research: Atmospheres, 126(4), e2020JD033880.
• Zhang, B., Soden, B. J., He, H., & Kramer, R. J. (2026). Decoupling Land Surface Effects from CO 2 Effective Radiative Forcing in a Climate Model. Journal of Climate, 39(1), 65-76.
Back to MPI-ESM, is vegetation interactive or prescribed in these simulations? If vegetation is dynamic, how large are the vegetation changes, and how do they feedback onto the simulated climate response? If vegetation is static, what are the implications for the interpretation and generality of the results? Explicit discussion of this issue would substantially strengthen the manuscript.
2. Assumption of linearity between temperature response and planetary albedo around line 65:
I have difficulty following the reasoning that the temperature response to changes in surface properties can be approximated as a linear function of shifts in planetary albedo. Several studies suggest that the relationship between TOA radiative forcing and surface temperature response can be strongly non-linear and highly dependent on the spatial distribution of the forcing. In particular, radiative forcing applied at high latitudes often produces a larger global mean temperature response than the same forcing applied in the tropics, a concept closely related to forcing efficacy. Interestingly, the results in this manuscript appear to show similar behavior (e.g., Fig. 2), where surface albedo changes in mid- and high latitudes induce stronger cooling than comparable changes in the tropics.
Some references:
• Zhang, B., Zhao, M., He, H., Soden, B. J., Tan, Z., Xiang, B., & Wang, C. (2023). The dependence of climate sensitivity on the meridional distribution of radiative forcing. Geophysical research letters, 50(18), e2023GL105492.

• Kaur, H., Bala, G., & Seshadri, A. K. (2025). Why is the temperature response larger for radiative forcing imposed in high latitudes than for forcing imposed in low latitudes?. Climate Dynamics, 63(5), 1-21.
• Myhre, G., Byrom, R. E., Andrews, T., Forster, P. M., & Smith, C. J. (2024). Efficacy of climate forcings in transient CMIP6 simulations. Frontiers in Climate, 6, 1397358.
3. The land surface is analyzed using three latitudinal bands, but the land fraction differs substantially among these bands. In addition, the background mean state such as surface temperature and soil moisture (which directly relates to water availability) also varies strongly across the three bands. These differences complicate direct, “apples-to-apples” comparisons of climate impacts of land surface change since the amount of land surface involved is different.
The authors should clarify how these confounding factors affect the interpretation of the results, particularly with respect to global mean climate impacts. For example, to what extent do differences in land fraction or background climate state influence the apparent sensitivity in each band?
Minor:
• How is ocean and land heat uptake (OLHU) computed? Is it a direct model output, or is it derived from the energy budget, like the net surface energy budget? Some clarification on this would be helpful.
• Near line 85, the manuscript mentions opposite changes in latent heat (LE) and sensible heat (H). Why not show these two variables separately to better support this statement?
• Near line 95, is the reduced shortwave absorption attributed mainly to drying of the atmosphere boundary layer? How about the role of low clouds? I see a clear reduction in low cloud fraction in Fig 1 panel (i) for the tropical case.
• Section 2.2, again, the sensitivity of global-mean temperature to the location of the perturbation is reminiscent of previous studies (e.g., Zhang et al., 2023; Kaur et al., 2025). While the approaches are slightly different (surface albedo versus TOA solar radiation or solar constant perturbations), the results appear similar.
• Overall, the figures are well prepared, but some panels appear crowded. In Figure 1, for instance, several lines overlap, which reduces readability. It may help to divide the variables into additional panels or adjust the formatting to enhance clarity.

(Remarks on code availability)

Reviewer #2

(Remarks to the Author)
The main topic of the article is the impact of land surface changes driven by radiative effects on the global climate. The simulation was conducted using the Earth system model (MPI-ESM) of the Max-Planck Institute for Meteorology, and it was found that the influence of radiative forcing on the climate is more significant compared to non-radiative effects. This topic can help reorganize new understandings regarding climate mitigation measures, but it requires strengthening scientific reasoning and logical expression.
The article conducted extensive data analysis. However, there are many unclear expressions and scientific explanations in the full text and the attachments, making it difficult to support the credibility of the subsequent research analysis and results. The author has not yet clearly demonstrated which human activities have an impact on energy flux and have a feedback on the climate within these research scopes. Currently, there is no good argument or clear thinking.

Key comments:
The nature of the "land surface changes" mentioned in the article is unclear, which leads to the ambiguity of the conclusions drawn in the article.

The abstract reads like a description of facts or a conclusion, without any scientific data to back it up. It is difficult to assess the reliability of these descriptions.

Line 39: Using the shade of clothing colors to represent the effect of solar radiation is a bit too colloquial and needs to be revised and adjusted.

Line 45: “the impact of man-made alterations to the terrestrial hydrological cycle on global climate remains unclear to the extent that they constitute the only major anthropogenic driver whose contribution has never fully been quantified in the reports of the Intergovernmental Panel on Climate Change”. Carefully reading the content of the paragraph where this sentence appears, it is found that there are several unclear and ambiguous expressions. The sentence "the impact of man-made alterations to the terrestrial hydrological cycle on global climate remains unclear" - what does "man-made" refer to? Has there been no summary on this aspect in the IPCC report? Even the subsequent descriptions cannot effectively support the assumptions and designs in the text.

What exactly are the perturbations mentioned in the text referring to? Are there any clear and definite contents? How is it manifested? The text mentions "initial perturbation" and "perturbation regions" several times. How exactly are these quantified?

Discussion section: “Our results also help explain why present-day agricultural irrigation is of little consequence for the global mean temperature, despite large amounts of water being redistributed with profound local effects”. The author is recommended to conduct more in-depth verification to support the above statement. Existing related studies also suggest that irrigation in farmland has an impact on temperature. For instance, the cooling effect brought about by establishing new irrigation areas on uncultivated land is significantly greater than that in forest and grassland areas.

In the data availability section: It is necessary to provide a description of the data mentioned in the text. Simply mentioning that the data is stored in a certain location is not sufficient.

(Remarks on code availability)
By referring to, the guidelines for constructing climate models, the codes used for data processing, and the related explanations can all be viewed through the provided link.

Reviewer #3

(Remarks to the Author)
This manuscript investigates the extent to which changes in surface hydrology (primarily water limitation) and surface albedo influence global mean temperature through non-radiative effects. The authors conclude that such changes largely redistribute energy and primarily affect local temperatures, while the global mean temperature response remains governed by direct or indirect radiative effects arising from the initial perturbation.

I find this to be an important and interesting finding that fits well in the ongoing research about understanding how land-surface changes influence climate. I liked the experimental design. However, there are several areas where interpretation of results and clarity can be improved. In particular, some explanations of key physical mechanisms are difficult to follow, and certain sign conventions and energy balance formulations are not sufficiently described. Addressing these issues would be important for the journal's wider readership.

Specific comments:

Framing of line 95-100 needs to be clear. My understanding is that author's point is about linking increased albedo to surface cooling lowering the saturation vapor pressure that lead to lower atmospheric absorption of solar radiation and eventually leading to increase shortwave surface irradiance. So downward solar radiation increases but albedo effects are stronger and leads to reduce net SW and cooling? But these lines alone do not explain this reasoning well and may even seem counterintuitive to the reader. The authors may also think of having a conceptual figure to link different feedbacks described in the manuscript (also the one with changes in local humidity and counter radiation). These pathways are central to the manuscript and can be more clearly described.

Lines 86-87: The compensating role of sum of turbulent fluxes (H + LE) is very interesting but at the same time it's not so clear why sensible heat (H) reduces in response to increase evaporation (LE). Net radiation is not strictly constrained and depends on surface temperature (Ts). Therefore, H and LE could, in principle, vary independently through changes in Ts and necessarily need not compensate.

The findings of compensating effect of H and LE are however consistent with some recent works that shows total turbulent fluxes are constrained by radiation and that could explain the compensation (Conte et al., 2019, GRL; Ghausi et al., 2023, PNAS) and also some work on surface flux equilibrium (McColl et al., 2019 JAMES; McColl & Rigden, 2020 GRL).

Fig 1: It may be good to describe the sign conventions followed in estimating the changes. The surface energy balance can be written in different forms: Rs,net + Rl,net = H+LE+G or Rs,net = Rl,net + H + LE + G based on how the direction of net longwave flux is described. It may be good to mention the form of surface energy balance equation that is used and how the signs of changes /delta EN are estimated (atleast in the figure caption).

Fig 1 (a-c): For the albedo changes, while it is clear the reduction in shortwave is mostly balanced by associated decrease in H+LE. However Rl,net seems to have an opposite response in the perturbed region for tropics and mid-latitudes. Why is that?

Fig 1 (e): Turbulent fluxes (H+LE) have increased in midlatitudes (15 – 35 N) response to surface water changes but this pattern did not show up in any other region despite increase in counter radiation (Fig -1J). How does individual H and LE fluxes look here? Do they follow a compensation?

Fig 1 (i): Rs_down does not decrease much in the tropics despite similar increase in cloud-cover (%) in other regions. Is it partly compensated by decrease in specific humidity? Lw down also does not respond much to changes in humidity in the tropics.

Fig 2: Why does changes in hydrology and albedo has maximum effect on temperature when the changes are made in 15-35N? It will be good to have some discussion around it.

L173-176: It will be good to have a figure supporting it.

L278 – 280: This is a major assumption and shall be mentioned in the discussion section where limitations are listed.

(Remarks on code availability)

Version 1:

Reviewer comments:

Reviewer #1

(Remarks to the Author)
I would like to thank the authors for addressing my previous comments. I believe the manuscript is now in good shape and can be published after the editorial process.

(Remarks on code availability)

Reviewer #2

(Remarks to the Author)
How can the reliability of the obtained results be demonstrated in the text? There are no relevant considerations in the text.Are there any other reliable simulation models that present different results?

(Remarks on code availability)

Reviewer #3

(Remarks to the Author)
The authors have adequately addressed most of my previous comments and concerns.

For the comments related to the latitudinal patterns in Figure 1, I understand that the authors consider these questions to be beyond the scope of the present study and part of their ongoing research. However, I think it would be helpful to briefly mention this somewhere in the manuscript as a topic for future work. This would make it clear to the readers that these aspects are not yet fully understood.

With this minor suggestion, I believe that the manuscript is now suitable for publication.

(Remarks on code availability)

Open Access This Peer Review File is licensed under a Creative Commons Attribution 4.0 International License, which permits use, sharing, adaptation, distribution and reproduction in any medium or format, as long as you give appropriate credit to the original author(s) and the source, provide a link to the Creative Commons license, and indicate if changes were made.
In cases where reviewers are anonymous, credit should be given to 'Anonymous Referee' and the source.
The images or other third party material in this Peer Review File are included in the article's Creative Commons license, unless indicated otherwise in a credit line to the material. If material is not included in the article's Creative Commons license and your intended use is not permitted by statutory regulation or exceeds the permitted use, you will need to obtain permission directly from the copyright holder.
To view a copy of this license, visit https://creativecommons.org/licenses/by/4.0/

Replies to reviewer comments on:
"Global climate impact of land-surface change driven by radiative effects"

Dear Reviewers,

please find below our responses to your comments on our manuscript. Here, we repeat the comments in red letters, our response is given in black and passages that are included in the revised manuscript are given in blue.

Before starting the point-by-point address of your comments, we would like to sincerely thank all of you for the careful reading of our manuscript and for the insightful comments. We hope to have made the best use of them in the revision our manuscript. We would also like to highlight that, in response to the last comment of Reviewer#3, we tried to improve the way we balance the artificial water inputs in the simulations with hydrological perturbations. While the water balance violations remain in the order of the fluxes used in water hosing experiments, we managed to improve them quite a bit. Importantly, this also has an (small) effect on the results presented in the manuscript, though qualitatively they remain the same. The only instance in which the new simulations required a more than minor adjustment to the text was for the global comparisons of out top-of-atmosphere going longwave radiation and surface temperatures. Here, the new simulations showed an non-negligible radiative effect due to changes in high altitude cloud cover, which we incorporated in the revised manuscript as follows (see also Fig. RspF-3 at the end of the response letter):

The changes in spatially integrated TOA radiation, on the other hand, are highly consistent with the global temperature signal (Fig. 2 b). Here, observations- and reanalysis-based estimates place the slope in the clear-sky lw-TOARSTR at between 1.9 and 2.2 $\mathrm{W\,m^{-2}\,K^{-1}}$ and suggest similar relationships for all-sky conditions[1,2]. For the simulated response to changes in the surface properties, none of the clear-sky lw-TOARSTRs deviates from this range by more than 10%, and there is no systematic difference between perturbations in different latitudes or between albedo- and hydrological perturbations. For all-sky lw-TOARSTRs, only the hydrological perturbation in the tropics falls slightly outside this range, indicating that, here, radiative effects associated with shifts in high-altitude cloud cover notably modify the TOARSTRs.

Finally, it should also be noted that the color schemes in all figures were slightly adjusted to enhance visual clarity and accessibility.

Yours sincerely,
The Authors

Reviewer #1 (Remarks to the Author):

Major:

1. Given that MPI-ESM is an Earth System Model, I assume that the simulations employ dynamic vegetation rather than prescribed/static vegetation. If so, vegetation should respond to changes in surface albedo and water availability by experimental design. For example, altered photosynthesis and stomatal conductance could modify near-surface humidity, low-level cloud cover, and ultimately the radiation budget. Doutriaux-Boucher et al. (2009) demonstrate that CO_2-induced stomatal closure can rapidly reduce low cloud cover and increase radiative forcing, suggesting that such processes may be relevant here.

- Doutriaux-Boucher, M., Webb, M., Gregory, J. M., & Boucher, O. (2009). Carbon dioxide induced stomatal closure increases radiative forcing via a rapid reduction in low cloud. Geophysical Research Letters, 36, L02703. https://doi.org/10.1029/2008GL036273

Recent studies using climate models have shown that land-surface properties, including land surface temperature and soil moisture, can influence the effective radiative forcing of CO_2 (see below). This process is relevant to the present study and should be discussed.

- Andrews, T., Smith, C. J., Myhre, G., Forster, P. M., Chadwick, R., & Ackerley, D. (2021). Effective radiative forcing in a GCM with fixed surface temperatures. Journal of Geophysical Research: Atmospheres, 126(4), e2020JD033880.
- Zhang, B., Soden, B. J., He, H., & Kramer, R. J. (2026). Decoupling Land Surface Effects from CO 2 Effective Radiative Forcing in a Climate Model. Journal of Climate, 39(1), 65-76.

Back to MPI-ESM, is vegetation interactive or prescribed in these simulations? If vegetation is dynamic, how large are the vegetation changes, and how do they feedback onto the simulated climate response? If vegetation is static, what are the implications for the interpretation and generality of the results? Explicit discussion of this issue would substantially strengthen the manuscript.

Vegetation is indeed dynamic in the MPI-ESM simulations that we employ. Consequently, the model includes vegetation-related feedback effects with respect to (i) surface albedo, (ii) resistance to evapotranspiration, and (iii) surface roughness. The only feedback that is not represented is the change in atmospheric CO_2 that would result from vegetation changes, because greenhouse gas concentrations are prescribed in all of our experiments. In general, the behavior of MPI-ESM is largely consistent with the studies cited by the reviewer and, also with the work of Lague et al.[3,4], who used a very similar experimental set-up. Here, one of our main conclusions is that changes in water availability act mainly through radiative effects from changes in low-altitude cloud cover. This implies that, since vegetation cover and water availability are directly and in directly affected by CO_2, changes in these properties also modulate the effective radiative forcing of a given CO_2 perturbation, which agrees well with reduced transpiration increasing radiative forcing via a rapid reduction in low cloud (Boutriaux-Boucher, Gregory & Boucher (2009)). And though we discuss this specifically only in the supplementary material, we also found a latitudinal dependency of the radiative forcing resulting from change in the surface properties. E.g. in SF7 (now SF8) we discussed that "For the perturbation in the region 40N-60N, the radiative forcing relative to the causative change in the latent heat flux is about 3-4 (2-3) times as large as for the region 15N-35N (10S-10N).

The vegetation response is also considerable in some of the simulations. In particular, the substantially reduced evapotranspiration for the tropical albedo perturbation is in parts driven by a massive die-back of the rainforest (reductions in vegetation cover of up to 50%). It is, thus, one of the key reasons why the albedo experiment in the tropics exhibits such a small temperature response; it is essentially a superposition of two very large, opposing perturbations – an increase surface albedo and a reduction in cloud cover – whose overall temperature response is, importantly, still proportional to the overall change in the planetary albedo. However, these feedbacks are extremely complex and a detailed analysis would substantially enlarge the present manuscript. This was also one of the reasons why we choose to conduct our main analysis focusing on the overall changes in the planetary albedo – including the feedback effects. We did conduct complementary simulations with fixed SSTs to isolate forcing from feedback effects, however – as also indicated by Andrews at al. (2021) and Zhang et al. (2026) – this common experimental setup is problematic especially in the context of land-surface perturbations, because the respective surface

temperatures and therefore OLR can adjust to the perturbations. Therefore, we were very careful as to draw conclusions with respect to radiative forcing alone and provide the respective analysis only in the supplementary materials.

Nonetheless, we agree with the reviewer that the treatment of feedbacks, particularly those from vegetation shifts, should be be clarified in the manuscript. Strictly speaking, our analysis of TOA mean radiation and surface temperature may not require such a clarification, but the way feedback effects are accounted for is highly important for defining the strength of the initial perturbation and for estimating the cloud sensitivities. Thus, we now include the following clarifications in the methods section, with the first one following the initial decription of the simulation setup:

It should be noted that, in all experiments, the surface properties are partly dynamic because the model determines vegetation, snow, and sea-ice cover interactively. Consequently, the model includes vegetation-related feedbacks with respect to (i) surface albedo, (ii) resistance to evapotranspiration, and (iii) surface roughness. The only feedback that is not represented is the change in atmospheric CO_2 concentrations that would result from vegetation changes, because CO_2 is prescribed in all of our experiments. These feedback effects have important implications for how we define the initial perturbation in terms of energy, to make the perturbation strengths inter-comparable. For surface-albedo experiments we define the initial perturbation as the downwelling short-wave radiation in the unperturbed reference run, multiplied by the prescribed albedo offset. By construction, this definition excludes any albedo effects that arise from changes in vegetation or snow cover. For the water-availability experiments such a straightforward energy definition is problematic due to the way the perturbation is introduced in the model, that is by modifying the filling of the skin reservoir. Computing the perturbation as "added water x latent heat of vaporisation" would be misleading, since the extra water may be routed to runoff rather than to evaporation. Instead we define the initial perturbation as the difference in latent-heat flux between the perturbed- and the control simulation, evaluated over the perturbed region. In a strict sense, this definition necessarily includes all surface feedbacks – especially those from vegetation – because the moisture flux into the atmosphere is also controlled by leaf area and stomatal conductance. Importantly, however, the amount of water added to the skin reservoir is also determined interactively and responds to the shifts in atmospheric water demand related to changes in transpiration; therefore any vegetation-induced increase or decrease in moisture flux is automatically compensated by the reservoir forcing. In practice this makes the changes in vegetation cover largely irrelevant for the hydrological perturbation.

, and the second one following our description of how the sensitivity parameters were determined:

As stated above, the surface properties in all simulations are, in parts, dynamic because the model determines the vegetation-, snow- and sea-ice cover interactively. This makes it difficult to isolate the effects that stem from a given albedo or hydrological perturbation from those resulting from local and remote changes in the other surface properties. Here, we adapted an iterative approach in which we initially determined the cloud sensitivities in simulations with a reduced water availability, that entailed only negligible effects on the surface albedo. Based on these cloud sensitivities, we were able to factor out the respective effects in simulations with prescribed changes in surface albedo, which were then used to isolate the changes in cloud cover resulting purely from changes in the surface hydrology. A more detailed description of the approach is given in the Supplementary Material SF1, SF2.

2. Assumption of linearity between temperature response and planetary albedo around line 65: I have difficulty following the reasoning that the temperature response to changes in surface properties can be approximated as a linear function of shifts in planetary albedo. Several studies suggest that the relationship between TOA radiative forcing and surface temperature response can be strongly non-linear and highly dependent on the spatial distribution of the forcing.

In particular, radiative forcing applied at high latitudes often produces a larger global mean temperature response than the same forcing applied in the tropics, a concept closely related to forcing efficacy. Interestingly, the results in this manuscript appear to show similar behavior (e.g., Fig. 2), where surface albedo changes in mid- and high latitudes induce stronger cooling than comparable changes in the tropics. Some references:

- Zhang, B., Zhao, M., He, H., Soden, B. J., Tan, Z., Xiang, B., & Wang, C. (2023). The dependence of climate sensitivity on the meridional distribution of radiative forcing. Geophysical research letters, 50(18), e2023GL105492.
- Kaur, H., Bala, G., & Seshadri, A. K. (2025). Why is the temperature response larger for radiative forcing

imposed in high latitudes than for forcing imposed in low latitudes?. Climate Dynamics, 63(5), 1-21.

- Myhre, G., Byrom, R. E., Andrews, T., Forster, P. M., & Smith, C. J. (2024). Efficacy of climate forcings in transient CMIP6 simulations. Frontiers in Climate, 6, 1397358.

Here, we apologize if our formulations were somewhat misleading. The reviewer is absolutely correct that the same radiative forcing applied in different regions can result in different global mean temperature responses, because the feedback effects will differ. In fact our analysis, additionally shows, that comparable land surface perturbations (albedo, hydrology) will also entail a different radiative forcing depending on the latitude they are applied in. We suspect that the confusion may have arisen because the statement in line 65 ff is far more trivial than what we think was understood by the reviewer. Here, we do not specifically address the TOA radiative forcing but the overall effect on the planetary albode, that is the radiative forcing in combination with any feedback effects that modify the reflected shortwave radiation. The statement thus merely clarifies that: In equilibrium TOA outgoing longwave is equal to TOA shortwave absorbed and, therefore, as long as TOA outgoing longwave radiation features a linear relation ship with surface temperatures, so does TOA shortwave absorbed. Though trivial, we make this statement to clarify that the only way non-radiative effects could modulalte surface temperatures directly is by changing the TOA radiation surface temperature relationship. To clarify that we are talking about the combined effect on the planetary albedo we modified the paragraph in the following way:

The land-atmosphere exchange is shaped by highly complex, often non-linear dependencies. Nonetheless, the top-of-atmosphere (TOA) outgoing longwave radiation largely follows a linear relationship with the temperature of the underlying surface[1,2,5–7]. This suggests that changes in global-mean temperature relate to changes in Earth's total longwave emissions in a similarly linear manner. Furthermore, while the efficacy of a forcing – i.e., the temperature response per unit TOA radiative forcing – depends on where the perturbation is applied[8–10], the overall amount of short-wave radiation absorbed by a system in equilibrium must equal its longwave emissions. Therefore, the equilibrium change in net TOA shortwave radiation (forcing and feedbacks) follows the same linear relationship with surface temperature as the outgoing longwave radiation. This, in turn, implies that the spatially integrated, long-term temperature response to changes in Earth's physical surface properties is, regardless of type or location, simply a linear function of the resultant shift in planetary albedo. That is, unless non-radiative effects notably alter the TOA-radiation-surface-temperature relations [11, 12, TOARSTR].

3. The land surface is analyzed using three latitudinal bands, but the land fraction differs substantially among these bands. In addition, the background mean state such as surface temperature and soil moisture (which directly relates to water availability) also varies strongly across the three bands. These differences complicate direct, "apples-to-apples" comparisons of climate impacts of land surface change since the amount of land surface involved is different. The authors should clarify how these confounding factors affect the interpretation of the results, particularly with respect to global mean climate impacts. For example, to what extent do differences in land fraction or background climate state influence the apparent sensitivity in each band?

We thank the reviewer for pointing out that the implications of the large differences in the characteristics of the perturbed regions have not been sufficiently addressed. The albedo and skin-reservoir perturbations were imposed uniformly within each band and were not calibrated to produce identical global-mean forcings. And, because the bands contain very different extents of land and experience distinct temperature-and precipitation regimes, the same local perturbation inevitably results in (i) different volumes of water added to the system and (ii) different magnitudes of change in the (global-mean) surface albedo. Although one could in principle adjust the latitudinal widths so that each band encloses a comparable land area or restrict the perturbation to a subset of grid cells, such "patterned" or size-adjusted experiments could themselves modify the climate response by altering the spatial distribution of the forcing and the associated feedbacks. For this reason we adopted the simplest configuration – the same spatially uniform perturbation applied over the entire land surface in equal-width latitude bands. However, this means that a direct numerical comparison of the absolute temperature responses across the three simulations would be misleading. Instead, we focus on the qualitative differences in regional responses and the resulting spatial patterns, and when quantitative results are presented we examine the relationship between the integrated forcing (e.g., total perturbation strength or overall change in planetary albedo) and the global-mean temperature response. This approach removes the first-order bias introduced by the differing land-area fractions and background-climate states, allowing a robust comparison of the underlying physics across the three latitude bands. The following clarifications have been added to the methods section of the manuscript:

With respect to the initial perturbation it should also be noted that the experiments were not designed to produce comparable global-mean effects. The three latitudinal bands differ markedly in continental area and in their background-climate states. Consequently, applying the same albedo offset or the same target filling of the skin-reservoir inevitably yields (i) different amounts of water added to the system and (ii) different changes in the global-mean surface albedo. In principle one could (a) adjust the widths of the latitudinal bands so that each band contains a similar land area, (b) restrict the perturbation to a subset of land grid-cells within a band, or (c) adjust the albedo offset and target filling to compensate for the differences in land area and climate within the perturbed regions. However, such "patterned" or size-adjusted perturbations may themselves modify the climate response (e.g., by altering the spatial distribution of the forcing and the associated feedbacks). For this reason we chose the simplest configuration: the same spatially uniform perturbation applied over the entire land surface within equal-width latitudinal bands. We acknowledge that a direct comparison of the absolute temperature responses between the three simulations is therefore of limited meaning. Instead, our analysis emphasizes qualitative differences in the regional responses and the resulting spatial patterns. When we present quantitative results we focus on the proportionality between the spatially integrated driver (e.g., perturbation strength or total change in planetary albedo) and the climate response, thereby removing the first-order effects of differing land-area fractions and background-climate states and enabling a meaningful comparison of the underlying physics across the three latitude bands.

Minor:

- How is ocean and land heat uptake (OLHU) computed? Is it a direct model output, or is it derived from the energy budget, like the net surface energy budget? Some clarification on this would be helpful.

  The land and ocean heat uptake is calculated as the energy balance residual: $OHLU = SW_{srf,net} + LW_{srf,net} - LE - H$. Over land, these fluxes are not fully identical to the ground heat flux since MPI-ESM does not compute the latter at the surface, but between the first and second layer of the soil. The differences are, however, negligible on any temporal scale but at the time-step level. There is another issue concerning the general closure of the energy balance in MPI-ESM – which is particularly relevant for our setups – namely that energy is not strictly conserved since temperatures of river discharge are not explicitly simulated. As a result the ocean heat uptake calculated by the model is also not necessarily equal to the energy balance residual at the ocean surface. However, as these mismatches are generally accepted in ESM simulations, we had not previously addressed them specifically and only do so in the revised manuscript (see also the last comment of reviewer #3).

- Near line 85, the manuscript mentions opposite changes in latent heat (LE) and sensible heat (H). Why not show these two variables separately to better support this statement?

  The idea was to keep the figure as simple as possible, with the key information being the two radiative fluxes (LW/SW) and the non-radiative term as the sum of LE, H and OHLU. In the end we also decided to treat OHLU separate from the turbulent fluxes, since the former does provide an overall impression of the effects of the simulations not being in full equilibrium. However, further separating LE from H does diminish the visual readability of the figure substantially because the absolute values of both fluxes taken separately are so large that the details of most of the other components are very difficult to see when being plotted on the same y-axis. However, we acknowledge that the direct comparison of LE and H would help show that these are in fact two large opposing fluxes and we included an additional figure in the supplements showing the spatial distribution of ΔLE, ΔH and $\Delta LE + H$ (see Fig. RspF-1 at the end of the response letter).

- Near line 95, is the reduced shortwave absorption attributed mainly to drying of the atmosphere boundary layer? How about the role of low clouds? I see a clear reduction in low cloud fraction in Fig 1 panel (i) for the tropical case.

  Across all albedo perturbations, the drying of the atmosphere is indeed the main cause of the increased solar insolation. But the reviewer is correct that in the tropical case there is also a reduced cloudiness. This however,

is not necessarily a direct effect of the albedo perturbation but of the subsequent reduction in water availability which in turn led to reduction evapotranspiration. In this section of the manuscript, we would prefer not to discuss the specific aspects of this case but rather at the end of the section discussing the temperature response. Nonetheless to acknowledge that the cloud response does contribute to the change in insolation we included the following at the end of the paragraph:

Importantly, the total reduction in atmospheric moisture content does not necessarily correspond to a decrease in relative humidity, often resulting in a spatially varying cloud response that both amplifies or diminishes the change in surface insolation.

- Section 2.2, again, the sensitivity of global-mean temperature to the location of the perturbation is reminiscent of previous studies (e.g., Zhang et al., 2023; Kaur et al., 2025). While the approaches are slightly different (surface albedo versus TOA solar radiation or solar constant perturbations), the results appear similar.

  We agree with the reviewer that our results align very well with previous studies. Not only do those studies likewise indicate a markedly higher sensitivity to perturbations in high(er) latitudes than in the tropics, but they also identify differences in cloud-cover characteristics—and the associated cloud-feedback sensitivity—as a key driver of the observed variations. Moreover, the fact that the participating models in the LUMIP and IRRMIP projects produce temperature-change patterns that are largely consistent with this spatial variability of sensitivities suggests that this behavior is not confined to the few models employed in the present study and the studies cited above, but represents a robust feature of the climate system.

- Overall, the figures are well prepared, but some panels appear crowded. In Figure 1, for instance, several lines overlap, which reduces readability. It may help to divide the variables into additional panels or adjust the formatting to enhance clarity.

  We thank the reviewer for this helpful observation. Figure 1 indeed appeared to be somewhat crowded, and we have made several targeted revisions to improve readability while preserving the overall layout of the figure (see Fig. RspF-2 at the end of the response letter):

  1. **Panels a–f:** The latitude range displayed has been narrowed so that individual features are more clearly resolved.
  2. **Panels a–f:** The perturbation values are now plotted as semi-transparent scatter points rather than lines, visually separating them from the other plotted quantities to make it clear that they are not an equivalent term in the surface energy balance. Furthermore, we only show non-zero values, preventing the perturbations from obscuring the zero-line.
  3. **Panels g–l:** The previously thick, grey zero-line has been replaced by a thin white line. The line still provides a visual reference but no longer dominates the panel.

  Regarding Fig. 2, we feel that little can be done to improve panel (a). However, in panel (b), we have removed the dots and lines showing TOA SW net + OLHU, which were obscuring the clear-sky TOA LW out signal (see Fig. RspF-2 at the end of the response letter). Given that atmospheric heat uptake is negligible, these two components should perfectly align under a fully closed global energy balance. In the initial version, we included both to demonstrate that energy balance violations due to our hydrological perturbations are present but extremely small. However, since the revised manuscript now directly addresses these violations in the methods section, we believe this visual confirmation is no longer necessary, and thus TOA SW net + OLHU can be omitted without losing important information.

  We believe that these adjustments substantially reduce visual clutter while retaining the scientific content of the original figure and hope the changes meet the reviewer's expectations.

Reviewer #2 (Remarks to the Author):

Key comments:

The nature of the "land surface changes" mentioned in the article is unclear, which leads to the ambiguity of the conclusions drawn in the article.

We agree with the reviewer that is helpful to clarify how the term is used in the present manuscript. In the revised version we now define land-surface change explicitly as the combined effect of land-use change (changes in the management of the land surface such as irrigation) and land-cover change (physical alterations of the surface such as de/afforestation). The definition is introduced at the beginning of the paragraph that discusses the climate impact of these changes, so that readers can immediately understand what is meant by the term throughout the manuscript (see comment on line 45ff below).

The abstract reads like a description of facts or a conclusion, without any scientific data to back it up. It is difficult to assess the reliability of these descriptions.

We thank the reviewer for the helpful observation that the original abstract read more like a set of conclusions than a data-driven summary. To address this we now (a) provide more details on the experimental setup (Earth-system-model simulations with defined perturbations of surface albedo and water availability), (b) explicitly present the key numerical finding that emerges from the simulations (a 0.4 K increase in surface temperature per W m^{-2} change in top-of-atmosphere net short-wave radiation), and (iii) ensure that, after the phrase "we find that" all statements are directly derived from our simulations without including any further references.

A robust proportionality between radiative forcing and global-mean temperature has been established for most climate drivers[13]. However, land-surface changes are considered an exception due to their potential to alter surface hydrology, introducing non-radiative effects that may rival those from surface albedo changes[11,12]. Comparing Earth-system-model simulations with large-scale perturbations of surface albedo and water availability, we find that the spatially integrated biophysical climate impact is similarly dominated by radiative processes and scales approximately linearly with the resulting shifts in planetary albedo, implying a ∼0.4 K surface temperature response per $W\,m^{-2}$ change in top-of-atmosphere net short-wave radiation. Thus, while non-radiative effects strongly influence local temperatures, their net contribution to the global-mean signal is negligible. The planetary-albedo response is primarily governed by regional cloud-cover characteristics, resulting in a substantially stronger temperature response to land-surface change in mid- and high-latitude regions than in the tropics and subtropics.

Line 39: Using the shade of clothing colors to represent the effect of solar radiation is a bit too colloquial and needs to be revised and adjusted.

We thank the reviewer for the helpful suggestion. In order to keep the intuitive tone while avoiding an overly colloquial reference to clothing, we have softened the wording of the albedo example as follows:

Shifts in surface reflectivity modify the absorbed shortwave radiation, which is why dark surfaces often feel hotter on sunny days than bright ones.

Line 45: "the impact of man-made alterations to the terrestrial hydrological cycle on global climate remains unclear to the extent that they constitute the only major anthropogenic driver whose contribution has never fully been quantified in the reports of the Intergovernmental Panel on Climate Change". Carefully reading the content of the paragraph where this sentence appears, it is found that there are several unclear and ambiguous expressions. The sentence "the impact of man-made alterations to the terrestrial hydrological cycle on global climate remains unclear" - what does "man-made" refer to? Has there been no summary on this aspect in the IPCC report? Even the subsequent descriptions cannot effectively support the assumptions and designs in the text.

We appreciate the reviewer's request for clarification. The revised paragraph now:

1. Specifies what "man-made alterations" refer to – we give concrete examples such as agricultural irrigation and changes in evapotranspiration caused by de- or afforestation.

2. Clarifies the statement concerning the state of knowledge in the IPCC reports – “remains poorly constrained” replaces “remains unclear”.
3. Explicitly mentions that the associated non-radiative effects, which complicate the quantification of effects (since this is done with the radiative forcing framework) are thought to be of the same order of magnitude as surface-albedo changes.

Numerous studies have investigated the ways land-surface change – generally understood as the alteration of the characteristics of Earth’s terrestrial surface via land-use and land-cover change – affects climate by modifying these surface properties[14–21]. Yet, the impact of man-made alterations to the surface hydrology – such as agricultural irrigation, or changes in evapotranspiration due to de- or afforestation – on the global climate remains poorly constrained. They constitute the only major anthropogenic driver whose contribution is not fully accounted for in the assessments of the Intergovernmental Panel on Climate Change, owing to difficulties in quantifying associated non-radiative effects, which are thought to be of the same order of magnitude as those of surface-albedo changes[11–13]. One issue may be the expectation that the drivers of local effects, indeed often of a non-radiative nature[22–24], are similarly responsible for the global-scale consequences.

The IPPC has indeed provided a summary on the issue: The fifth assessment report, for example, concluded that: "non-radiative impacts of land use have a similar magnitude, and may be of opposite sign, as the albedo effect (though these are not part of RF)" [11, p. 688], with the special report on Climate And Land adding: "As a consequence, there is low agreement on the sign of the net change in global mean temperature as a result of land use change" [12, p. 138]. AR6 has made an attempt to include the radiative forcing from hydrological shifts due to land-use change, more specifically irrigation, placing it at –0.05 [–0.1 to 0.05] W m^{-2}[13]. However, this evaluation is based on a single study by Sherwood et al.[25], in which the authors added a spatially uniform moisture flux to the lowest atmospheric model level of NCAR’s CESM1. Unfortuately, this omits the local evaporative cooling effect (while maintaining the condensational heating in the atmosphere) and neglects the fact that most irrigated areas are located in the tropics and subtropics. So we believe that "remains poorly constrained" is an adequate description of the present state of knowledge.

What exactly are the perturbations mentioned in the text referring to? Are there any clear and definite contents? How is it manifested? The text mentions "initial perturbation" and "perturbation regions" several times. How exactly are these quantified?

In the original manuscript we describe the perturbations at the end of the introductory section in the following way:

Here, we focus on changes that can be expected to induce a substantial local cooling, namely an increase in albedo of ≈0.15 or a near-complete elimination of moisture constraints on evaporation, with changes being applied to the entire land surface in the latitudes 10°S-10°N, 15°N-35°N and 40°N-60°N, respectively.

, with the technical details being given in the method section. Nonetheless, in the results section we discuss the perturbations in terms of energy perturbations (W m^{-2}) to make them intercomparable with each other and with the terms of the energy balance equation. Unfortunately, we merely mentioned how these energy perturbations are defined in the caption of Fig.1 without giving any details or reasoning why we defined them in this specific way. This has now been corrected and the methods section now includes the following two paragraphs (after the description of the required code modifications):

It should be noted that, in all experiments, the surface properties are partly dynamic because the model determines vegetation, snow, and sea-ice cover interactively. Consequently, the model includes vegetation-related feedbacks with respect to (i) surface albedo, (ii) resistance to evapotranspiration, and (iii) surface roughness. The only feedback that is not represented is the change in atmospheric CO_2 concentrations that would result from vegetation changes, because CO_2 is prescribed in all of our experiments. These feedback effects have important implications for how we define the initial perturbation in terms of energy, to make the perturbation strengths inter-comparable. For surface-albedo experiments we define the initial perturbation as the downwelling short-wave radiation in the unperturbed reference run, multiplied by the prescribed albedo offset. By construction, this definition excludes any albedo effects that arise from changes in vegetation or snow cover. For the water-availability experiments such a straightforward energy definition is problematic due to the way the perturbation is introduced in the model,

that is by modifying the filling of the skin reservoir. Computing the perturbation as "added water x latent heat of vaporisation" would be misleading, since the extra water may be routed to runoff rather than to evaporation. Instead we define the initial perturbation as the difference in latent-heat flux between the perturbed- and the control simulation, evaluated over the perturbed region. In a strict sense, this definition necessarily includes all surface feedbacks – especially those from vegetation – because the moisture flux into the atmosphere is also controlled by leaf area and stomatal conductance. Importantly, however, the amount of water added to the skin reservoir is also determined interactively and responds to the shifts in atmospheric water demand related to changes in transpiration; therefore any vegetation-induced increase or decrease in moisture flux is automatically compensated by the reservoir forcing. In practice this makes the changes in vegetation cover largely irrelevant for the hydrological perturbation.

With respect to the initial perturbation it should also be noted that the experiments were not designed to produce comparable global-mean effects. The three latitudinal bands differ markedly in continental area and in their background-climate states. Consequently, applying the same albedo offset or the same target filling of the skin-reservoir inevitably yields (i) different amounts of water added to the system and (ii) different changes in the global-mean surface albedo. In principle one could (a) adjust the widths of the latitudinal bands so that each band contains a similar land area, (b) restrict the perturbation to a subset of land grid-cells within a band, or (c) adjust the albedo offset and target filling to compensate for the differences in land area and climate within the perturbed regions. However, such "patterned" or size-adjusted perturbations may themselves modify the climate response (e.g., by altering the spatial distribution of the forcing and the associated feedbacks). For this reason we chose the simplest configuration: the same spatially uniform perturbation applied over the entire land surface within equal-width latitudinal bands. We acknowledge that a direct comparison of the absolute temperature responses between the three simulations is therefore of limited meaning. Instead, our analysis emphasizes qualitative differences in the regional responses and the resulting spatial patterns. When we present quantitative results we focus on the proportionality between the spatially integrated driver (e.g., perturbation strength or total change in planetary albedo) and the climate response, thereby removing the first-order effects of differing land-area fractions and background-climate states and enabling a meaningful comparison of the underlying physics across the three latitude bands.

Discussion section: "Our results also help explain why present-day agricultural irrigation is of little consequence for the global mean temperature, despite large amounts of water being redistributed with profound local effects". The author is recommended to conduct more in-depth verification to support the above statement. Existing related studies also suggest that irrigation in farmland has an impact on temperature. For instance, the cooling effect brought about by establishing new irrigation areas on uncultivated land is significantly greater than that in forest and grassland areas.

The only study that we could identify showing a substantial effect of irrigation on global mean temperatures was by Lobel and Duffy from 2006, using NCAR'S CAM3/CLM with a slab-ocean[26]. However, they obtained an irrigation-induced cooling of around -0.55 K only by maintaining soil moisture in all cropland grid cells at saturation levels, while similar studies at the time – which used a more realistic irrigation representation – hardly show any notable global mean response[27–30]. This absence of a global signal is also confirmed by more recent models/model versions, with none of the IRRMIP[31] models producing an average land surface cooling of more than -0.03 K, for a threefold increase of irrigated areas. Thus, we believe that the statement in our manuscript, i.e. a limited global impact despite large local scale effects, is in line with existing global scale studies and we merely clarify the phrasing.

Our results also help resolve another important puzzle: why agricultural irrigation has profound local impacts yet only exerts a minor influence on global mean temperature[27–34].

In the data availability section: It is necessary to provide a description of the data mentioned in the text. Simply mentioning that the data is stored in a certain location is not sufficient.

The reviewer is absolutely right, that some additional information on the primary data facilitates reproducing the results of the present study substantially. Here, we added the following to the data availability statement:

With a few key exceptions, all variables that have been investigated in this study are described in the user manual of MPI-ESM's atmospheric component (ECHAM6[35]) and the technical documentation of its land model (JSBACH3[36]). These documents each contain a list of all output variables, including the information in which data stream they can be found, their GRIB code numbers and units.

There are some derived variables which have to be determined as part of the postprocessing. Some of these variables, such as low-, mid-, and high-altitude cloud fraction can be computed with the ECHAM standard postprocessing, the Afterburner as part of the Climate Data Operators (CDO[37]), a few others have to be computed specifically from the ECHAM output:

1. planetary albedo=-srad0u / srad0d
2. surface albedo:=-sradsu/(srads-sradsu)
3. surface downwelling shortwave radiation=srads-sradsu
4. surface downwelling longwave radiation=trads-tradsu
5. top-of-atmosphere outgoing shortwave radiation=srad0d-srad0
6. Ocean and land heat uptake = srads + trads + ahfs + ahfl

Finally, two additional output fields were implemented in the land model: the change in skin reservoir content resulting from the imposed hydrological perturbation: "skinres_chng_acc", and the unbalanced violation of the water-balance "wb_viol_acc". The first variable is given in kg $m^{-2}s^{-1}$ and was assigned the GRIB code 229, while the second is also given in kg $m^{-2}s^{-1}$ and has the GRIB code 230. Both variables can be found in the models "land" output stream.

Reviewer #2 (Remarks on code availability): By referring to, the guidelines for constructing climate models, the codes used for data processing, and the related explanations can all be viewed through the provided link.

See comment above.

Reviewer #3 (Remarks to the Author):

Specific comments:

Framing of line 95-100 needs to be clear. My understanding is that author's point is about linking increased albedo to surface cooling lowering the saturation vapor pressure that lead to lower atmospheric absorption of solar radiation and eventually leading to increase shortwave surface irradiance. So downward solar radiation increases but albedo effects are stronger and leads to reduce net SW and cooling? But these lines alone do not explain this reasoning well and may even seem counterintuitive to the reader. The authors may also think of having a conceptual figure to link different feedbacks described in the manuscript (also the one with changes in local humidity and counter radiation). These pathways are central to the manuscript and can be more clearly described.

We appreciate the reviewer's feedback and have revised the passage (Lines 95–100) to clarify the causal chain linking albedo increases, cooling, and sw radiation changes. The updated text now presents the processes in a logical sequence and also adds some spatial context on adjacent regions, which may experience increased net sw radiation due to atmospheric drying. The revised passage reads:

The changes in net sw radiation are a function of shifts in surface solar irradiance and -reflectivity, which for albedo increases constitute two opposing factors. Here, the increase in reflectivity reduces net sw radiation at the surface, resulting in general cooling. This cooling lowers the saturation vapor pressure, causing atmospheric drying and thus diminished sw absorption by water vapor, which in turn increases surface solar irradiance (Fig. RspF-2 g-i). Within the perturbed regions, the increase in surface reflectivity dominates, leading to a net reduction in sw radiation. However, because the drying of the atmosphere is not limited to these areas, some adjacent regions can exhibit an increase in net sw radiation.

Lines 86-87: The compensating role of sum of turbulent fluxes (H + LE) is very interesting but at the same time it's not so clear why sensible heat (H) reduces in response to increase evaporation (LE). Net radiation is not strictly constrained and depends on surface temperature (Ts). Therefore, H and LE could, in principle, vary independently through changes in Ts and necessarily need not compensate.

The findings of compensating effect of H and LE are however consistent with some recent works that shows total turbulent fluxes are constrained by radiation and that could explain the compensation (Conte et al., 2019, GRL; Ghausi et al., 2023, PNAS) and also some work on surface flux equilibrium (McColl et al., 2019 JAMES; McColl & Rigden, 2020 GRL).

There are a number of factors contributing to the compensating roles of H and LE. The most straightforward (and important) is availability of energy, as mentioned by the reviewer: Here, the energy deficit due to increased evapotranspiration lowers surface temperatures and with it the sensible heat flux. The second factor is the condensation of water vapor in the atmosphere that necessarily goes along with the increase in LE. Since the sensible heat flux is determined by the surface-atmosphere temperature gradient, H is also reduced by the higher atmospheric temperatures due to an increase in condensational heating – though not necessarily in the same location as the evaporative cooling. So an increase in LE essentially "flattens the vertical temperature profile at both ends", thereby decreasing H. However, the reviewer is absolutely correct that changes in H and LE do not have to fully balance, and in fact they very often don't – as is very prominent in Fig. 1e for the region 15-35N. This is mainly because, the heat transfer between atmosphere and surface is not limited to the turbulent fluxes, and when additional condensational heating warms the atmosphere, a part of the energy is returned to the surface as an increase in lw down (though there are important differences between the responses in different regions; see below). And as long as condensation happens (relativly) low in the atmosphere, most of the energy is returned to the surface (instead of contributing to outgoing lw) either as a net decrease in H or an increase in lw down.

Here, we think that the main contributing factors to the (partial) H-LE compensation can be clarified simply by adding the following sentence to the manuscript:

This compensation arises mainly because evaporative cooling lowers the surface temperature while subsequent condensational heating warms the atmosphere, jointly reducing the surface-atmosphere temperature gradient that drives the sensible heat flux.

Fig 1: It may be good to describe the sign conventions followed in estimating the changes. The surface energy balance can be written in different forms: Rs,net + Rl,net = H+LE+G or Rs,net = Rl,net + H + LE + G based on how the direction of net longwave flux is described. It may be good to mention the form of surface energy balance equation that is used and how the signs of changes /delta EN are estimated (atleast in the figure caption).

We thank the reviewer for highlighting the need for an explicit statement of the sign conventions in Figure 1. The caption has been revised to give the surface-energy-balance equation used in the analysis and define each flux together with its direction. Here, our aim to keep the figure in a way that all positive values show an increase in energy resulted in the slightly unconventional definition:

- SW_{net} positive downward,
- LW_{net} positive downward,
- $H + LE$ positive downward,
- OLHU positive upward.

The updated figure caption now reads:

Panels a-f show 50-year mean, zonally averaged changes in the surface-energy fluxes (EN) resulting from increases in surface albedo (a-c) and water availability (d-f), with perturbed regions indicated on the x-axes. sw net is net surface shortwave radiation [orange], lw net is net surface longwave radiation [red], (H+LE) is the sum of turbulent fluxes [blue], and OLHU is ocean and land heat uptake [brown]. All fluxes are defined as positive downward except OLHU, which is defined as positive upward. Consequently, a positive value in the panels always corresponds to an increase of energy at the surface: 0=sw net+lw net+(H+LE)+OLHU.

Before continuing with the point-by-point response to the reviewer's comments, we would like to emphasize that we fully recognize the validity and importance of the following three questions. In fact, these concern issues that are closely aligned with ongoing work in our group and represent topics we are actively investigating. However, we believe they fall outside the immediate scope of the present study. Addressing them in depth would require substantial additional analysis, data interpretation, and discussion, which would significantly extend the manuscript's length and shift the focus away from its core objectives. Therefore, while we value these points highly, we have chosen not to incorporate them into the current manuscript, but exclusively address them in our response to the reviewers' comments, as we do below.

Fig 1 (a-c): For the albedo changes, while it is clear the reduction in shortwave is mostly balanced by associated decrease in H+LE. However Rl,net seems to have an opposite response in the perturbed region for tropics and mid-latitudes. Why is that?

Here, the situation is rather complex and the lw net response can not be attributed to the decrease in albedo directly. Another striking feature of panel c is that the change in lw net was not only opposing the direction in the other regions, but it was also in the same direction as and even larger than the overall decrease in the causative shortwave radiation. The problem with the tropical albedo perturbation is that it actually triggers a very strong hydrological perturbation where convection is suppressed which reduces precipitation. This, in turn, limits available water (resulting in a substantial die-back of the rain-forest) and thereby evapotranspiration. This secondary perturbation is so strong that, while the global average temperature decreases, temperatures in the perturbed region actually rise. And the latter produces a reduction in lw net that is not only opposing the signal in all other regions but also exceeding the reduction in sw net.

Fig 1 (e): Turbulent fluxes (H+LE) have increased in midlatitudes (15 – 35 N) response to surface water changes but this pattern did not show up in any other region despite increase in counter radiation (Fig -1J). How does individual H and LE fluxes look here? Do they follow a compensation?

Here, the reviewer's observation is correct that the hydrological perturbation in midlatitudes differs from the other regions, in that here the increase in LE is not fully offset by the decrease in H. However, two thirds of the LE increase

are still balanced by the H decrease and we will will provide an additional figure in the supplements to showing the changes in LE and H directly, to clarify that the overall change in the turbulent fluxes in the midlatitudes is still comparatively small, considering the initial increase in LE (see Fig. RspF-1 at the end of our replies). The question why the response in the midlatitudes differs from that in other regions is the subject of an ongoing investigation, but we are happy to summarize our current understanding of the situation: While lapse rate and water vapor feedback are negligible on the global scale they might still play a role in the local/regional scale responses. Here, we think that the decisive factor could be the fact, that the midlatitudes are very dry in comparison to the other two zones. Consequently, the atmospheric water content within the perturbed region actually increases due to the higher water availability. This means that within the perturbed region more incoming, and reflected solar radiation is absorbed by water vapor within the atmosphere, resulting in larger downwelling lw flux. On the global scale this effect would be offset by the drying in the adjacent regions, hence not have a notable effect on the overall signal, but within the perturbed region it could help explain why the sum of the turbulent fluxes shows an increases, despite the reduction in sw net.

Fig 1 (i): Rs-down does not decrease much in the tropics despite similar increase in cloud-cover (%) in other regions. Is it partly compensated by decrease in specific humidity? Lw down also does not respond much to changes in humidity in the tropics.

This is a keen observation and the reviewer is absolutely right that the ratio of cloud cover change to change in sw downwelling radiation differs roughly by a factor of 2 from the other regions. Also, it is correct that this effect appears to be mainly caused by a strong atmospheric drying within the perturbed region, which – in this magnitude – is rather unique to the tropical perturbation. Another contribution stems from the fact that, in the tropics, the effects of hydrological perturbation are still strong at greater altitudes, which is also leading to the change in surface lw down being comparatively small. In the manuscript, our focus lies on low-altitude clouds (since these are by far the most important factor) but when looking at the same ratio for low- and mid-level clouds the discrepancy is much reduced. Another factor is the temporal/spatial distribution of solar insolation. The cloud changes in the two more northern bands occur primarily during the summer months when the insolation is higher in these regions than in the tropics so that that the same amount of clouds block a larger radiative flux. However, even this is not sufficient to fully explain the discrepancy and resolving the latter would require information on the diurnal cycle of cloud formation which, unfortunately, is not available with the monthly output timestep we used for our simulations.

Fig 2: Why does changes in hydrology and albedo has maximum effect on temperature when the changes are made in 15-35N? It will be good to have some discussion around it.

This is because we decided to introduce the perturbation in the most basic way conceivable – the same spatially uniform perturbation applied over the entire land surface in equal-width latitude bands – aiming for a consistency in the perturbation mechanisms rather than similar-magnitude global-scale effects. And, because the bands contain very different extents of land and experience distinct climate regimes, the same local perturbation inevitably results in (i) different volumes of water added to the system and (ii) different magnitudes of change in the (global-mean) surface albedo. Although one could in principle adjust the latitudinal widths so that each band encloses a comparable land area or restrict the perturbation to a subset of grid cells, such "patterned" or size-adjusted experiments could themselves modify the climate response by altering the spatial distribution of the forcing and the associated feedbacks. This however, also means that a direct comparison of the global-mean temperature signal is not necessarily very meaningful. To clarify this, we added the following paragraph to the methods section:

With respect to the initial perturbation it should also be noted that the experiments were not designed to produce comparable global-mean effects. The three latitudinal bands differ markedly in continental area and in their background-climate states. Consequently, applying the same albedo offset or the same target filling of the skin-reservoir inevitably yields (i) different amounts of water added to the system and (ii) different changes in the global-mean surface albedo. In principle one could (a) adjust the widths of the latitudinal bands so that each band contains a similar land area, (b) restrict the perturbation to a subset of land grid-cells within a band, or (c) adjust the albedo offset and target filling to compensate for the differences in land area and climate within the perturbed regions. However, such "patterned" or size-adjusted perturbations may themselves modify the climate response (e.g., by altering the spatial distribution of the forcing and the associated feedbacks). For this reason we chose the simplest configuration: the same spatially uniform perturbation applied over the entire land surface within equal-width

latitudinal bands. We acknowledge that a direct comparison of the absolute temperature responses between the three simulations is therefore of limited meaning. Instead, our analysis emphasizes qualitative differences in the regional responses and the resulting spatial patterns. When we present quantitative results we focus on the proportionality between the spatially integrated driver (e.g., perturbation strength or total change in planetary albedo) and the climate response, thereby removing the first-order effects of differing land-area fractions and background-climate states and enabling a meaningful comparison of the underlying physics across the three latitude bands.

L173-176: It will be good to have a figure supporting it.

We appreciate the reviewer's comment. It's possible we may have misunderstood the request, and we apologize if so. That said, we believe that Figure 3 already serves as the supporting figure for the statements in lines 173–176. The sensitivity parameters used in the analysis were derived from the assumptions outlined in the first paragraph of section 2.3, which were based on the model's behavior as observed in the simulations. Therefore, if those assumptions — particularly regarding the cloud-free fraction and the rate of cloud formation — were inconsistent with the model's dynamics, we would expect a significant mismatch between the simulated values and those predicted using the sensitivity parameters. The good agreement observed supports the plausibility of these assumptions — or at least does not contradict them. We believe this provides sufficient justification for the claims made, and thus consider Figure 3 to be adequate in this context.

L278 – 280: This is a major assumption and shall be mentioned in the discussion section where limitations are listed.

We thank the reviewer for this insightful comment, which rightly highlights that the surface water adjustments described in lines 278–280 represent a significant assumption with implications for mass conservation. Indeed, the initial implementation led to extremely large violations—with artificial fluxes in the 15°N–35°N region far exceeding values of 1 Sv— surpassing the combined discharge of all global rivers and being in the order of magnitude of the freshwater fluxes prescribed in hosing experiments designed to force an AMOC shutdown (albeit with a less targeted spatial distribution than the latter). This prompted us to critically reevaluate and improve the perturbation mechanics, implementing two key modifications: **Dynamic adjustment of skin reservoir fluxes**: Rather than simply adding water whenever the skin reservoir fell below its target threshold, we now explicitly calculate the minimum amount of water required to sustain evaporation at a rate consistent with the target reservoir level. This allows the reservoir to temporarily drop below the threshold, increasing its capacity to retain precipitation and reducing throughfall. As a result, the largest artificial fluxes are now consistently below 1 Sv. **Mass-conserving balancing of fluxes**: We now compensate for water added to the skin reservoir by proportionally reducing locally generated surface runoff and drainage, where possible. To account for the temporal decoupling between these fluxes, we accumulate the mass violations over monthly intervals rather than merely attempting to balance them at each time step. This approach roughly halves the residual mass imbalance, with the majority of the remaining violation manifesting as enhanced precipitation over the ocean rather than river discharge—suggesting this is the practical limit of the method. Importantly, these refinements had minimal impact on our core results, confirming that the model's behavior is largely robust to the specifics of the freshwater perturbation.

Furthermore, adding water to the skin reservoir introduces an energy imbalance in the model, reflecting structural limitations inherent to MPI-ESM (and other CMIP6 models). Here, the fundamental issue lies in how river discharge is handled: instead of explicitly calculating the thermal state of river water, the model assumes that discharge enters ocean grid cells at the ocean's temperature. This simplification means the energy balance isn't strictly closed even in standard configurations, and our perturbation may amplify this pre-existing thermodynamic inconsistency. While this limitation may be significant, it is rarely discussed in Earth system modeling studies as it represents a fundamental compromise in current-generation models. Importantly, with our attempts to balance mass violations, the perturbations remain small relative to total river discharge fluxes and – given that these base model simplifications are considered acceptable for large-scale climate simulations – we assume our additional flux falls within a comparable range of uncertainty.

We have now added a clear discussion of these limitations, explicitly stating the assumptions and their implications. This addition provides full transparency about the physical assumptions underlying our approach and acknowledges their potential impact on the simulated energy budget. We have placed this explanation in the Methods (rather than in the Discussion) because it directly addresses the physical assumptions and technical implementation of our

simulation approach, and it is crucial for readers to understand the validity and limitations of our methodology before interpreting the results.

It should be noted that, for simulations in which water was added to the skin reservoir, we did not fully balance the artificial fluxes, resulting in a marked violation of mass conservation. Here, the initial inputs ranged from $\sim$0.4 Sv ($10^6\,m^3\,s^{-1}$) to $\sim$0.8 Sv, with the largest fluxes occurring in the 15°N–35°N region and the smallest in the 40°N–60°N domain. These rates are not minor modifications to the hydrological cycle but represent severe perturbations akin to those used in freshwater hosing experiments[38]. Therefore, in order to mitigate water balance violations, we attempted to compensate for the added water by reducing locally generated runoff and drainage in the perturbed regions. However, between $\sim$0.2 Sv and $\sim$0.6 Sv of water could not be balanced, of which $\sim$0.05 - $\sim$0.15 Sv entered the ocean via river discharge – though not necessarily within the perturbed latitudinal zones – with potentially important implications for simulated ocean circulation and heat uptake: The perturbations in the 10°S–10°N and 15°N–35°N regions led to a notable weakening of the Atlantic Meridional Overturning Circulation (AMOC), with a reduction of approximately $\sim$2 Sv at 27°N. In contrast, the 40°N–60°N perturbation resulted in a strengthening of the AMOC by a similar magnitude. However, these patterns are not necessarily consistent with the expectable signal for the freshwater forcing alone, which should have lead to a pronounced weakening of the AMOC particularly when freshwater is added in the latitudes that include the regions of Atlantic deep water formation, that is between 40°N–60°N. Instead, the observed AMOC responses align more closely with changes in the latitudinal temperature gradient: cooling in high latitudes can be expected to strengthen the AMOC, while cooling closer to the equator may weaken it. Crucially, the simulations with albedo perturbations exhibited similar AMOC responses despite showing opposing changes in river discharge, suggesting that shifts in the latitudinal temperature gradient – rather than freshwater inputs – may indeed have been the dominant driver of AMOC variability in our experiments. The exception lies in the tropical regions – where hydrological and albedo perturbations produce markedly different AMOC responses – but here, the temperature responses also differ significantly between the two forcing types.

Furthermore, the unbalanced addition of water amplifies the model's inherent inconsistencies in the treatment of the global energy balance. This is not merely a technical oversight but a consequence of deeper structural limitations present in the MPI-ESM framework. Here, river discharge is assumed to enter the ocean grid cell at the temperature of that cell, as the thermal state of water within rivers is never explictly calculated. As a result, the model's energy balance is not strictly closed. By adding or removing water at the surface, our perturbation approach exacerbates this existing thermodynamic inconsistency. However, while the water flux introduced by our adjustments is very large, the additional river runoff remains small relative to the total discharge to the ocean. Given that similar simplifications in the base model are considered acceptable for large-scale climate simulations, we assume that the additional energy flux from our perturbations remains within a comparable range of uncertainty. Nonetheless, we acknowledge that this assumption may not hold in all contexts—particularly at regional scales or over long integration periods—where cumulative effects could become significant.

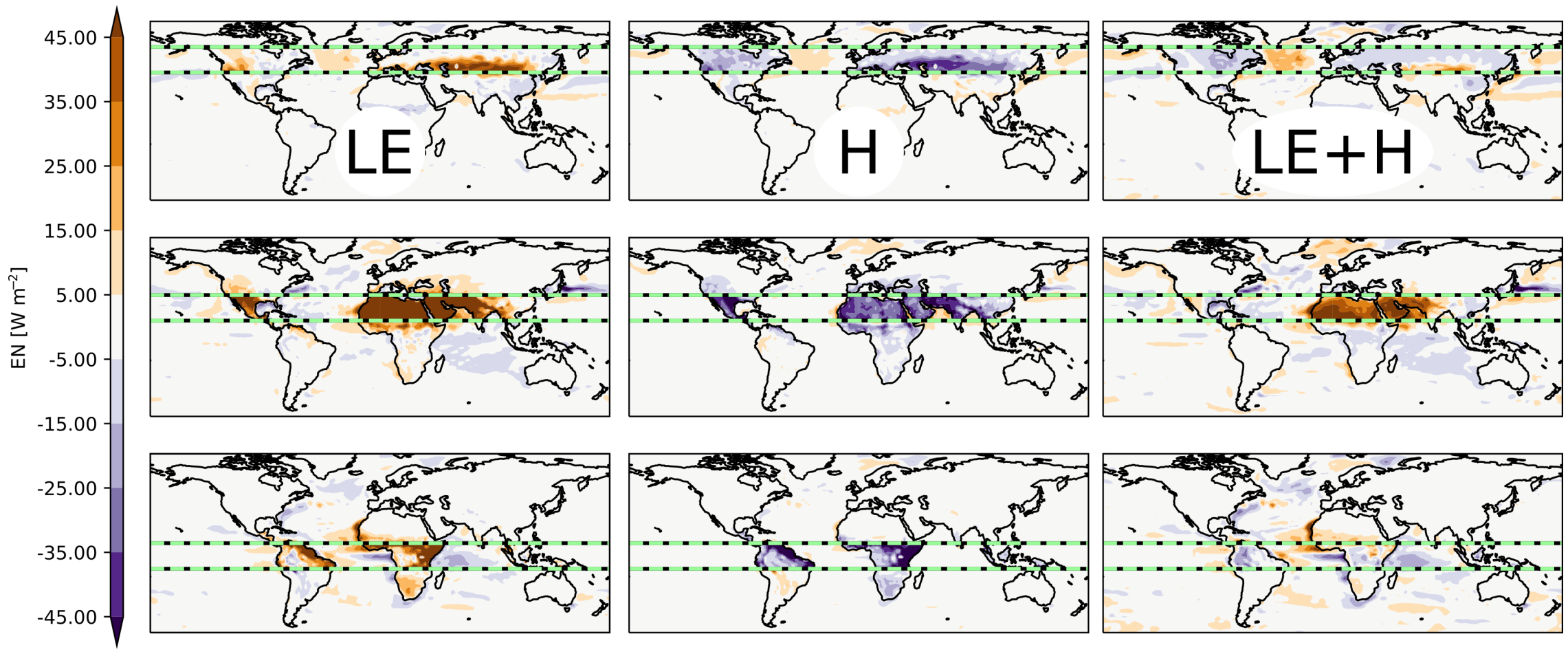

Figure RspF-1. Compensatory shifts in LE and H in case of changes in water availability: Shown are the 50-year mean changes in latent heat flux (LE, first column), sensible heat flux (H, second column) and in the overall turbulent fluxes (LE+H, third column), resulting from an increased water availability in the regions between 40°N-60°N (top row), 15°N-35°N (middle row) and 10°S-10°N (bottom row), with dashed green lines indicating the latitudinal band of the perturbation. Please note that, different from Fig. 1 in the main manuscript, all fluxes are defined as upward postive.

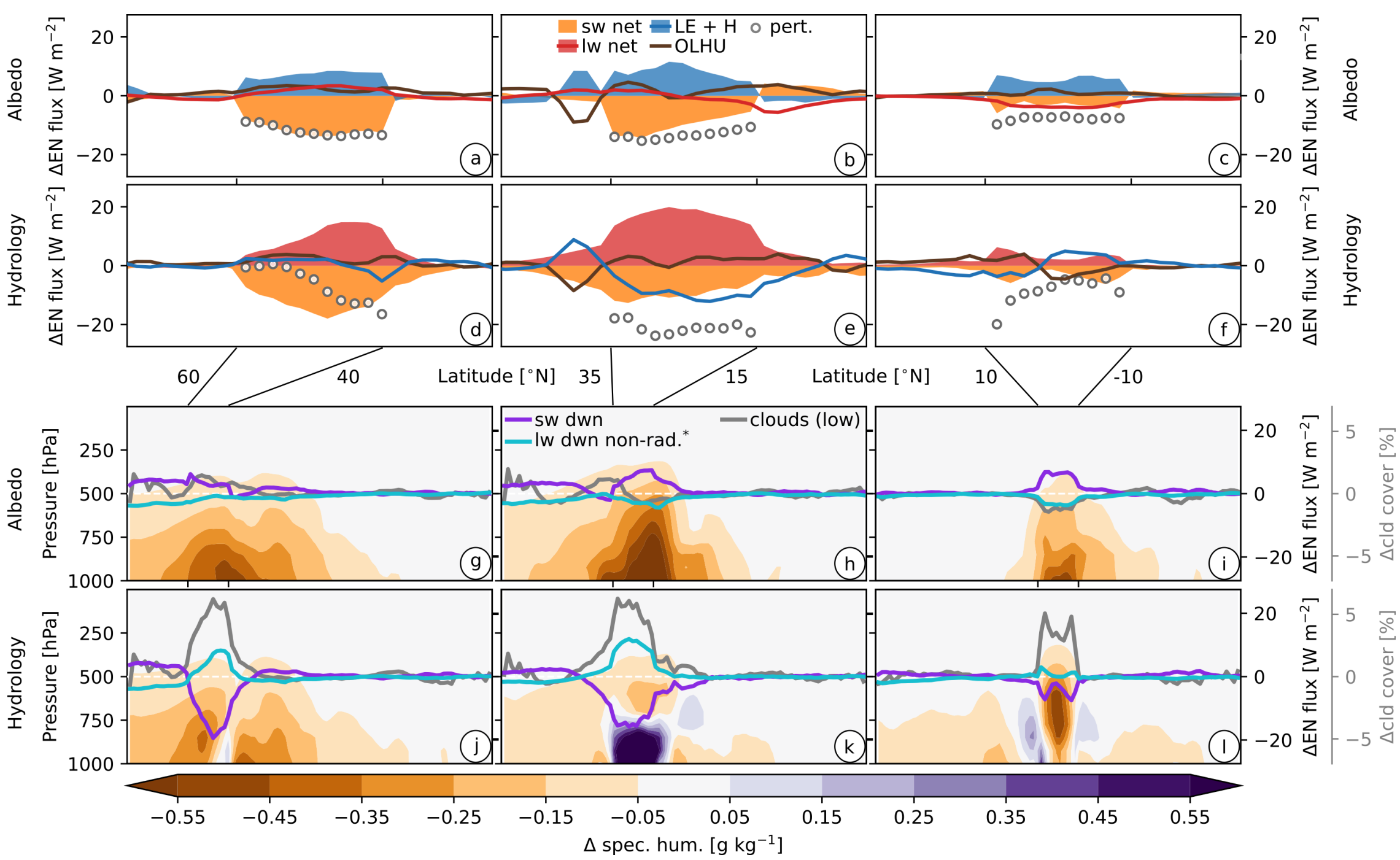

Figure RspF-2. Surface energy balance:

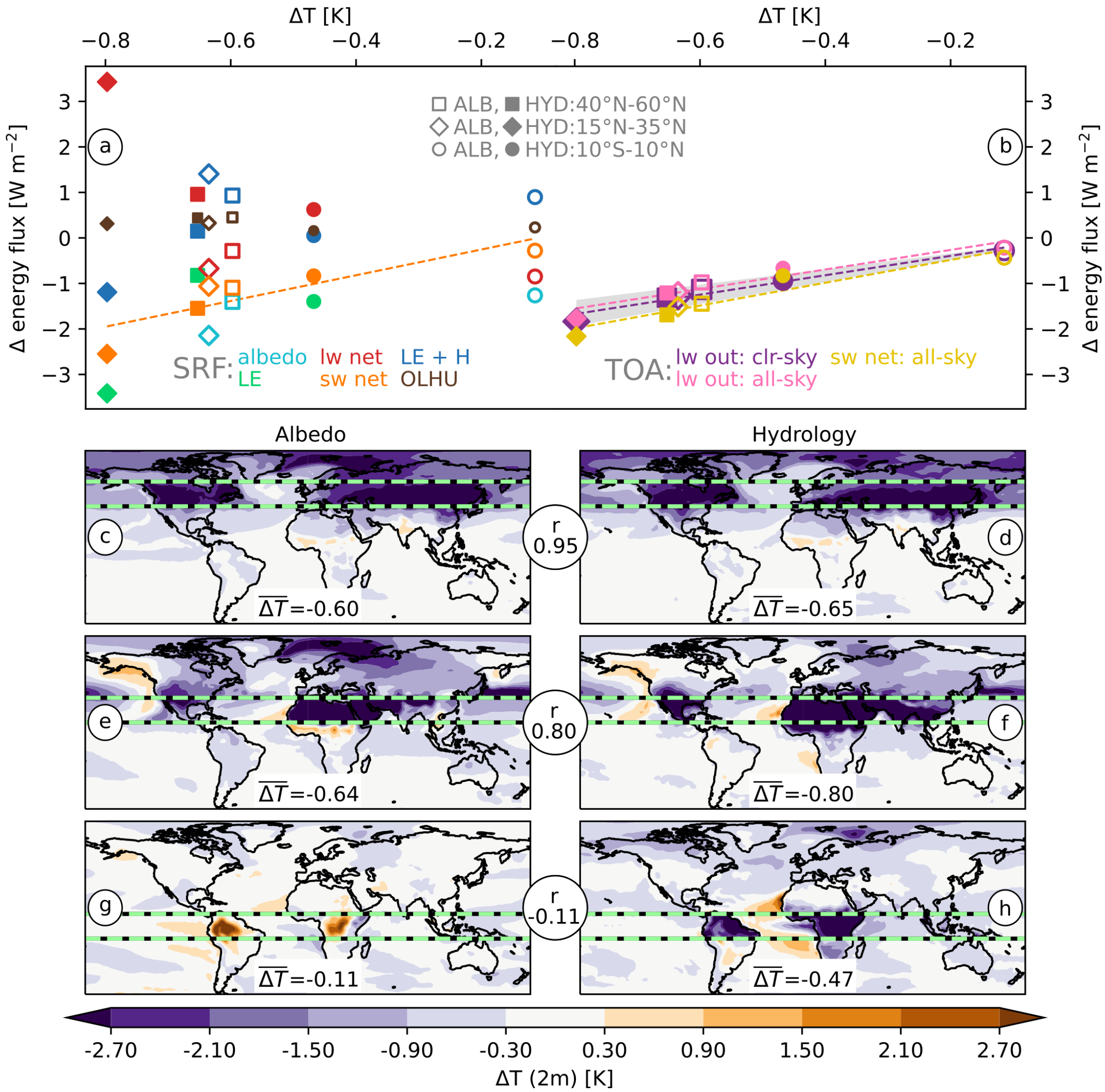

Figure RspF-3. Surface energy balance:

References

1. Koll, D. D. B. & Cronin, T. W. Earth's outgoing longwave radiation linear due to h2o greenhouse effect. *Proceedings of the National Academy of Sciences* **115**, 10293–10298 (2018). URL http://dx.doi.org/10.1073/pnas.1809868115.

2. Feng, J., Paynter, D., Wang, C. & Menzel, R. How atmospheric humidity drives the outgoing longwave radiation–surface temperature relationship and inter-model spread. *Environmental Research Letters* **18**, 104033 (2023). URL http://dx.doi.org/10.1088/1748-9326/acfb98.

3. Laguë, M. M., Swann, A. L. S. & Boos, W. R. Radiative feedbacks on land surface change and associated tropical precipitation shifts. *Journal of Climate* 1–63 (2021). URL http://dx.doi.org/10.1175/JCLI-D-20-0883.1.

4. Laguë, M. M., Quetin, G. R. & Boos, W. R. Reduced terrestrial evaporation increases atmospheric water vapor by generating cloud feedbacks. *Environmental Research Letters* **18**, 074021 (2023). URL http://dx.doi.org/10.1088/1748-9326/acdbe1.

5. Budyko, M. I. The heat balance of the earth's surface. *Soviet Geography* **2**, 3–13 (1961).

6. Manabe, S. & Wetherald, R. T. Thermal equilibrium of the atmosphere with a given distribution of relative humidity. *Journal of the Atmospheric Sciences* **24**, 241–259 (1967). URL http://dx.doi.org/10.1175/1520-0469(1967)024<0241:TEOTAW>2.0.CO;2.
7. Held, I. M. & Suarez, M. J. Simple albedo feedback models of the icecaps. *Tellus* **26**, 613–629 (1974). URL http://dx.doi.org/10.1111/j.2153-3490.1974.tb01641.x.
8. Zhang, B. *et al.* The dependence of climate sensitivity on the meridional distribution of radiative forcing. *Geophysical Research Letters* **50** (2023). URL http://dx.doi.org/10.1029/2023GL105492.
9. Myhre, G., Byrom, R. E., Andrews, T., Forster, P. M. & Smith, C. J. Efficacy of climate forcings in transient cmip6 simulations. *Frontiers in Climate* **6** (2024). URL http://dx.doi.org/10.3389/fclim.2024.1397358.
10. Kauer, H., Bala, G. & Seshadri, A. K. Why is the temperature response larger for radiative forcing imposed in high latitudes than for forcing imposed in low latitudes? *Climate Dynamics* **63** (2025). URL http://dx.doi.org/10.1007/s00382-025-07659-y.
11. IPCC. *Climate Change 2013 - The Physical Science Basis: Working Group I Contribution to the Fifth Assessment Report of the Intergovernmental Panel on Climate Change* (Cambridge University Press, Cambridge, United Kingdom and New York, NY, USA, 2013). URL http://www.climatechange2013.org/images/report/WG1AR5_ALL_FINAL.pdf.
12. IPCC. *Climate Change and Land: IPCC Special Report on Climate Change, Desertification, Land Degradation, Sustainable Land Management, Food Security, and Greenhouse Gas Fluxes in Terrestrial Ecosystems* (Cambridge University Press, 2022). URL http://dx.doi.org/10.1017/9781009157988.
13. IPCC. *Climate Change 2021 – The Physical Science Basis: Working Group I Contribution to the Sixth Assessment Report of the Intergovernmental Panel on Climate Change* (Cambridge University Press, 2023). URL http://dx.doi.org/10.1017/9781009157896.
14. Pitman, A. J. *et al.* Uncertainties in climate responses to past land cover change: First results from the lucid intercomparison study. *Geophysical Research Letters* **36** (2009). URL http://dx.doi.org/10.1029/2009GL039076.
15. Lawrence, P. J. & Chase, T. N. Investigating the climate impacts of global land cover change in the community climate system model. *International Journal of Climatology* **30**, 2066–2087 (2010). URL http://dx.doi.org/10.1002/joc.2061.
16. Swann, A. L. S., Fung, I. Y. & Chiang, J. C. H. Mid-latitude afforestation shifts general circulation and tropical precipitation. *Proceedings of the National Academy of Sciences* **109**, 712–716 (2011). URL http://dx.doi.org/10.1073/pnas.1116706108.
17. Pongratz, J. *et al.* Land use effects on climate: Current state, recent progress, and emerging topics. *Current Climate Change Reports* **7**, 99–120 (2021). URL http://dx.doi.org/10.1007/s40641-021-00178-y.
18. De Hertog, S. J. *et al.* The biogeophysical effects of idealized land cover and land management changes in earth system models. *Earth System Dynamics* **13**, 1305–1350 (2022). URL http://dx.doi.org/10.5194/esd-13-1305-2022.
19. Lawrence, D., Coe, M., Walker, W., Verchot, L. & Vandecar, K. The unseen effects of deforestation: Biophysical effects on climate. *Frontiers in Forests and Global Change* **5** (2022). URL http://dx.doi.org/10.3389/ffgc.2022.756115.
20. Liu, S. *et al.* Local and non-local biophysical impacts of deforestation on global temperature during boreal summer: Cmip6-lumip multimodel analysis. *Journal of Geophysical Research: Atmospheres* **128** (2023). URL http://dx.doi.org/10.1029/2022JD038229.
21. Luo, H., Quaas, J. & Han, Y. Decreased cloud cover partially offsets the cooling effects of surface albedo change due to deforestation. *Nature Communications* **15** (2024). URL http://dx.doi.org/10.1038/s41467-024-51783-y.
22. Bright, R. M. *et al.* Local temperature response to land cover and management change driven by non-radiative processes. *Nature Climate Change* **7**, 296–302 (2017). URL http://dx.doi.org/10.1038/nclimate3250.
23. Zhang, G., Li, H., Li, J., Meng, C. & Cai, X. Non-radiative effect of land use/land cover differences on land surface temperature simulated by noah-mp land surface model over east asia. *Journal of Geophysical Research: Atmospheres* **128** (2023). URL http://dx.doi.org/10.1029/2022JD037722.

24. Hänchen, L., Quesada, B. & Arneth, A. Non-radiative effects dominate the local surface temperature response to land-cover change—insights from a semi-empirical model. *Journal of Environmental Management* **380**, 124741 (2025). URL http://dx.doi.org/10.1016/j.jenvman.2025.124741.

25. Sherwood, S. C., Dixit, V. & Salomez, C. The global warming potential of near-surface emitted water vapour. *Environmental Research Letters* **13**, 104006 (2018). URL http://dx.doi.org/10.1088/1748-9326/aae018.

26. Lobell, D., Bala, G. & Duffy, P. Biogeophysical impacts of cropland management changes on climate. *Geophys. Res. Lett.* **33** (2006).

27. Sacks, W. J., Cook, B. I., Buenning, N., Levis, S. & Helkowski, J. H. Effects of global irrigation on the near-surface climate. *Clim. Dyn.* **33**, 159–175 (2009).

28. Puma, M. J. & Cook, B. I. Effects of irrigation on global climate during the 20th century. *J. Geophys. Res. [Atmos.]* **115** (2010).

29. de Vrese, P. & Hagemann, S. Uncertainties in modelling the climate impact of irrigation. *Climate Dynamics* **51**, 2023–2038 (2017). URL https://doi.org/10.1007/s00382-017-3996-z.

30. Thiery, W. *et al.* Present-day irrigation mitigates heat extremes. *Journal of Geophysical Research: Atmospheres* **122**, 1403–1422 (2017). URL https://doi.org/10.1002/2016jd025740.

31. Yao, Y. *et al.* Impacts of irrigation expansion on moist-heat stress based on irrmip results. *Nature Communications* **16** (2025). URL http://dx.doi.org/10.1038/s41467-025-56356-1.

32. Chen, L. & Dirmeyer, P. A. Global observed and modelled impacts of irrigation on surface temperature. *International Journal of Climatology* **39**, 2587–2600 (2019). URL http://dx.doi.org/10.1002/joc.5973.

33. Gormley-Gallagher, A. M. *et al.* Agricultural management effects on mean and extreme temperature trends. *Earth System Dynamics* **13**, 419–438 (2022). URL http://dx.doi.org/10.5194/esd-13-419-2022.

34. McDermid, S. *et al.* Irrigation in the earth system. *Nature Reviews EarthI& ; Environment* **4**, 435–453 (2023). URL http://dx.doi.org/10.1038/s43017-023-00438-5.

35. Rast, S. *et al.* User manual for echam6 - june 21, 2013, (2013-02-26), version echam-6.1.06p3-guide-1.3 (2013). URL http://pubman.mpdl.mpg.de/pubman/item/escidoc:1810486.

36. Reick, C. H. *et al.* Jsbach 3 - the land component of the mpi earth system model: documentation of version 3.2. *Berichte zur Erdsystemforschung* **240** (2021). URL https://pure.mpg.de/pubman/item/item_3279802.

37. Schulzweida, U. Cdo user guide (2022). URL https://zenodo.org/record/7112925.

38. Jackson, L. C. *et al.* Understanding amoc stability: the north atlantic hosing model intercomparison project. *Geoscientific Model Development* **16**, 1975–1995 (2023). URL http://dx.doi.org/10.5194/gmd-16-1975-2023.

Replies to reviewer comments on:
"Global climate impact of land-surface change driven by radiative effects"

Dear Reviewers,

please find below our responses to your comments on our manuscript. Here, we repeat the comments in red letters, our response is given in black and passages that are included in the revised manuscript are given in blue.

We would like to sincerely thank all three reviewers for their second reading of our manuscript and for their helpful suggestions. We appreciate that the paper is not necessarily the easiest or most straightforward to read, and we are grateful for the reviewers' willingness to stay with us throughout the process.

Yours sincerely,
The Authors

Reviewer #1 (Remarks to the Author):

I would like to thank the authors for addressing my previous comments. I believe the manuscript is now in good shape and can be published after the editorial process.

Reviewer #2 (Remarks to the Author):

How can the reliability of the obtained results be demonstrated in the text? There are no relevant considerations in the text.Are there any other reliable simulation models that present different results?

We sincerely thank the reviewer for their insightful comment. We are not aware of any study whose findings fundamentally contradict the results reported here. In fact, key aspects of our findings are consistent with recent studies, particularly regarding the response to hydrological perturbations—effects that are arguably the most counterintuitive in our analysis. These results closely align with those reported by Lague et al.[1,2], who employed a very similar experimental design within a simplified CESM framework. Furthermore, the findings from Doutriaux-Boucher et al.[3], mentioned by reviewer #1 in the first review, based on the Met Office Unified Model System, are consistent with surface hydrology influencing climate primarily through shifts in low cloud cover—supporting the physical plausibility of our results. However, these studies also rely on a single model, and neither includes a direct, systematic comparison between albedo and hydrological effects. To the best of our knowledge, no existing multi-model ensemble with a consistent experimental setup currently enables a direct comparison of surface perturbations of different types across multiple models. Consequently, we are unaware of any other reliable simulation dataset that presents results directly comparable to ours. Given the absence of such directly comparable simulations, we assessed our results against output from the IRRMIP and LUMIP model intercomparison projects, which represent some of the largest coordinated efforts to date specifically addressing large-scale land-surface changes—hydrological perturbations in IRRMIP, and combined albedo and hydrological changes in LUMIP. While substantial inter-model spread in cloud sensitivity is evident, the overall globally averaged temperature and radiative responses are qualitatively consistent with our findings. This convergence across independent model ensembles, despite differing experimental designs and model structures, strongly supports the robustness of our conclusions, even in the face of model uncertainty. We believe this evaluation is already reflected in the manuscript, and we therefore did not propose any changes to the latter.

Reviewer #3 (Remarks to the Author):

The authors have adequately addressed most of my previous comments and concerns. For the comments related to the latitudinal patterns in Figure 1, I understand that the authors consider these questions to be beyond the scope of the present study and part of their ongoing research. However, I think it would be helpful to briefly mention this somewhere in the manuscript as a topic for future work. This would make it clear to the readers that these aspects are not yet fully understood.

We sincerely thank the reviewer for their final comment and we fully agree that these regional complexities — which are part of ongoing research — should be acknowledged in the manuscript. We have added a sentence in the Results section (after the discussion of local-scale effects) to highlight that while these processes introduce distinct zonally averaged patterns, they do not lead to a significant global-scale response. This placement ensures the point is contextually relevant and maintains the manuscript's focus.

At the local level, changes in surface hydrology have a crucial non-radiative impact on the lw-TOARSTRs via unique lapse-rate effects (supplements SF7). Furthermore, there are important regional differences in the portioning of turbulent fluxes and long- and shortwave radiation — introducing distinct patterns in the zonally averaged response to surface perturbations which are currently being investigated in ongoing work. At the global level, however, the similarity to the lw-TOARSTRs for albedo changes – an entirely radiative disturbance – supports our initial hypothesis, suggesting that such non-radiative effects even out on this scale and play no major role in the overall climate impact of hydrological perturbations. Instead, a similar consistency in the sw-TOARSTRs – on average 2.5 $\mathrm{W\,m^{-2}\,K^{-1}}$ – implies that the spatially integrated response to any large-scale change in the physical land-surface properties is driven by changes in planetary albedo, with the ocean heat uptake being the only other factor notably affecting the temperature signal (as indicated by the differences between lw- and sw-TOARSTRs).

References

1. Laguë, M. M., Swann, A. L. S. & Boos, W. R. Radiative feedbacks on land surface change and associated tropical precipitation shifts. *Journal of Climate* 1–63 (2021). URL http://dx.doi.org/10.1175/JCLI-D-20-0883.1.
2. Laguë, M. M., Quetin, G. R. & Boos, W. R. Reduced terrestrial evaporation increases atmospheric water vapor by generating cloud feedbacks. *Environmental Research Letters* **18**, 074021 (2023). URL http://dx.doi.org/10.1088/1748-9326/acdbe1.
3. Doutriaux-Boucher, M., Webb, M. J., Gregory, J. M. & Boucher, O. Carbon dioxide induced stomatal closure increases radiative forcing via a rapid reduction in low cloud. *Geophysical Research Letters* **36** (2009). URL http://dx.doi.org/10.1029/2008GL036273.
